# Panorama des pathologies infectieuses et non infectieuses de Guyane en 2022

**DOI:** 10.48327/mtsi.v3i1.2023.308

**Published:** 2023-02-17

**Authors:** Loïc Epelboin, Philippe Abboud, Karim Abdelmoumen, Frédégonde About, Antoine Adenis, Théo Blaise, Romain Blaizot, Timothée Bonifay, Morgane Bourne-Watrin, Mathilde Boutrou, Gabriel Carles, Pierre-Yves Carlier, Jean-François Carod, Luisiane Carvalho, Pierre Couppié, Bertrand De Toffol, François Delon, Magalie Demar, Justin Destoop, Maylis Douine, Jean-Pierre Droz, Narcisse Elenga, Antoine Enfissi, Yves-Kénol Franck, Alexis Fremery, Mélanie Gaillet, Hatem Kallel, Arsène Amadouhé Kpangon, Anne Lavergne, Paul Le Turnier, Lucas Maisonobe, Céline Michaud, Rémi Mutricy, Mathieu Nacher, Richard Naldjinan-Kodbaye, Margot Oberlis, Guillaume Odonne, Lindsay Osei, Jean Pujo, Sébastien Rabier, Brigitte Roman-Laverdure, Cyril Rousseau, Dominique Rousset, Nadia Sabbah, Vincent Sainte-Rose, Roxane Schaub, Karamba Sylla, Marc-Alexandre Tareau, Victor Tertre, Camille Thorey, Véronique Vialette, Gaëlle Walter, Magaly Zappa, Félix Djossou, Nicolas Vignier

**Affiliations:** 1 Unité des maladies infectieuses et tropicales, Centre hospitalier de Cayenne, Cayenne, Guyane; 2 Centre d'investigation clinique Guyane (Inserm CIC 1424), Centre hospitalier de Cayenne, Cayenne, Guyane; 3 Département des maladies infectieuses, Centre hospitalier de Mayotte, Mamoudzou, Mayotte; 4 Unité carcérale de soins ambulatoires, Centre hospitalier de Cayenne, Cayenne, Guyane; 5 Service de dermatologie, Centre hospitalier de Cayenne, Cayenne, Guyane; 6 Service de gynécologie-obstétrique, Centre hospitalier de l'ouest guyanais, Saint-Laurent-du-Maroni, Guyane; 7 Laboratoire de biologie médicale, Centre hospitalier de l'ouest guyanais, Saint-Laurent-du-Maroni, Guyane; 8 Agence régionale de santé de Guyane, Cayenne, Guyane; 9 Santé publique France, Cayenne, Guyane; 10 Service de neurologie, Centre hospitalier de Cayenne, Cayenne, Guyane; 11 TBIP (Tropical Biome and ImmunoPhysiopathology), Université de Guyane, Cayenne, Guyane; 12 Laboratoire hospitalo-universitaire de parasitologie et mycologie, Centre hospitalier de Cayenne Andrée-Rosemon, Cayenne, Guyane; 13 Université Claude Bernard Lyon 1 et Centre Léon Bérard, Lyon, France; 14 Service de pédiatrie, Centre hospitalier de Cayenne, Cayenne, Guyane; 15 Laboratoire de virologie, Institut Pasteur de la Guyane; 16 Service de cardiologie, Centre hospitalier de Cayenne, Cayenne, Guyane; 17 Service d'accueil des urgences et SAMU, Centre hospitalier de Cayenne, Cayenne, Guyane; 18 Pôle des Centres délocalisés de prévention et de soins, Centre hospitalier de Cayenne, Cayenne, Guyane; 19 Service de réanimation, Centre hospitalier de Cayenne, Cayenne, Guyane; 20 Service de médecine, Centre hospitalier de Kourou, Kourou, Guyane; 21 Laboratoire des interactions virus-hôtes, Institut Pasteur de la Guyane, Cayenne, Guyane; 22 Croix-Rouge française de Guyane, Cayenne, Guyane; 23 Laboratoire Écologie, évolution, interactions des systèmes amazoniens (LEEISA), CNRS, Université de Guyane, IFREMER, Cayenne, Guyane; 24 COREVIH (Comité de coordination de la lutte contre les infections sexuellement transmissibles et le virus de l'immunodéficience humaine), Centre hospitalier de Cayenne, Cayenne, Guyane; 25 Service d'endocrinologie-diabétologie et maladies métaboliques, Centre hospitalier de Cayenne, Cayenne, Guyane; 26 Service de médecine, Centre hospitalier de l'ouest guyanais, Saint-Laurent-du-Maroni, Guyane; 27 Direction interarmées du service de santé (DIASS); 28 Laboratoire Eurofins Guyane, site de Kourou, Centre hospitalier de Kourou, Guyane; 29 Service de radiologie, Centre hospitalier de Cayenne, Cayenne, Guyane

**Keywords:** Guyane, Amérique du sud, Médecine des voyages, Médecine tropicale, Épidémiologie, French Guiana, South America, Travel medicine, Tropical medicine, Epidemiology

## Abstract

Source de nombreux mythes, la Guyane représente un territoire exceptionnel par la richesse de sa biodiversité et par la variété des communautés qui la composent. Seul territoire européen en Amazonie, entouré du géant brésilien et du méconnu Suriname, on y lance des fusées Ariane 6 depuis Kourou tandis que 50% de la population vit en dessous du seuil de pauvreté. Cette situation paradoxale est source de problématiques de santé spécifiques à ce territoire, qu'il s'agisse de maladies infectieuses à germes méconnus, d'intoxications, ou de pathologies chroniques.

Certaines maladies infectieuses telles que la fièvre Q, la toxoplasmose, la cryptococcose ou l'infection à VIH sont communes aux pays tempérés, mais présentent en Guyane des spécificités entraînant une prise en charge et un raisonnement médical parfois différents. Parallèlement à ces pathologies, de nombreuses maladies tropicales sont par ailleurs présentes sur un mode endémique et / ou épidémique telles que le paludisme, la leishmaniose, la maladie de Chagas, l'histoplasmose ou la dengue.

De plus, la dermatologie amazonienne est extrêmement variée, allant de pathologies rares, mais graves (ulcère de Buruli, lèpre), à d'autres fréquentes et bénignes telles que les poux d'agouti (acariens de la famille des Trombiculidae) ou la papillonite. Les envenimations par la faune sauvage ne sont pas rares, et méritent une prise en charge appropriée au taxon incriminé. Les pathologies obstétricale, cardiovasculaire et métabolique cosmopolites prennent parfois en Guyane une dimension particulière à prendre en compte dans la prise en charge des patients. Enfin, différents types d'intoxication sont à connaître par les praticiens, notamment aux métaux lourds.

Les ressources de niveau européen offrent des possibilités diagnostiques et thérapeutiques inexistantes dans les pays et régions des environs, permettant ainsi la prise en charge de maladies peu connues ailleurs.

Du fait de ces mêmes ressources de niveau européen, la recherche en Guyane occupe une place clé au sein de la région amazonienne, malgré une population moins nombreuse que dans les pays alentour. Ainsi, certaines pathologies telles que l'histoplasmose du patient immunodéprimé, la toxoplasmose amazonienne ou la fièvre Q ne sont pratiquement pas décrites dans les pays voisins, probablement du fait d'un sous-diagnostic lié à des ressources plus limitées. La Guyane joue ainsi un rôle moteur dans l’étude de ces pathologies.

L'objectif de ce panorama est d'orienter les soignants venant ou exerçant en Guyane dans leur pratique quotidienne, mais également les praticiens prenant en charge des personnes au retour de Guyane.

## Abstract (en)

Source of many myths, French Guiana represents an exceptional territory due to the richness of its biodiversity and the variety of its communities. The only European territory in Amazonia, surrounded by the Brazilian giant and the little-known Suriname, Ariane 6 rockets are launched from Kourou while 50% of the population lives below the poverty line. This paradoxical situation is a source of health problems specific to this territory, whether they be infectious diseases with unknown germs, intoxications or chronic pathologies.

Some infectious diseases such as Q fever, toxoplasmosis, cryptococcosis or HIV infection are in common with temperate countries, but present specificities leading to sometimes different management and medical reasoning. In addition to these pathologies, many tropical diseases are present in an endemic and / or epidemic mode such as malaria, leishmaniasis, Chagas disease, histoplasmosis or dengue. Besides, Amazonian dermatology is extremely varied, ranging from rare but serious pathologies (Buruli ulcer, leprosy) to others which are frequent and benign such as agouti lice (mites of the family Trombiculidae) or papillonitis. Envenomations by wild fauna are not rare, and deserve an appropriate management of the incriminated taxon. Obstetrical, cardiovascular and metabolic cosmopolitan pathologies sometimes take on a particular dimension in French Guiana that must be taken into account in the management of patients. Finally, different types of intoxication are to be known by practitioners, especially due to heavy metals.

European-level resources offer diagnostic and therapeutic possibilities that do not exist in the surrounding countries and regions, thus allowing the management of diseases that are not well known elsewhere.

Thanks to these same European-level resources, research in Guyana occupies a key place within the Amazon region, despite a smaller population than in the surrounding countries. Thus, certain pathologies such as histoplasmosis of the immunocompromised patient, Amazonian toxoplasmosis or Q fever are hardly described in neighboring countries, probably due to under-diagnosis linked to more limited resources. French Guiana plays a leading role in the study of these diseases.

The objective of this overview is to guide health care providers coming to or practicing in French Guiana in their daily practice, but also practitioners taking care of people returning from French Guiana.

## Abréviations

**Table:** 

Ae	*Aedes*
ARN	Acide ribonucléique
ARS	Agence régionale de santé
ARV	Antirétroviral
ATAR	Antenne de traitement antirabique
ATL	Leucémie / Lymphome à cellules T de l'adulte (Adult T-cell leukemia / lymphoma)
ATU	Autorisation temporaire d'utilisation
AVC	Accident vasculaire cérébral
BAAR	Bacilles acido-alcoolo-résistants
BCG	Bacille de Calmette et Guérin
BTP	Bâtiment et travaux publics
Cb	*Coxiella burnetii*
CDPS	Centres délocalisés de prévention et de soins
CH	Centre hospitalier
CHC	Centre hospitalier de Cayenne
CHIKV	Virus chikungunya
CHK	Centre hospitalier de Kourou
CHOG	Centre hospitalier de l'ouest guyanais
CHRU	Centre hospitalier régional universitaire
CID	Centre intégré de la drépanocytose
CNR	Centre national de référence
Corevih	Comité de coordination de la lutte contre les infections sexuellement transmissibles et le VIH
COVID-19	Maladie à coronavirus 2019
CRP	Protéine C-réactive
CTAR	Centre de traitement antirabique
DENV	Virus de la dengue
DHBN	Dermohypodermites bactériennes nécrosantes
DHBNN	Dermohypodermites bactériennes non nécrosantes
DTP	Vaccination contre la diphtérie, le tétanos et la poliomyélite
EBV	Virus d'Epstein-Barr
ECSA	Chikungunya, lignée East / Central / South Africa (Afrique de l'Est / Centrale / du Sud)
FJ	Fièvre jaune
G6PD	Glucose-6-phosphate déshydrogénase
GM-CSF	Facteur de stimulation des colonies de granulocytes et de macrophages (Granulocyte-macrophage colony-stimulating factor)
HHV8	Herpèsvirus humain type 8
HPS	Syndrome pulmonaire à hantavirus
HPV	Papillomavirus humains
HTA	Hypertension artérielle
HTLV-1	Virus T-lymphotropique humain type 1 (Human T-lymphotropic virus type 1)
IFNγ	Interféron gamma
IgM	Immunoglobine M
INCa	Institut national du cancer
IP	Indice parasitaire
IST	Infection sexuellement transmissible
IV	Intraveineux
LC	Leishmaniose cutanée
LDH	Lactate déshydrogénase
LNH	Lymphome non Hodgkinien
MAC	*Mycobacterium avium intracellulare*
MAYV	Virus Mayaro
MBNT	Mycobactérie non tuberculeuse
MBNTER	Mycobactérie non tuberculeuse d'expression respiratoire
MGG	May-Grünwald Giemsa (coloration)
MST	Microsatellites
NPA	Nodule pénien artificiel
OFAST	Office anti-stupéfiants
OFDT	Observatoire français des drogues et toxicomanies
OMS	Organisation mondiale de la santé
OROV	Virus Oropouche
PAC	Pneumonies aiguës communautaires
PCR	Réaction de polymérisation en chaîne (Polymerase Chain Reaction)
PVVIH	Personne vivant avec le VIH
RABV	Virus de la rage (Rabies virus)
ROR	Vaccin rougeole-oreillons-rubéole
SAMU	Service d'aide médicale urgente
SARS-CoV-2	Coronavirus-2 responsable du syndrome respiratoire aigu sévère (Severe acute respiratory syndrome coronavirus-2)
SCA	Syndrome coronarien aigu
SCA ST+	Syndrome coronarien aigu avec sus-décalage du segment ST
SDRA	Syndrome de détresse respiratoire aiguë
SIDA	Syndrome d'immunodéficience acquise
SMUR	Structures mobiles d'urgence et de réanimation
SNC	Système nerveux central
TI	Taux d'infestation
TONV	Virus Tonate
TSP / HAM	Paraparésie spastique tropicale ou Myélopathie associée à HTLV-1 (Tropical spastic paraparesis / HTLV-1 associated myelopathy)
UB	Ulcère de Buruli
VHA	Virus de l'hépatite A
VHB	Virus de l'hépatite B
VHC	Virus de l'hépatite C
VHE	Virus de l'hépatite E
VIH	Virus de l'immunodéficience humaine
VS AV	Véhicule de secours et d'assistance aux victimes
WHO	World health organization
YFV	Yellow fever virus / Fièvre jaune (FJ)
ZIKV	Virus Zika

## Préambule

L'objectif de ce panorama est de présenter de façon large les pathologies tropicales infectieuses et non infectieuses les plus fréquentes et/ou les plus originales de Guyane, et de partager cette expérience avec les professionnels de santé d'ici et d'ailleurs.

Les chapitres sont répartis ainsi: les pathologies infectieuses dites fébriles regroupant des infections bactériennes, virales, parasitaires et fongiques, la dermatologie tropicale, les pathologies tropicales non infectieuses incluant les intoxications, les envenimations, et certaines pathologies spécifiques telles que les hémoglobinopathies et le béribéri. Un chapitre sur des phénomènes spécifiques à la Guyane est proposé, puis des paragraphes concernant les particularités guyanaises de la prise en charge de la grossesse et des pathologies cardiovasculaires et métaboliques. Enfin, des conseils aux voyageurs sont proposés ainsi que trois algorithmes décisionnels synthétiques.

## Contexte

### Géographie et biodiversité

Loïc Epelboin

**Figure 1:**
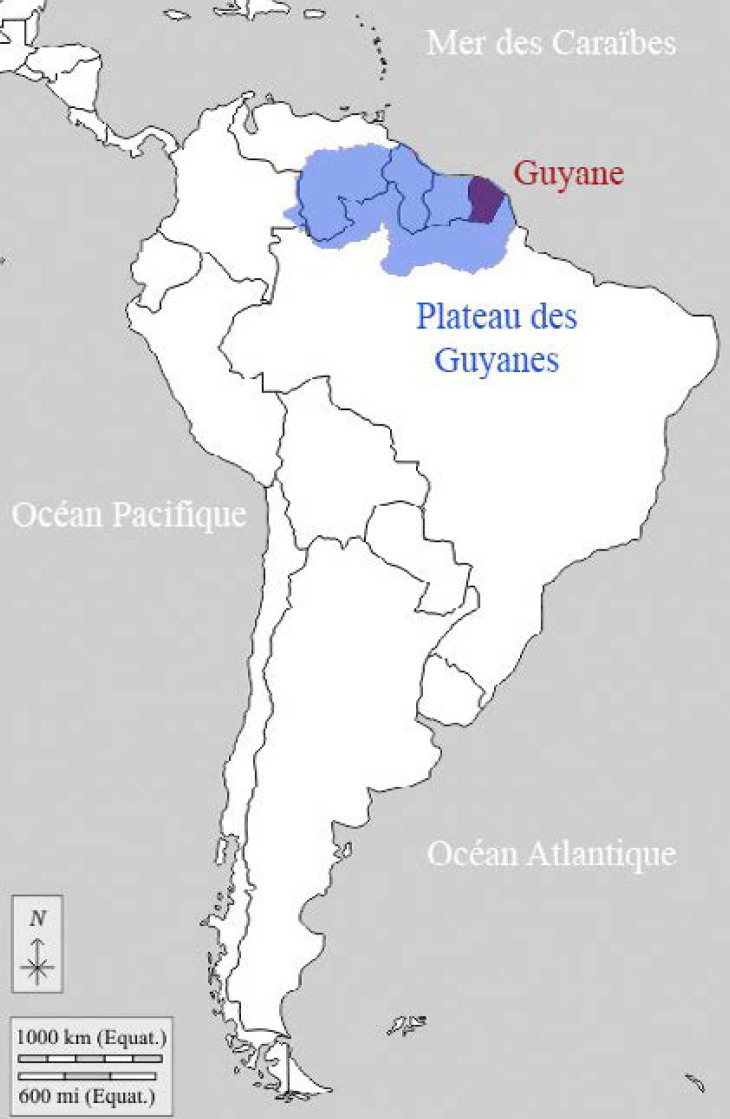
Situation géographique de la Guyane (source: d-maps.com/carte.php?num_car=5106) Geographical situation of French Guiana (source: d-maps.com/carte.php?num_car=5106)

**Figure 2:**
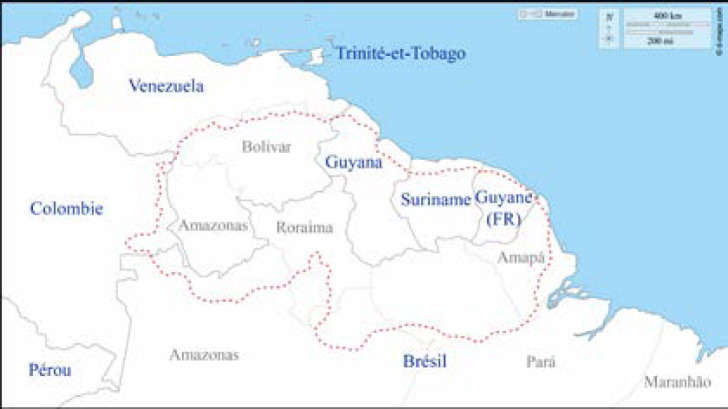
Carte du plateau des Guyanes (source: d-maps.com/carte.php?num_car=284548">https://d-maps.com/carte.php?num_car=284548">d-maps.com/carte.php?num_car=284548) Légende: Les pointillés rouges montrent la délimitation de l'aire géologique. Historiquement: États Amazonas et Bolivar du Venezuela et départements de Vichada et Guainía de Colombie (ex-Guyane espagnole), Guyana (ex-Guyane britannique), Suriname (ex-Guyane néerlandaise), Guyane française et au Brésil les États Amapá et Roraima, et partie des États Pará et Amazonas (ex-Guyane portugaise)) Map of the Guiana Shield (source: d-maps.com/carte.php?num_car=284548) Caption: The red dotted line shows delimitation of the geological area. Amazonas and Bolivar States in Venezuela and the Departments of Vichada and Guainía in Colombia (ex-Spanish Guiana), Guiana (ex-British Guiana), Suriname (ex-Dutch Guiana), French Guiana and in Brazil the States of Amapá and Roraima, and parts of the States of Pará and Amazonas (ex-Portuguese Guiana)

**Figure 3:**
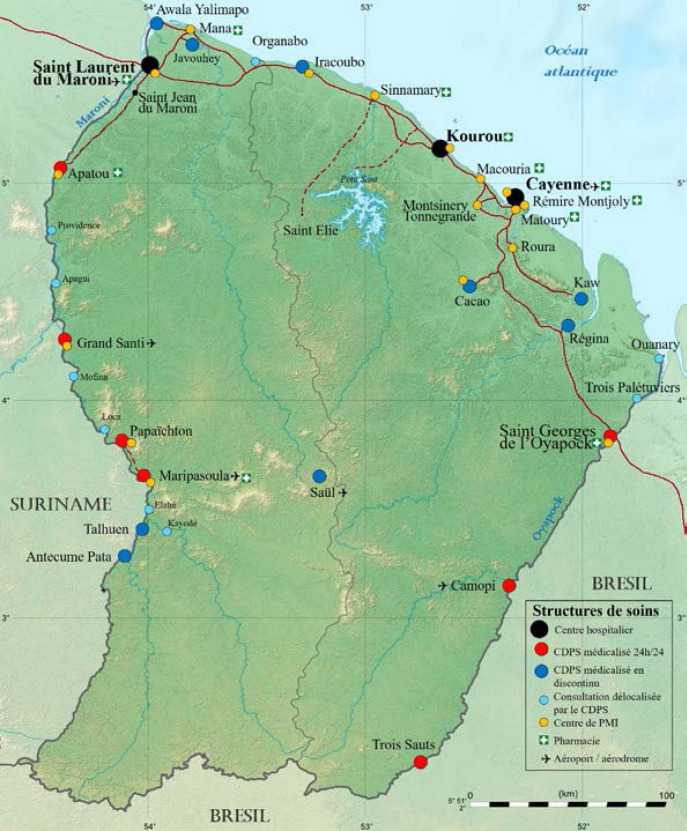
Carte géographique de répartition des structures de santé (Réalisation: É. Martin) Geographical distribution of healthcare structures (Creation: É. Martin)

**Figure 4:**
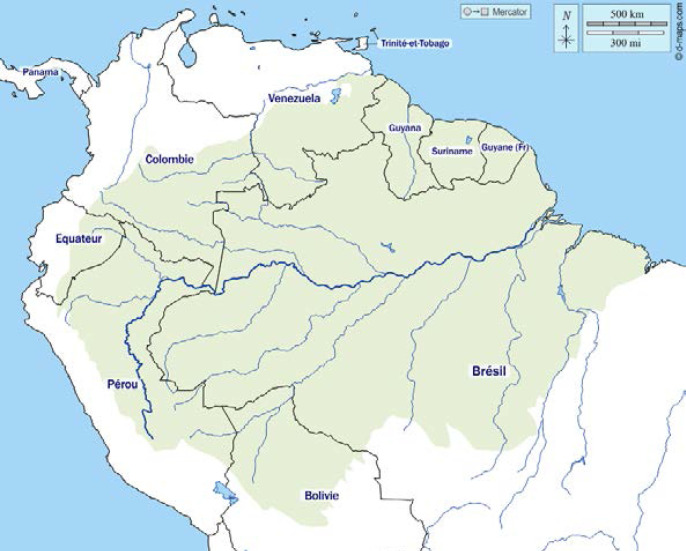
Carte politique de l'Amazonie (source: d-maps.com/carte.php?num_car=2316) Political map of Amazonia (source: d-maps.com/carte.php?num_car=2316)

La Guyane fait partie, avec la Guadeloupe, la Martinique et leurs dépendances, et Saint-Pierre-et-Miquelon, des territoires français d'Amérique. Il s'agit d'une collectivité territoriale, région monodépartementale. Située sur la côte nord-est de l'Amérique du Sud (Fig. 1), elle fait partie du plateau (ou bouclier) des Guyanes qui comprend d'est en ouest l’État de l'Amapá et une partie des États du Pará et de Roraima (ex-Guyane portugaise) au Brésil, la Guyane (Guyane française), le Suriname (ex-Guyane néerlandaise), le Guyana (ex-Guyane britannique), et les États de Bolivar et d'Amazonas au Venezuela (ex-Guyane espagnole) (Fig. 2). Elle est séparée du Suriname par le fleuve Maroni, et du Brésil par le fleuve Oyapock ainsi que par la chaîne montagneuse des Tumuc-Humac au sud (Fig. 3). Plus de 95% des 84 000 km^2^ de la Guyane (l’équivalent du Portugal) sont recouverts de forêt amazonienne. Les 10% restants sont les zones côtières. Le climat est de type équatorial avec quatre saisons: grande saison sèche de mi-juillet à minovembre, puis petite saison des pluies de mi-novembre à février; petite saison sèche en mars et enfin grande saison des pluies d'avril à mi-juillet. Les températures sont élevées toute l'année aux environs de 28-30 °C, sans grande différence entre le jour et la nuit, avec une température plus élevée en saison sèche, et une humidité moindre. Bien que ne représentant que 1,5% des 6,7 millions de km^2^ de l'Amazonie répartie sur 9 pays (Fig. 4), la Guyane partage avec ses voisins l'une des biodiversités les plus importantes au monde, avec un impact direct sur la variété des pathologies infectieuses et non infectieuses que l'on y rencontre.

### Peuples de Guyane

Loïc Epelboin, Marc-Alexandre Tareau

**Figure 5:**
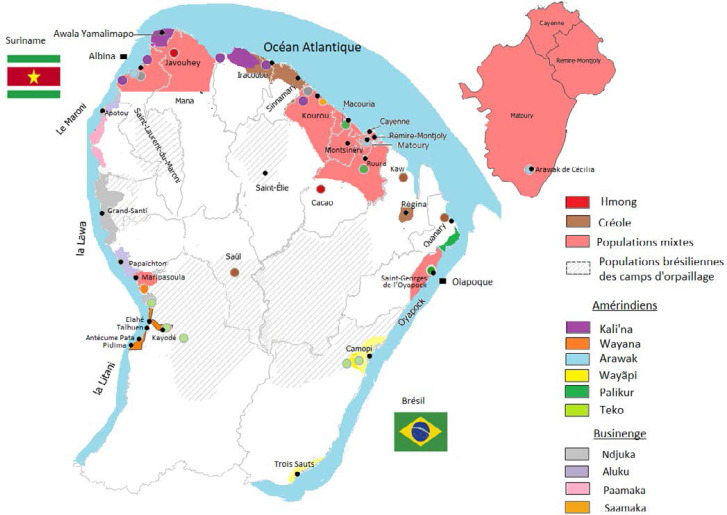
Carte de répartition des ethnies et langues de Guyane (réalisation: S. Rabier, L. Epelboin et M.-A. Tareau) Geographical distribution of ethnic groups and languages in French Guiana (creation: S. Rabier, L. Epelboin and M.-A. Tareau)

La population de Guyane est estimée à 281 678 habitants au 1^er^ janvier 2019 [178]. L’Île de Cayenne qui regroupe Cayenne, Rémire-Montjoly et Matoury, comprend à elle seule 50% de la population totale. Les autres grands centres urbains sont Saint-Laurent-du-Maroni, ville frontière de l'ouest du territoire en pleine expansion, et Kourou, où se situe le Centre spatial guyanais (CSG). La population est largement multiethnique avec une répartition communautaire variée sur le territoire. Les Amérindiens et les Businenges sont appelés peuples autochtones. Parmi les Amérindiens, on retrouve 6 groupes différents: les Wayãpi sur le haut Oyapock, les Wayana sur le haut Maroni, et les Teko sont présents sur les rives des deux fleuves (Fig. 5), tandis que les autres peuples amérindiens sont plutôt côtiers, parmi lesquels les Kaliña (anciennement appelés Galibi) à l'ouest, les Palikur à l'est et enfin les Arawak (ou Lokono), diversement répartis sur les communes du littoral. Les Businenges (prononcer Bouchinèngué; busi = forêt, nenge = nègre/homme), également appelés Noirs marrons ou Marrons, se retrouvent principalement sur le fleuve Maroni du même nom, bien que retrouvés également ailleurs en Guyane, et sont des descendants des esclaves africains échappés aux XVIII^e^ et XIX^e^ siècles des plantations de la Guyane néerlandaise. Parmi eux on retrouve les Aluku ou Boni à Maripasoula et Papaïchton, les Ndjuka à Grand Santi, ainsi que les Paamaka (également appelés Paramaka) et les Saamaka (également appelés Saramaka), plutôt sur le bas Maroni, Saint-Laurent-du-Maroni et le reste du littoral. Les Businenges, en croissance démographique rapide, représentent environ 1/3 de la population de Guyane. Les Créoles (Guyanais et Antillais) représentent environ 40% de la population, et les « Métropolitains » ou « Métros », originaires de la France hexagonale, représentent environ 12% de la population. Les termes « métropole » et « métropolitain » ne seront plus utilisés dans le reste de ce document, leur connotation post-coloniale faisant préférer le terme « Hexagone », plus neutre. Parmi les autres communautés présentes en Guyane on retrouve les Hmongs, réfugiés originaires du Laos et expatriés à la fin des années 1970 à la fin de la guerre du Vietnam, les Chinois, issus de différentes vagues migratoires dont certaines très anciennes, et enfin les populations issues de migrations de pays de la région Amérique et Caraïbe – Haïti, Brésil, Suriname, Guyana, Sainte-Lucie, République dominicaine et Pérou – qui sont parmi les nationalités les plus représentées [48, 207, 355]. Depuis quelques années, suite aux guerres au Moyen-Orient, une nouvelle communauté, constituée notamment de Syriens, mais aussi de Palestiniens, se rencontre de plus en plus dans les rues de Cayenne. Il s'agit pour eux d'une voie alternative à la traversée de la Méditerranée et ses dangers pour rejoindre la France. Chacune de ces populations, de par son lieu de vie, les écosystèmes qu'elle fréquente, ses origines géographiques et son mode de vie, présente des facteurs de risque spécifiques pouvant orienter le diagnostic médical.

### Système de santé guyanais

Loïc Epelboin, Pierre-Yves Carlier

En Guyane, le système public de santé repose sur les centres hospitaliers des trois principales villes du littoral (Centre hospitalier de Cayenne (CHC), Centre hospitalier de Kourou (CHK), Centre hospitalier de l'ouest guyanais (CHOG)) ainsi que les 17 centres délocalisés de prévention et de soins (CDPS) et les centres de protection maternelle et infantile (PMI) répartis sur tout le territoire (Fig. 3). Le secteur privé repose sur les médecins généralistes libéraux, quelques établissements à Cayenne ainsi que de rares médecins spécialistes libéraux. En matière d'effectifs, la Guyane comptait, au 1^er^ janvier 2022, 169 médecins libéraux et 36 ayant un exercice mixte, dont 1/4 a plus de 65 ans et 1/5 entre 60 et 64 ans. À la même date, les hôpitaux comptaient 411 praticiens au 1^er^ janvier 2022 (pour seulement 59 en 1999) [10, 96]. Les 30-49 ans représentent les 2/3 des médecins hospitaliers, avec 1/3 de femmes et 2/3 d'hommes. Chez les infirmiers, les effectifs augmentent plus vite en ville qu’à l'hôpital. Alors que la Guyane comptait 255 infirmiers libéraux en 2012, ils étaient 63% de plus (415) au 1^er^ janvier 2021. À l'hôpital, les effectifs sont passés de 975 à 1 369 sur la même période (+ 40%). Pouvant désormais compter sur la hausse des promotions de l'Institut de formation en soins infirmiers (IFSI) de Guyane, la profession connaît le même vieillissement que les médecins: en ville, 1 infirmier libéral sur 5 a plus de 60 ans. La maïeutique compte 200 sages-femmes: un peu plus de la moitié à l'hôpital (111), 40 en ville, 30 avec un exercice mixte et 19 salariées hors hôpitaux, alors qu'elles étaient entre 40 et 50 au début des années 2000. Près des 3/4 ont moins de 40 ans avec une extrême mobilité des jeunes diplômées et donc une difficulté à stabiliser les effectifs. Un tiers des sagesfemmes de Guyane exercent sur le territoire depuis moins de 2 ans, une sur cinq depuis 3 ou 4 ans et un quart depuis la période 2012-2018. Pour tenter de stabiliser les sages-femmes sur le territoire, une mission exploratoire est en cours pour la création d'une école en Guyane, à l'horizon de 3 à 4 ans. Les chirurgiens-dentistes étaient 85 au 1^er^ janvier 2022, les pharmaciens, 140 (ces 2 populations étant vieillissantes), les psychologues, 195, caractérisés par une population jeune, et enfin les techniciens de laboratoire, 117. Le Centre hospitalier régional universitaire (CH RU) de Guyane est attendu à l'horizon 2025 et devrait regrouper les 3 hôpitaux ainsi que les CDPS; trois des CDPS vont devenir des hôpitaux de proximité: celui de Saint-Georges-de-l'Oyapock, sur l'Oyapock à la frontière brésilienne, et ceux de Maripasoula et Grand Santi, sur le Maroni à la frontière surinamaise.

### Évacuations sanitaires (evasan)

Céline Michaud, Loïc Epelboin, Jean Pujo

Le terme evasan est l'acronyme qui désigne les évacuations sanitaires. Il englobe 2 types de situations très différentes.

La première est le transfert des patients des communes de l'intérieur, depuis les CDPS vers les hôpitaux du littoral, principalement le CHC, mais aussi le CHOG ou le CHK. Ces communes sont caractérisées par un isolement géographique important.

Quelques CDPS sont desservis par la route, et les patients peuvent donc être transférés par taxi collectif, véhicule personnel, ambulance, VSAB ou SMUR. À l'est, les patients pris en charge au CDPS de Saint-Georges-de-l'Oyapock peuvent être transférés par voie terrestre vers le CHC en 3 heures 30, avec passage du barrage de gendarmerie de Régina. À l'ouest, les CDPS de Javouhey, Awala-Yalimapo, Iracoubo et Apatou sont aussi accessibles par la route, avec la traversée du barrage de gendarmerie d'Iracoubo en cas de transfert vers le CHC ou le CHK. Pour les patients sans titre de séjour, une demande de laissez-passer auprès de la préfecture *via* l'ARS est indispensable pour passer ces deux barrages.

Pour les patients des CDPS vivant dans les autres communes des fleuves Oyapock et Maroni, il n'existe pas de réseau routier vers le littoral. Les transferts urgents sont réalisés par hélicoptère. Le SAMU a procédé, en moyenne sur ces 5 dernières années, à 782 transferts héliportés par an, sur déclenchement du médecin régulateur au CHC qui travaille en coordination avec les médecins des CDPS.

Pour les situations ne relevant pas de l'urgence, le transport aérien est disponible depuis Saül, Maripasoula, Grand Santi et Camopi (depuis 2021) vers Cayenne ou Saint-Laurent-du-Maroni. Les habitants du pays amérindien wayana sur le Haut Maroni (Talhuen, Antecume Pata) sont à 1 à 2 heures de pirogue de Maripasoula, et ceux de Papaïchton à 1 h 30 de piste. La voie aérienne, bien que subventionnée et/ou prise en charge partiellement ou complètement par la Sécurité sociale, reste coûteuse pour la population (70 à 90 euros pour un trajet aller simple) et ne dessert que les villes de taille moyenne. Les trajets jusqu’à l'aéroport de départ ne sont pas pris en charge. Là encore, pour les patients sans titre de séjour, une demande de laissez-passer auprès de la préfecture *via* l'ARS est indispensable pour prendre un vol intérieur.

Malgré l'existence de lignes aériennes, le fleuve reste la voie de communication privilégiée, en particulier de Grand Santi vers le CHOG et de Camopi vers le CHC *via* Saint-Georges, puis la route. La pirogue reste le mode de déplacement plébiscité du fait des centaines de zones d'habitat dénommées « kampoes » ou « écarts » qui parsèment les rives des fleuves conduisant ainsi à une mobilité aléatoire y compris pour les déplacements jusqu'au CDPS le plus proche ou un retour dans son village d'origine.

**Figure 6:**
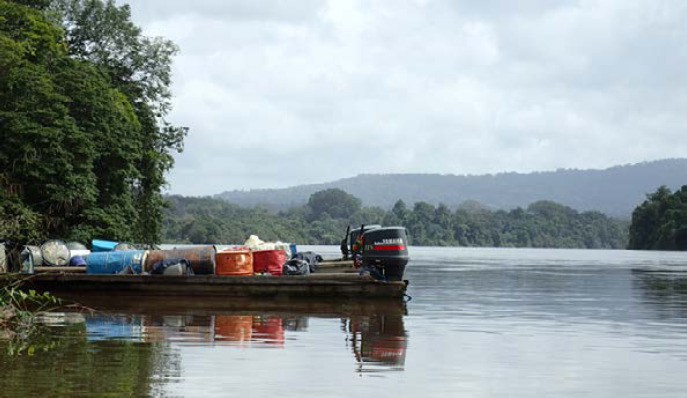
Pirogue surchargée de bidons d'essences remontant le Maroni vers Maripasoula (crédit photo: M. Douine) Pirogue overloaded with petrol cans going up the Maroni towards Maripasoula (photo credit: M. Douine)

La voie fluviale présente un coût élevé lié au prix de l'essence, à la complexité de la navigation marquée par la fréquence des rapides, parfois très dangereux à passer, surtout avec des pirogues surchargées, et la longueur des trajets, allant jusqu’à plusieurs jours pour rejoindre un village (Fig. 6).

La seconde situation est le transfert de certains patients, – faute de plateau technique suffisant ou de spécialité disponible en Guyane – vers les Antilles (80%), majoritairement vers le CHU de Martinique (notamment pour la cardiologie interventionnelle, la pédiatrie spécialisée, la neurochirurgie et la chirurgie thoracique), parfois la Guadeloupe (transplantation rénale), ou vers l'Hexagone (20%), en particulier Paris.

Ainsi, de par sa localisation géographique particulière en zone équatoriale, de l'immense diversité de la faune, de la flore et des écosystèmes, de sa diversité culturelle et de moyens diagnostiques et thérapeutiques de niveau européen, la Guyane présente un vaste panel de pathologies tropicales infectieuses et non infectieuses parfois peu connues, même dans la sous-région. Ces pathologies, auxquelles sont quotidiennement confrontés les cliniciens en Guyane, et pas seulement les infectiologues, doivent être évoquées ailleurs chez des patients ayant voyagé ou étant originaires de la Guyane et de façon plus générale de la grande région amazonienne.

## Pathologies Infectieuses Fébriles

### Zoonoses bactériennes

#### Fièvre Q

Loïc Epelboin

**Figure 7:**
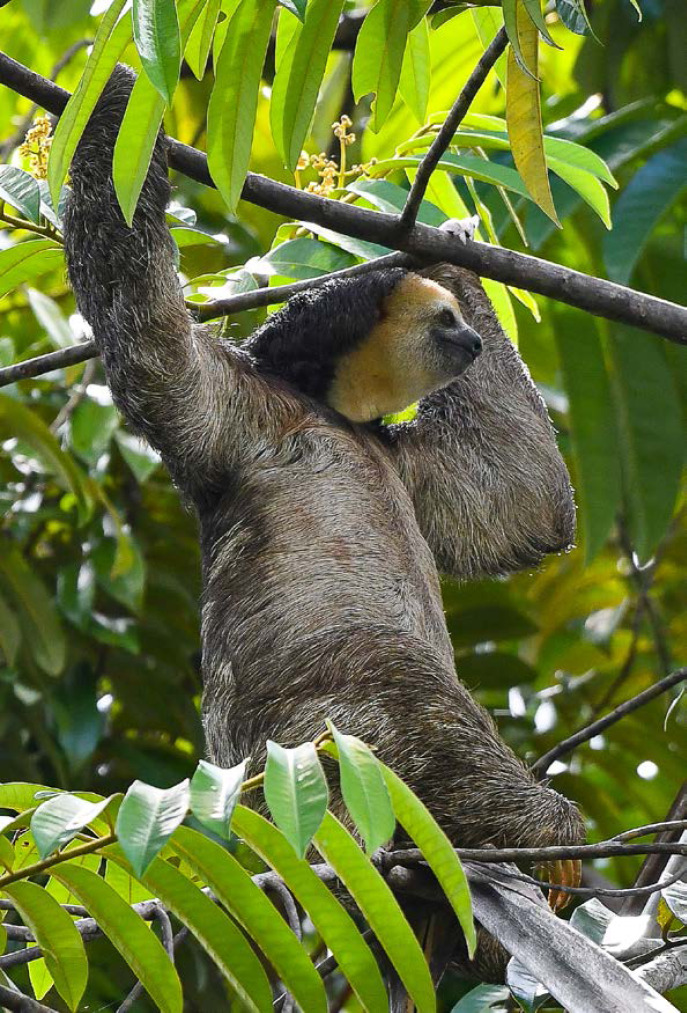
Paresseux à trois doigts (*Bradypus tridactylus*) (crédit photo: L. Epelboin) *Pale-throated sloth* (Bradypus tridactylus} (*photo credit: L. Epelboin*)

**Figure 8:**
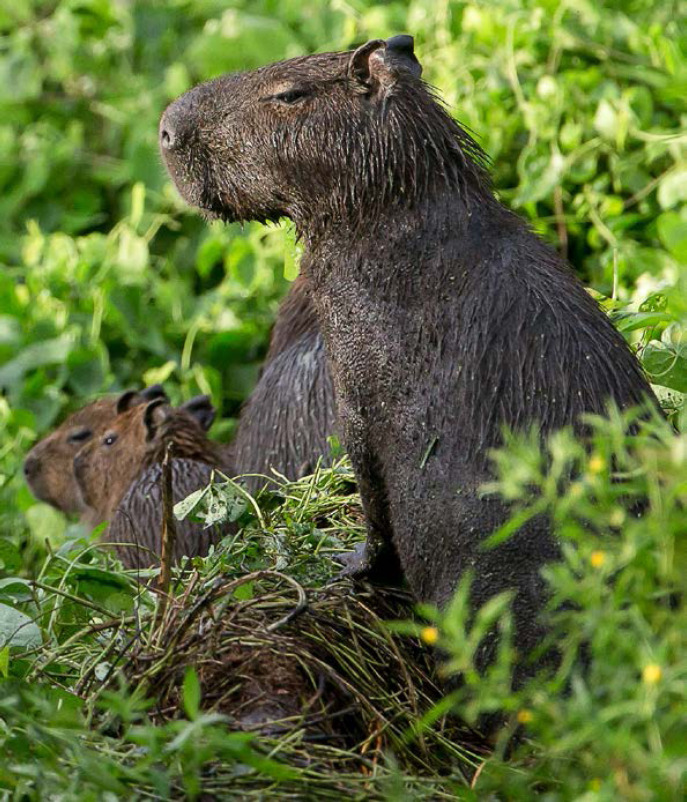
Cabiaï ou capybara *(Hydrochoerus hydrochaeris)* (crédit photo: N. Defaux) *Greater capybara* (Hydrochoerus hydrochaeris) *(photo credit: N. Defaux)*

**Figure 9:**
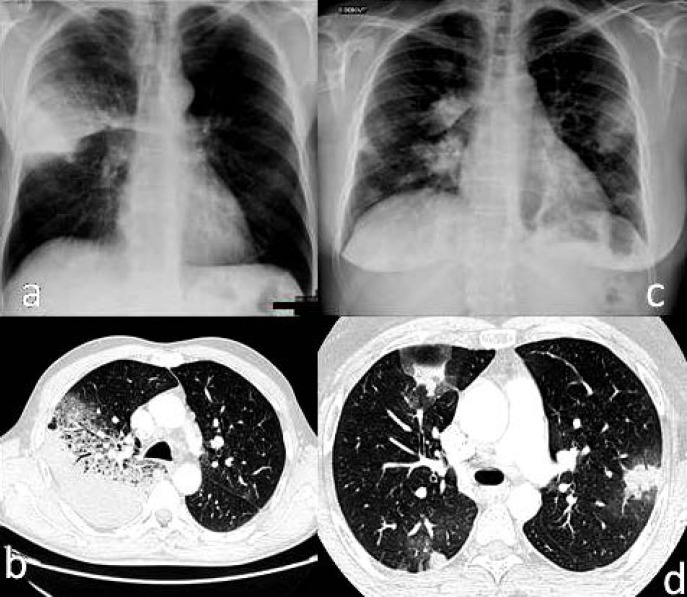
Radiographies et scanners thoraciques de 2 patients atteints de fièvre Q (crédit photo: M. Zappa) a. radiographie pulmonaire et b. scanner thoracique en fenêtre parenchymateuse: condensation alvéolaire unique systématisée du LSD; c. radiographie pulmonaire et d. scanner thoracique en fenêtre parenchymateuse: condensations alvéolaires de plus petite taille bilatérales touchant plusieurs lobes Chest radiographies and CT scans of 2 patients with Q fever (photo credit: M. Zappa) a. chest X-ray and b. chest CT in parenchymal window: single systematized alveolar condensation of LSD; c. chest X-ray and d. chest CT in parenchymal window: bilateral smaller alveolar condensations involving several lobes

La fièvre Q, zoonose bactérienne liée à *Coxiella burnetii* (*Cb*), présente une épidémiologie particulière en Guyane. Son incidence annuelle est très élevée, variant entre 25 et 40 cas pour 100 000 habitants contre 0,33 cas/100 000 habitants dans l'Hexagone. C'est ainsi le foyer hyperendémique le plus important du monde [361]. Cette incidence élevée ne semble retrouvée ni chez les voisins de la région amazonienne ni dans le reste de l'Amérique latine, faisant s'opposer deux hypothèses: celle d'une exception épidémiologique en Guyane, liée à un phénomène non expliqué, ou à l'opposé, celle de l'iceberg qui suggérerait que le phénomène guyanais n'est que le reflet d'une épidémie cachée sur le reste du continent [118, 120]. La forme pulmonaire représente plus de 90% des fièvres Q aiguës symptomatiques contre 8 à 37% dans l'Hexagone [110, 141]. Parallèlement, *Cb* est l'agent pathogène de 24 à 38% des pneumopathies hospitalisées à Cayenne contre 1% des pneumonies aiguës communautaires (PAC) hospitalisées au Royaume-Uni et en Europe et 2,3% en Amérique du Nord [118, 120]. Contrairement au reste du monde où les facteurs de risque d'acquisition de la maladie sont liés à la fréquentation du bétail et au milieu rural, les facteurs de risque identifiés à Cayenne sont moins clairs. Évoqués dans une première étude, le fait de travailler dans le BTP, d'observer des chauves-souris et des mammifères sauvages depuis chez soi, de vivre près de la forêt et réaliser des travaux de jardinage (débroussailleuse) étaient des facteurs de risque d'avoir une infection pulmonaire à *C. burnetii* [62, 141]. Les Hexagonaux et les Créoles sont plus fréquemment atteints que les autres communautés vivant en Guyane [118], les facteurs démographiques associés au fait d'avoir acquis la fièvre Q par rapport à la population générale sont d’être né en Europe, de vivre à Cayenne et ses environs, d’être de sexe masculin et d'avoir entre 30 et 60 ans [361]. Jusqu’à récemment, 30 ans de recherche n'avaient pas permis d'identifier les ovins et les bovins comme réservoir potentiel de la fièvre Q [299]. Une étude de séroprévalence chez le bétail a retrouvé un taux de prévalence apparente de 6,35% mais un taux d'exposition inter-élevage de 37,6% parmi 109 élevages, avec une prédominance chez les bovins [335]. Coté faune sauvage, quelques espèces ont été identifiées comme porteuses de la fièvre Q: paresseux à 3 doigts (Fig. 7) (*Bradypus tridactylus*), cabiaï ou *capybara* (Hydrochoerus hydrochaeris) (Fig. 8) et suidés sauvages (pécari à collier, *Pecari tajacu*, et pécari à lèvres blanches, *Tayassu pecari*) [62, 79, 116]. Du point de vue moléculaire, tous les cas identifiés par PCR en Guyane étaient dus à la même souche MST 17, isolée chez 8 patients ayant vécu ou voyagé en Guyane [213]. À l'inverse, MST 17 n'a été détectée dans aucune des 298 souches en provenance d'autres zones géographiques au CNR. Ce clone unique MST 17 semble plus virulent *in silico, in vitro* et *in vivo* comparé aux souches de référence Nine Mile et German strain [229]. Du fait de la fréquence de ce pathogène, les patients pris en charge pour une pneumopathie en Guyane sont traités en première intention par la doxycycline en sus du schéma antibiotique recommandé dans l'Hexagone. Les macrolides ne sont pas recommandés car la souche guyanaise est résistante à cette classe d'antibiotiques [111]. Une étude non publiée a montré que les patients traités par macrolides, comparés à ceux traités par doxycycline, évoluaient plus facilement vers la forme persistante focalisée [104]. La présentation clinique classique de la fièvre Q en Guyane est une pneumopathie aiguë communautaire avec opacité systématisée uni- ou multilobaire, peu différente de la pneumopathie à pneumocoque (Fig. 9). Elle est généralement accompagnée de céphalées, avec parfois une présentation pseudo-encéphalitique et/ou psychiatrique d’évolution rapidement favorable sous antibiotiques, et d'une élévation importante de la CRP (> 185 mg/l) associée paradoxalement à un taux de leucocytes normal, contrairement aux autres infections bactériennes à tropisme pulmonaire [81, 118]. C'est donc la première cause de pneumopathie aiguë communautaire de l'immunocompétent en Guyane.

#### Leptospirose

Paul Le Turnier, Loïc Epelboin

**Figure 10:**
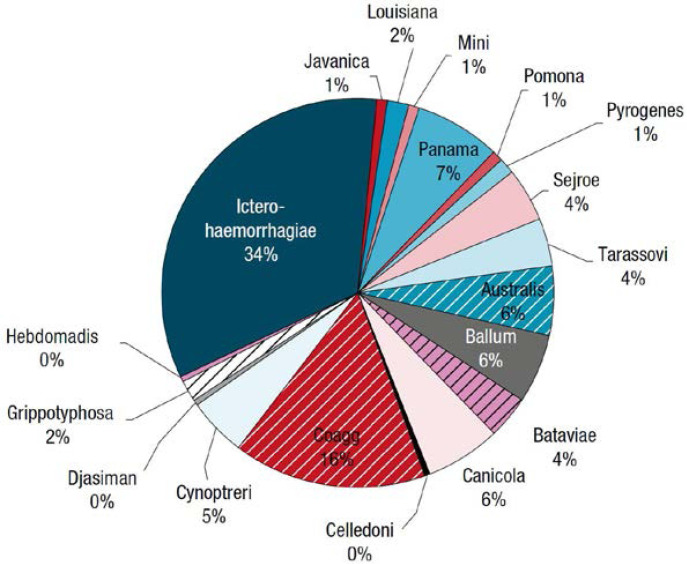
Répartition des sérogroupes et sérovars de *Leptospira* en Guyane, données du Centre national de référence de la leptospirose (Institut Pasteur, Paris), 1996-2015 *Distribution of* Leptospira *serogroups and serovars in French Guiana, data from National Reference Center for Leptospirosis (Institut Pasteur, Paris), 1996-2015*

La leptospirose est une zoonose fréquente dans la zone intertropicale. Classiquement, sa transmission repose sur la pénétration cutanée ou muqueuse de leptospires pathogènes à l'occasion d'un contact avec un milieu humide contaminé par de l'urine de rongeurs excréteurs. La présentation clinique associe un syndrome algique et fébrile aigu (céphalées, myalgies) avec des troubles digestifs (vomissements, diarrhées). Les signes plus spécifiques comme l'ictère ou, plus rarement, un syndrome hémorragique pulmonaire sont souvent retardés. Les anomalies biologiques sont une CRP élevée souvent supérieure à 100 mg/l, une hyperleucocytose à polynucléaires neutrophiles, une thrombopénie parfois profonde, une cytolyse hépatique et une élévation de la créatininémie et de la bilirubinémie totale qui sont de mauvais pronostic en cas d’élévation importante [40, 119]. Si le principal sérogroupe reste Icterohemorrhagiae en Guyane (˜ 30%), faisant poser la question de l'intérêt du vaccin pour les professions à risque (l'unique vaccin ne couvrant que cette souche), plus de 15 autres sérogroupes ont été décrits ces dernières années (Fig. 10). En Guyane, entre 2007 et 2014, 72 cas ont été identifiés sur l'ensemble du territoire, environ 40% présentaient une atteinte pulmonaire associée et 16% présentaient des critères de gravité, 12 patients ont été admis en réanimation et 4% sont décédés [119, 204]. Entre 2014 et 2021, 25 patients atteints de leptospirose étaient pris en charge en réanimation à Cayenne [Kallel, données non publiées], soit un doublement du nombre de cas. Entre janvier et juillet 2022, 47 patients ont été pris en charge à l'hôpital de Cayenne dont 6 en réanimation et un patient est décédé [Prince, données de surveillance hospitalière]. L'augmentation récente est probablement à relier à un indice pluviométrique exceptionnellement fort depuis 2020, responsable d'inondations répétées, notamment en zone urbaine, regroupant ainsi les conditions propices à la transmission de *Leptospira* [64]. Ces dernières années, une recherche plus systématique de cette étiologie pourrait aussi expliquer l'augmentation observée. Les phénomènes climatiques exceptionnels, les habitats insalubres, les dépôts sauvages de déchets et la présence de rongeurs excréteurs sont des facteurs de risque de leptospirose bien décrits et toujours très présents en Guyane. Ces conditions contribuent probablement au maintien d'une forte incidence sur notre territoire. L'identification des sérogroupes impliqués et le typage de souches restent malheureusement très limités en Guyane (Rapport CNR 2019 sur l'activité 2018). Depuis 10 ans, une seule souche a été identifiée: *Leptospira santarosai* sérogroupe *Sejroe* [189]. Des études à venir devraient permettre de mieux caractériser les souches, les réservoirs, les populations atteintes et les modes de transmission en Guyane. Ces éléments seront utiles pour cibler les interventions à mener afin de lutter contre certains facteurs de risque et informer les populations à risque.

### Mycobactéries respiratoires

#### Tuberculose

Mathilde Boutrou

La Guyane est la région de France où le taux de déclaration de tuberculose est le plus élevé, avec 27,5 cas pour 100 000 habitants en 2018 vs 7,1 au niveau national [156]. Son incidence semble être globalement stable depuis 10 ans du fait d'une transmission endémique de la maladie, à laquelle s'associent de nouveaux cas provenant des pays limitrophes et d'immigration à fort taux de prévalence [350]. Ainsi entre 50 et 100 cas sont rapportés chaque année. Entre 2007 et 2017, 405 patients ont été hospitalisés pour tuberculose à l'hôpital de Cayenne, parmi lesquels 63% étaient des hommes, d’âge médian 40 ans (intervalle interquartile: 29-50 ans) [43]. Parmi eux, 74% étaient nés à l’étranger, avec un délai médian d'arrivée en Guyane de 5 ans. Dans cette étude, 1/4 des patients étaient infectés par le VIH. Les personnes originaires d'Haïti constituaient la population la plus représentée (26%) alors qu'elles ne représentent que 10% de la population guyanaise. La localisation était pulmonaire dans 80% des cas, extra-pulmonaire dans 25%, la localisation ganglionnaire arrivant en première position. La prise en charge de la maladie ne présente pas de particularités, si ce n'est la difficulté du diagnostic différentiel avec l'histoplasmose disséminée chez les PVVIH. Chez ces patients, la co-infection tuberculose-histoplasmose n'est pas rare et pose des problèmes d'interactions médicamenteuses non négligeables [300]. Le phénomène de multirésistance reste relativement limité en Guyane: 2,0% des 824 souches guyanaises étudiées entre 1995 et 2011 au CNR des mycobactéries de Guadeloupe avaient une résistance associée à la rifampicine et l'isoniazide (souches MDR), bien que 13,1% d'entre elles étaient résistantes à au moins l'un des antituberculeux majeurs, le plus souvent l'isoniazide [237]. Sur la cohorte cayennaise 2007-2017, seuls 4% des souches avaient une résistance à l'isoniazide ou à la rifampicine [43]. La majorité des cas est diagnostiquée suite à un recours spontané aux soins et la proportion d'issues de traitement favorables (72%) n'a pas augmenté par rapport à la période 2007-2010 [350]. Le nombre de cas pourrait être plus important si le dépistage systématique de la tuberculose était proposé aux personnes migrantes primo-arrivantes originaires des pays de forte endémie – incidence > 40/100 000 (Guyana, Brésil, République dominicaine notamment) ou de très forte incidence –> 100/100 000 (Haïti et pays d'Afrique subsaharienne en particulier).

#### Mycobactéries atypiques

Loïc Epelboin

L’épidémiologie des infections à mycobactéries non tuberculeuses, dites atypiques, d'expression respiratoire (MBNTER) diffère en Guyane de celle de l'Hexagone. Ainsi, une étude portant sur les infections à MBNTER entre 2008 et 2018 a identifié 178 patients dont 147 étaient porteurs asymptomatiques et 31 présentaient des symptômes compatibles avec une infection active [58].

Parmi ces 178 patients, on retrouvait une majorité d'hommes (61%), jeunes (âge médian 49 ans), en situation de précarité (64%), avec des comorbidités respiratoires (33%) ou une immunodépression (46% de patients séropositifs pour le VIH dont 39% avec des CD4 ≤ 50/mm^3^). *Mycobacterium avium intracellulare* (MAC) suivi de *M. fortuitum*, puis de *M. abscessus* étaient les espèces les plus fréquemment retrouvées (38%, 19% et 6% respectivement). *M. avium intracellulare* et *M. abscessus* représentaient respectivement 81% et 16% des maladies pulmonaires à MBNT. La mortalité à un an, toutes causes confondues de MBNTER, était de 29% chez les malades ayant une infection confirmée, ce qui est très élevé par rapport à l'Hexagone.

### Infections virales chroniques

#### VIH

Nicolas Vignier, Loïc Epelboin

**Figure 11:**
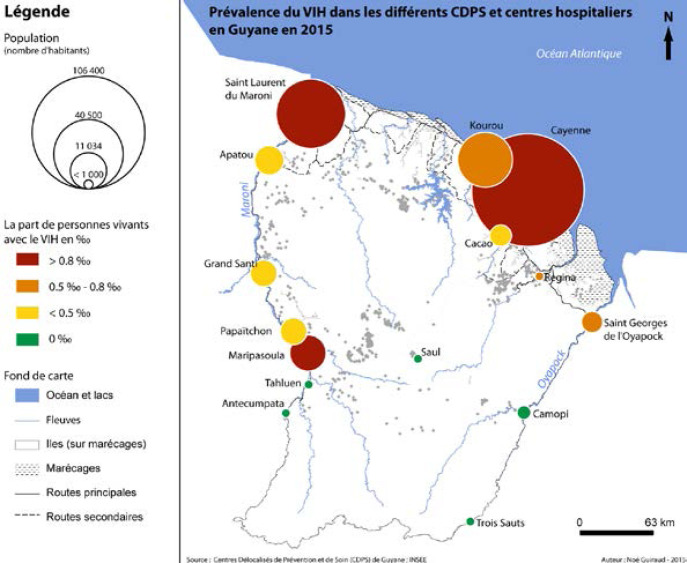
Prévalence du VIH dans les différents CDPS et CH en Guyane en 2015 [242] HIV prevalence in the various CDPS (Off-site prevention and healthcare centres) and CH (General hospitals) in French Guiana in 2015 [242]

La Guyane est le département ayant la prévalence d'infection par le VIH la plus élevée de France, avec une prévalence supérieure à 1% [262]. On estime ainsi que plus de 4000 personnes vivent avec le VIH (PVVIH) en Guyane en 2022, dont 10% ne sont pas diagnostiquées, 1937 sont suivies à l'hôpital, 497 en suivi exclusif en ville et 1421 sont perdues de vue depuis plus d'un an [107]. En 2021, 76 personnes ont découvert leur séropositivité (118 en 2019), principalement des hommes (53%), originaires d'Haïti (63%). Le mode de transmission est essentiellement hétérosexuel (81%) avec autant de femmes que d'hommes infectés, notamment du fait de la fréquence des rapports sexuels transactionnels et du multipartenariat. Parmi les personnes migrantes vivant avec le VIH, on estime que plus d'une infection sur deux a été acquise sur le territoire guyanais et non dans le pays d'origine et que le diagnostic survenait en médiane 4,5 ans après l'infection [258]. L’épidémiologie de l'infection est sensiblement différente selon que l'on se trouve sur l’Île de Cayenne, dans l'ouest ou bien dans les communes isolées le long des fleuves Maroni et Oyapock (Fig. 11). Ainsi, à Cayenne, les PVVIH originaires d'Haïti (51%), du Guyana (10%) et de l'Hexagone (9%) sont majoritaires, là où ceux originaires du Suriname (57%) et d'Haïti (13%) prédominent à Saint-Laurentdu-Maroni, tandis que sur les fleuves, les PVVIH originaires du Brésil sont plus nombreuses, en lien avec l'orpaillage illégal [244]. En Guyane, 24 à 35% des patients sont dépistés à un stade tardif (CD4 < 200/mm^3^), les personnes migrantes étant les plus concernées [261]. Les co-infections avec les hépatites virales sont rares (4% de co-infection VHB, 5% de co-infection HTLV-1) et les ruptures de suivi sont fréquentes [245]. Les 5 pathologies opportunistes classant sida les plus fréquentes sont: l'histoplasmose disséminée, la tuberculose, la toxoplasmose cérébrale, la candidose œsophagienne et la pneumocystose pulmonaire [69]. La place prépondérante de l'histoplasmose disséminée au cours de l'infection par le VIH sera détaillée un peu plus loin et celle de la tuberculose l'a été juste avant. En 2021, 14 PVVIH sont décédées, à un âge médian de 57 ans [69]. Des équipes spécialisées prennent en charge les patients dans les 3 hôpitaux des 3 principales villes du littoral et une équipe mobile dédiée à l'infectiologie réalise des consultations dans les principaux CDPS tous les 1 à 3 mois. Les traitements antirétroviraux sont les mêmes que ceux disponibles dans l'Hexagone. Parmi les patients traités, 90% sont en succès thérapeutique; l'enjeu étant ainsi le retour dans le soin des personnes perdues de vue et le diagnostic précoce des personnes dont le statut est inconnu. De plus, après quelques échecs de la prévention il y a quelques années, 92 enfants sont nés en 2021 de mères vivant avec le VIH sans être infectés. Si les difficultés de prise en charge des PVVIH que rencontrent les praticiens en Guyane sont communes à d'autres territoires, avec en premier lieu la grande précarité et ses conséquences sur le suivi de la maladie, certains obstacles sont spécifiques à la Guyane. Ainsi, la prise en charge des patients vivant de part et d'autre des deux fleuves frontières, Oyapock et Maroni, représente un véritable challenge du fait de l'inégalité des soins proposés entre la Guyane d'un côté et le Brésil et le Suriname de l'autre, notamment en matière d'accès aux ARV, mais aussi aux soins et aux diagnostics d'infections opportunistes et de suivi clinique et biologique de qualité [183, 247]. Des initiatives locales se sont mises en place pour favoriser la prise en charge conjointe transfrontalière. À l'est, un projet de coopération de professionnels de santé de Saint-Georges-de-l'Oyapock et de la ville frontière brésilienne d'Oiapoque a été mis en place avec succès (www.idsante.eu/nos-projets/oyapock-cooperation-sante/) [39]. Enfin, l'accompagnement par des médiateurs en santé, bien implanté maintenant dans les CDPS et en voie de mise en place dans les hôpitaux du littoral, permet de favoriser le bon suivi de ces patients aux origines culturelles multiples et à la situation sociale complexe.

#### HTLV

Karim Abdelmoumen

Le virus HTLV-1 (Human T-cell Leukemia virus type 1) est le premier rétrovirus oncogène découvert chez l’être humain et touche 5 à 10 millions d'individus dans le monde répartis en foyers d'endémie dont la Guyane fait partie [8]. Dans 2 à 7% des cas, ce rétrovirus est responsable de diverses pathologies dont les deux plus sévères sont la leucémie/lymphome à cellules T de l'adulte (ATL) et la paraparésie spastique tropicale ou myélopathie associée à HTLV-1 (TSP/HAM). L'infection à HTLV-1 et les pathologies associées sont reconnues comme enjeu de santé publique par l'OMS qui a publié un rapport en mars 2021. Cette infection reste néanmoins méconnue des professionnels de santé, particulièrement en Europe [67]. En Guyane, c'est principalement la communauté businenge du fleuve Maroni qui est la plus exposée à l'infection à HTLV-1, suivie des communautés créoles guyanaises, haïtiennes et amérindiennes avec des prévalences variables selon les ethnies [192, 365]. Ainsi, HTLV-1 infecte jusqu’à 40% des personnes dans certains villages businenges, majoritairement des femmes. En comparaison, la séroprévalence de HTLV-1 est de 3% dans toute la Guyane et de 0,03% dans l'Hexagone. De même, l'incidence de l'hémopathie maligne associée à HTLV-1, l'ATL, est plus importante en Guyane (2,03 pour 1000 habitants, par an) que celle du cancer du sein ou du cancer de l'endomètre [306]. L'ATL en Guyane se présente principalement sous la forme « aiguë » de la classification de Shimoyama, avec un âge médian de 54 ans au diagnostic, un tropisme cutané important (39% des cas) et un pronostic extrêmement sombre: 11% de survie à 4 ans [1]. L'hypercalcémie est souvent présente au diagnostic dans les formes agressives (leucémies aiguës et lymphomes). Il faut toujours rechercher et traiter l'anguillulose en cas de séropositivité au HTLV-1. En effet, le portage d'anguillules augmente le risque de développer une maladie sévère liée à HTLV-1 (ATL et TSP/HAM) et la séropositivité HTLV-1 augmente le risque de développer une anguillulose maligne. Bien que quelques cas aient été décrits chez des Créoles guyanais, la TSP/HAM reste peu fréquente en Guyane alors qu'elle est bien décrite aux Antilles et au Brésil [272]. La dernière cohorte prospective de femmes séropositives à HTLV-1, avec 16 ans de suivi, n'a mis en évidence aucun cas de TSP/HAM [306].

Le tropisme de HTLV-1 concerne principalement les lymphocytes T CD4+. Le virus peut ainsi se transmettre par contact sexuel (de l'homme vers la femme surtout), par voie sanguine mais avant tout par l'allaitement prolongé. La transmission serait inexistante durant la grossesse. Chez les enfants allaités de mères vivant avec HTLV-1, le taux de transmission est de 10 à 25%. Une durée d'allaitement maternel de plus de 6 mois ainsi qu'une charge provirale HTLV-1 élevée chez la femme allaitante sont les 2 facteurs de risque principaux de transmission mère-enfant. S'infecter à HTLV-1 par l'allaitement est par ailleurs un facteur de risque reconnu de développer plus tard un ATL, comparativement aux autres modes de transmission (sexuelle et sanguine). Le Japon a montré une nette réduction de la prévalence de l'infection en contre-indiquant l'allaitement maternel aux mères porteuses de HTLV-1, passant de 20% à 2,5% [182, 302]. Dans l'ouest de la Guyane, la séroprévalence HTLV-1 de l'ensemble des accouchées est de 4,4% avec un taux plus élevé (5,7%) chez les Businenges [274]. En Guyane, la sérologie HTLV-1 devrait être aussi facilement proposée que la sérologie VIH et ce, quelle que soit l'origine ethnique, avec la difficulté cependant de devoir annoncer une maladie le plus souvent bénigne et exceptionnellement grave et incurable.

#### Hépatite virale B

Nicolas Vignier

La Guyane est aussi un territoire où les infections chroniques par le virus de l'hépatite B (VHB) sont fréquentes. En effet, contrairement à l'Hexagone et à la moyenne du continent américain (0,5%) [376] mais à l'instar des pays d'origine d'une partie de sa population, la Guyane est un territoire de moyenne endémie selon la définition de l'OMS (prévalence de l'antigène HBs > 2%) [138]. Dans un premier travail réalisé chez les femmes enceintes en 2007, la prévalence variait selon les communautés d'appartenance de 0% (caucasiennes) à 11% (femmes hmongs et d'origine asiatique), en passant par 2,1% (businenges) et 2,5% (haïtiennes) [214]. La prévalence a été évaluée à 2,1% en 2012-2016 dans les communes isolées de Guyane, à 4,1% chez les détenus en 2014 et à 4,6% chez les orpailleurs illégaux en 2015 [102, 175]. L'analyse des données de dépistages réalisés dans les trois centres de prévention santé de la Croix-Rouge française entre 2007 et 2018 retrouvait une prévalence de 4% chez les hommes et 2% chez les femmes. Cette prévalence était de 5,5% chez les personnes originaires du Suriname et de 5% chez celles originaires d'Haïti en 2019, contre 0,8% et 0,7% pour les consultants nés en France et au Brésil respectivement [221]. Une baisse de la prévalence était cependant observée chez les moins de 25 ans depuis 2010, en lien probable avec la vaccination généralisée dans les pays d'origine [221]. Cependant, chez les plus âgés, le taux de personnes non immunes et donc susceptibles restait à un niveau élevé (40% en 2010) et augmentait dans les années récentes (60% en 2018), malgré l'existence d'une vaccination efficace et théoriquement accessible. Bien que la prévalence soit élevée, peu de personnes concernées sont dépistées et accèdent à une prise en charge spécialisée. Sur la base des données du logiciel Nadis (qui n'est pas utilisé par tous les médecins suivant des patients avec hépatite virale B), seuls 293 patients étaient en cours de suivi sur les trois hôpitaux de Guyane en 2021, 725 étaient perdus de vue, là où on peut estimer que près de 6 000 infections chroniques relèveraient d'un suivi; 61% d'entre eux sont des hommes, âgés en médiane de 39 ans et majoritairement (56%) nés en Haïti et arrivés depuis moins de 3 ans [69]. Parmi eux, 85 (29%) sont sous traitement antiviral. Un travail récent de recherche active sur la cohorte hospitalière de Cayenne a permis de réintégrer dans le soin 17% des patients perdus de vue et a révélé que le manque de compréhension de l'indication et de l'importance d'un suivi était la principale raison de l'arrêt du suivi. Plus inquiétant encore, 31% des personnes hospitalisées dans les hôpitaux de Guyane pour un cancer du foie, 30% pour une cirrhose décompensée et 28% pour une insuffisance hépatique aiguë n'avaient pas été dépistées pour le VHB et le VHC [374]. Parmi les 89 cancers du foie pris en charge dans les hôpitaux de Guyane entre 2009 et 2020, 22 étaient liés à une infection VHB (25%), 8 VHC et 3 VHD. L'hépatite B représentait ainsi la première cause de cancer du foie en Guyane, suivie par la consommation excessive d'alcool.

#### Hépatite virale C

Nicolas Vignier

L'infection par le virus de l'hépatite C est beaucoup plus rare que celle par le VHB (0,7%) [138]. Sa prévalence est estimée à 0,5% dans la région Amérique de l'OMS en 2019. Elle a été estimée à 0,7% chez les orpailleurs illégaux en Guyane [102]. La grande majorité des patients infectés par ce virus ont été traités avec succès ces dernières années suite à l'apparition des nouveaux antiviraux à action directe.

#### Papillomavirus

Maylis Douine

En Guyane, le cancer du col de l'utérus est le deuxième cancer le plus fréquent chez la femme alors qu'il se situe en 12^e^ position dans l'Hexagone [177]. Les données du Registre des cancers de Guyane ont montré que l'incidence du cancer du col en 2003-2008 était 4 fois plus élevée que dans l'Hexagone et la mortalité 5 fois plus élevée, notamment en raison d'un dépistage tardif, en particulier chez les femmes vivant dans l'intérieur de la Guyane [101, 322]. La nécessité d’être transférée dans l'Hexagone pour la radiothérapie (absente en Guyane) a pu jouer un rôle sur l'acceptabilité du traitement et donc la mortalité. L'incidence semble diminuer depuis 2010. Un âge précoce du premier rapport sexuel et le multipartenariat ont été évoqués comme facteurs de risque, sans que cela ait fait l'objet d'une confirmation épidémiologique [6].

En 2013, une étude auprès de plus de 600 femmes vivant sur les fleuves frontières a retrouvé une prévalence du portage de HPV de 35% tous âges confondus, suivant une classique forme en U selon l’âge [5]. Parmi les 61 femmes avec des lésions cytologiques au frottis, 52 (85%) avaient un test positif pour HPV [6]. Les HPV circulants étaient différents de ceux de l'Hexagone avec une prédominance de 52, 16, 68, 31, 53, 58, 18 et 56 parmi les HPV à haut risque [5]. Ces résultats ont entraîné la mise en place d'un dépistage organisé dès l’âge de 20 ans en Guyane depuis 2012 en lien avec le Centre régional de coordination des dépistages du cancer. Une thèse de médecine réalisée en 2021 montrant une faible prévalence des cancers et lésions de haut-grade chez les femmes de moins de 25 ans incite à revoir l’âge de dépistage: systématique à partir de 25 ans, ciblé à moins de 25 ans (entrée très précoce dans la vie sexuelle, grande multiparité, antécédent d'IST à répétition, femmes vivant avec le VIH) [17]. La vaccination par un vaccin nonavalent (Gardasil9^©^) est également fortement recommandée chez tout jeune, fille et garçon, de plus de 11 ans. Il reste primordial de prêter une attention particulière au suivi gynécologique des patientes de Guyane, en particulier pour le dépistage du cancer du col, notamment chez les femmes précaires ou vivant dans les zones isolées.

### Arboviroses

Timothée Bonifay, Dominique Rousset, Loïc Epelboin

#### Dengue

**Figure 12:**
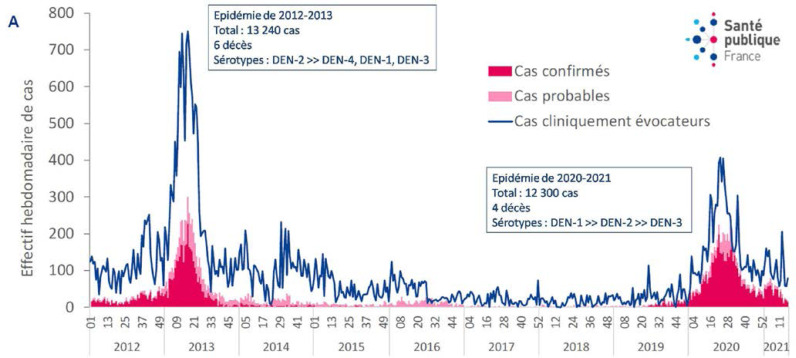
Nombre hebdomadaire estimé de cas cliniquement évocateurs de dengue chez des patients ayant consulté en médecine de ville ou dans un centre de santé et nombre de cas confirmés de dengue, Guyane, janvier 2012 à avril 2021 (source: Luisiane Carvalho, Santé publique France) Estimated weekly number of clinically suggestive cases of dengue in patients having consulted a practitioner in town or a health centre, and number of confirmed dengue cases, French Guiana, January 2012 to April 2022 (source: Luisiane Carvalho, Santé publique France)

La dengue est une arbovirose de la famille des Flaviviridae, importée aux Amériques depuis l'Ancien Monde à partir du XVII^e^ siècle *via* le commerce triangulaire. Depuis le début des années 2000, des vagues épidémiques régulières de dengue se sont succédées tous les 3 à 5 ans avec une co-circulation de différents sérotypes, même si cette périodicité a été un peu élargie à l'occasion des émergences successives des virus chikungunya puis Zika (Tableau I). Une alternance des sérotypes dominants est observée d'une épidémie à l'autre, associée à des phénomènes d'extinction puis de réintroduction de nouveaux génotypes à partir des virus circulant dans la région. Les cas notifiés en interépidémie, diagnostiqués par sérologie (IgM), sont étiquetés comme probables, mais peuvent traduire des infections passées à travers des réactivations immunologiques, même s'il peut aussi s'agir de cas importés ou de petits clusters. *Aedes aegypti* est le principal vecteur identifié, *Aedes albopictus* n’étant pas présent sur le territoire [35]. Au cours des dernières années, on a assisté à des épidémies de plus en plus fréquentes (2006, 2009-2010, 2013, 2020-2021) avec un nombre de cas cliniquement évocateurs variant de 10 000 à 18 000 selon les épidémies. La dernière épidémie a duré de janvier 2020 à juin 2021 (majoritairement DENV-1) et était concomitante de la pandémie de SARS-CoV-2. Le nombre de cas évocateurs a été estimé à 10 891 (contre 13 240 en 2013), pour une incidence de 38/1000 habitants, avec 282 patients hospitalisés (contre 701 en 2013) et 3 décès enregistrés dont 2 indirectement liés à la dengue (Fig. 12). En 2021, une étude a évalué la séroprévalence, tous sérotypes confondus, à 73,1% en population générale guyanaise [19]. Sur un territoire où sévissent plusieurs pathologies à présentations clinico-biologiques proches, telles que les infections par les virus de la dengue, du chikungunya et du Zika, le paludisme, la primo-infection VIH, la fièvre Q ou la leptospirose, un bilan biologique est pratiquement toujours nécessaire pour éliminer les pathologies parasitaires et bactériennes justifiant un traitement spécifique, et ce même au cours des épidémies. Des études ont récemment montré qu'une CRP > 50 mg/l était très évocatrice d'un diagnostic autre que la dengue, comme la leptospirose ou le paludisme [117, 202]. Aucun programme de vaccination contre la dengue n'a pour l'heure été mis en place en Guyane, notamment du fait des précautions nécessaires pour ce vaccin (nécessité d'une immunisation préalable), mais d'autres vaccins arrivent actuellement sur le marché et pourraient mettre le sujet de nouveau à l'ordre du jour [287].

**Tableau I:** Récapitulatif des données de séroprévalence et d'incidence des 7 principales arboviroses d'intérêt médical en Guyane Summary of seroprevalence and incidence data for the 7 major arboviroses of medical interest in French Guiana

Arbovirus	Acronyme	Famille (Genre)	Principaux vecteurs	Séroprévalence	Données épidémiologiques
Fièvre jaune	YFV	Flaviviridae (*Flavivirus*)	*Ae. aegypti* (cycle urbain) *Haemagogus* spp. et *Sabethes* spp. (cycle selvatique)	95,0%*	1 décès en 1998 2 décès en 2017-2018 2 décès en 2020
Dengue	DENV	Flaviviridae (*Flavivirus*)	*Ae. aegypti*	73,1%**	2005 DENV3 majoritaire / 2006 DENV2 majoritaire 13 700-16 200 cas 2009-2010 DENV-1 (67,6%) > DENV-4 (27%) (2009 DENV1 majoritaire; 2010 Cocirculation DEN1 et DEN4) ˜7 800 cas 2012-2013 (DENV2) ˜16 000 cas 2020-2021 (DENV1) ˜10 000 cas
Zika	ZIKV	Flaviviridae (*Flavivirus*)	*Ae. aegypti*	23,3%**	2015-2017 ˜9 700 cas
Chikungunya	CHIKV	Togaviridae (*Alphavirus*)	*Ae. aegypti*	20,3%**	2014-2015 ˜16 000 cas
Mayaro	MAYV	Togaviridae (*Alphavirus*)	*Haemagogus spp. Ae. aegypti*	3,3%**	17 cas de 2003 à 2019 ˜15 cas en 2020
Tonate	TONV	Togaviridae (*Alphavirus*)	*Culex portesi*	11,9%***	45 cas de 2003 à 2016
Oropouche	OROV	Bunyaviridae	*Culicoides paraensis Culex quinquefasciatus?*	ND	41 à 58 cas en août-septembre 2020 à Saül

* [129]

** [19]

*** [354]

#### Chikungunya

Le chikungunya est une arbovirose ayant la particularité d'associer un syndrome dengue-like à des arthralgies parfois intenses. Le continent américain a connu entre 2014 et 2015 une épidémie sans précédent d'infection par le virus du chikungunya (CHIKV), un arbovirus de la famille des Togaviridae et du genre *Alphavirus* transmis par *Aedes aegypti* voire *Aedes albopictus* pour le lignage ECSA-Océan indien. Après les premiers cas autochtones d'infection à CHIKV rapportés à Saint-Martin (Antilles françaises) fin 2013, les premiers cas autochtones du continent sud-américain ont été rapportés en Guyane en février 2014. L’épidémie en Guyane n'a pas été explosive comme aux Antilles. Un nombre total de cas estimés à environ 16 000 a été enregistré. En 2021, la séroprévalence en population générale a été estimée à 20,3% [19]. *Ae. aegypti* a été le principal vecteur de l’épidémie américaine de 2013-2014 avec la souche asiatique, et une deuxième souche, ECSA, a circulé de façon concomitante au Brésil. Si la symptomatologie rapportée en Guyane était relativement superposable aux formes décrites à La Réunion, avec un tableau regroupant fièvre et arthralgies distales au premier plan, des cas atypiques et ou sévères ont été rapportés: formes neurologiques, encéphalite ou syndrome de Guillain-Barré, chocs septiques dus au CHIKV ou encore purpura thrombocytopénique thrombotique [35, 36]. Si le CHIKV ne semble plus circuler sur le territoire guyanais et aux Antilles où il n'a plus été détecté depuis 2016 en dehors de rares cas importés, le risque à moyen terme d'une nouvelle épidémie est réel. Le Brésil fait d'ailleurs face à de nouvelles épidémies depuis 2020, avec plusieurs dizaines de milliers de cas enregistrés. Ainsi devant une fièvre avec arthralgies au retour de Guyane, et plus largement d'Amérique latine, une infection par CHIKV doit être recherchée par PCR avant J7 du début des symptômes et/ou par sérologie IgM à partir de J5. En outre, un virus proche du CHIKV, le virus Mayaro (cf. infra), a une présentation clinique de type arthralgies fébriles très proche et est endémique en Guyane. Il doit également être évoqué en cas d'atteinte « Chik-like ». Le risque d'exportation de ces arboviroses à destination de l'Hexagone et de la survenue de cas autochtones est réel dans un contexte d'expansion du vecteur *A. albopictus* à plus de la moitié des départements de France.

#### Zika

Succédant aux épidémies liées au DENV en 2013 et au CHIKV en 2014-2015, une épidémie due au virus émergent Zika (ZIKV) a sévi en Guyane et en Amérique du Sud en 2015-2016. L’épidémie qui a débuté dans le Pacifique en 2013 s'est étendue au Brésil puis à tout le continent américain en 2015. En Guyane, 23% de la population a été infectée parmi laquelle 26% seulement a eu une forme symptomatique [130]. L'infection par ZIKV semblait complètement bénigne jusqu'en 2014, puis des formes plus préoccupantes ont été décrites, d'abord en Polynésie française puis au Brésil [271]. Parmi elles, une atteinte neurologique de l'adulte avec une sur-incidence de syndromes de Guillain-Barré et une atteinte fœtale et congénitale illustrée par des malformations neurologiques sévères dont des microcéphalies chez des fœtus ou nouveau-nés dont la mère aurait fait une infection au cours de la grossesse [251]. Si les premières données étaient extrêmement alarmantes, il semblerait que le poids de ZIKV soit plus faible qu'initialement décrit, avec des taux de complications qui semblent similaires à ceux d'autres infections congénitales [298]. L’équipe du CHOG a partagé de manière dynamique son expérience sous forme de publications scientifiques sur l'impact materno-infantile de l'infection par ZIKV [164, 297]. Lors de l’épidémie, il était recommandé de réaliser au cours de la grossesse un dépistage sérologique systématique répété et un suivi échographique renforcé. En cas d'infection confirmée, un suivi post-natal rapproché était mis en place.

#### Fièvre jaune

Le virus de la fièvre jaune (FJ) est un flavivirus originaire d'Afrique importé par le commerce triangulaire et circulant à la fois sur le continent africain (95%) et sud-américain où il est endémique [60]. Il s'agit d'une arbovirose potentiellement grave se manifestant par un syndrome hépatorénal hémorragique. Elle peut être responsable de grandes épidémies comme cela a été le cas dans les États de Sâo Paulo et de Rio de Janeiro au Brésil en 2016-2018, ou plus récemment en 2021 au Venezuela [148, 318]. En Guyane, aucun cas humain de FJ n'avait été signalé de 1902 – date du début de la surveillance – à 1998 – date à laquelle un premier cas mortel avait été rapporté chez une Amérindienne wayana du Haut Maroni [167]. Ensuite, aucun cas n'a été rapporté pendant 20 ans jusqu’à 2017. Quatre cas ont été rapportés depuis, 2 cas chez des ressortissants brésiliens travaillant sur des camps d'orpaillage clandestins en forêt en 2017 et 2020, 1 cas chez un adolescent Amérindien wayana du Haut Maroni en 2020 également, bien que vacciné contre la fièvre jaune dans l'enfance mais n'ayant pas reçu de rappel après 6 ans et co-infecté par la Covid-19, et enfin 1 cas chez un citoyen suisse de 47 ans non vacciné [329, 362]. Tous ces cas se sont avérés fatals, ce qui laisse supposer une sous-détection de la circulation de ce virus. Un certificat international de vaccination anti-amarile est obligatoire pour les résidents de la Guyane et les voyageurs souhaitant s'y rendre. Le certificat est valable à vie après une seule injection (OMS, 11 juillet 2016), à l'exception des personnes immunodéprimées, des personnes se rendant dans un pays où une circulation active du virus est signalée, des femmes ayant été vaccinées en cours de grossesse et des enfants de plus de 6 ans ayant reçu leur première injection avant l’âge de 2 ans [334]. Le vaccin est recommandé à partir de 9 mois chez les enfants se rendant ou vivant en pays à risque.

#### Mayaro

Le virus Mayaro (MAYV) est un alphavirus de la famille des Togaviridae proche du virus du chikungunya et décrit pour la première fois à Trinidad en 1954. Son principal vecteur est un moustique selvatique *Haemagogus* spp., mais *Ae. aegypti* a également été incriminé dans la transmission à l'hôte humain. MAYV a déjà provoqué plusieurs épidémies en région amazonienne au Venezuela, au Pérou, en Bolivie et au Brésil. Il a été isolé pour la première fois en Guyane en 1996 [352]. Une vaste étude a récemment montré une séroprévalence pouvant aller de 1% à Cayenne jusqu’à 23,5% dans certaines communes isolées du haut Oyapock et du haut Maroni [19]. Une étude rétrospective a identifié 17 cas humains entre 2003 et 2019, majoritairement acquis en forêt profonde [255]. Le tableau clinique et biologique était proche de celui du chikungunya avec de la fièvre et des arthralgies. Un patient a eu une méningo-encéphalite aiguë et 4 autres ont souffert d'arthralgies persistantes. En 2020, un cluster d'une quinzaine de cas a été identifié à Matoury, avec des tableaux cliniques aigus et chroniques très évocateurs de chikungunya, mais finalement positifs en biologie moléculaire à MAYV. MAYV doit donc être évoqué devant un tableau d'arthralgie fébrile chez des patients vivant ou revenant d'Amérique latine. Cet arbovirus avait également été évoqué, avant l'arrivée de la Covid-19, comme un virus à potentiel pandémique [4].

#### Tonate

Le virus Tonate (TONV) est également un arbovirus de la famille des Togaviridae et du genre *Alphavirus*, régulièrement décrit en Guyane. Il appartient au sous-type IIIb du complexe des virus de l'encéphalite équine vénézuélienne. Il a été décrit pour la première fois en 1973 en Guyane chez un oiseau, le cacique huppé, dans le village de Tonate, près de Cayenne, puis retrouvé chez plusieurs espèces de moustiques en Guyane et au Suriname, y compris *Anopheles, Culex* et *Lutzomyia* [94]. Il a par la suite été retrouvé chez plusieurs espèces d'oiseaux guyanais et récemment chez quatre espèces de chauves-souris [128]. Enfin il a été isolé de puces de nids d'hirondelles dans le Colorado et l'Utah. Si l'on retrouve quelques publications humaines chez des patients guyanais, dont les premiers sont 2 cas rapportés en 1973 et 1975, cette infection humaine n'a jamais été rapportée en dehors de Guyane. Deux études sérologiques auprès de la population, l'une dans les années 1970 et l'autre dans les années 1990, ont montré des taux de séroprévalence moyens autour de 11-14%, avec de très grandes variations géographiques de 0 à 35%, les taux les plus importants étant retrouvés dans les plaines du littoral [95, 354]. Une étude rétrospective a été menée sur les cas identifiés au CNR des arbovirus de Cayenne entre 2003 et 2016, qui a identifié 45 cas [252]. L'infection touchait principalement des hommes jeunes et les symptômes les plus fréquemment retrouvés étaient la fièvre, les frissons, les céphalées et les douleurs diffuses. Comme pour MAYV, le bilan biologique était peu spécifique avec une lymphopénie dans environ 20% des cas et une CRP supérieure à 50 mg/l dans 20% des cas également. Un tableau de méningo-encéphalite aiguë à liquide pléiomorphe associé à une hyperprotéinorachie à 1,52 g/l, d’évolution spontanément favorable était rapporté. Aucun décès n’était à déplorer. Le seul cas grave existant dans la littérature est celui d'une encéphalite mortelle chez une enfant de 2 mois publiée en 1997. Récemment, l’équipe de Saint-Laurent-du-Maroni a rapporté pour la première fois un cas de transmission verticale du virus Tonate chez une femme enceinte de Guyane. Le fœtus présentait des lésions nécrotiques et hémorragiques sévères au niveau du cerveau et de la moelle épinière [199]. TONV doit donc être évoqué devant un tableau dengue-like, ainsi que devant un tableau d'infection du SNC non documenté. Il est à noter qu'une sérologie positive en IgM anti-TONV ne signe pas l'infection, puisque la majorité des 326 sérologies positives de l’étude 2003-2016 avait finalement un diagnostic alternatif à ce virus [252].

#### Oropouche

Le virus Oropouche (OROV) est un arbovirus de la famille des Bunyaviridae identifié pour la première fois chez l’être humain en 1955 à Trinidad et Tobago, généralement transmis par des *Culicoides* sp., sortes de moucherons. Plusieurs épidémies ont été rapportées en Amérique latine en particulier au Brésil, au Pérou et en Équateur [325]. En août et septembre 2020, en pleine épidémie de Covid-19, une cinquantaine de cas a été rapportée pour la première fois en Guyane chez les habitants du petit village de Saül, situé en plein cœur de la forêt amazonienne, avec un taux d'attaque estimé entre 43 et 61%, sans que l'origine ni le vecteur de cette épidémie ne soient évidents [139]. La symptomatologie de ce virus est aspécifique, (fièvre, céphalées et douleurs diffuses) et rarement pourvoyeuse de cas graves. L’épidémie de Saül en août-septembre 2020 a entraîné 3 hospitalisations, dont une pour méningite aiguë lymphocytaire, toutes d’évolution favorable.

#### *Aedes aegypti* et arboviroses

**Figure 13:**
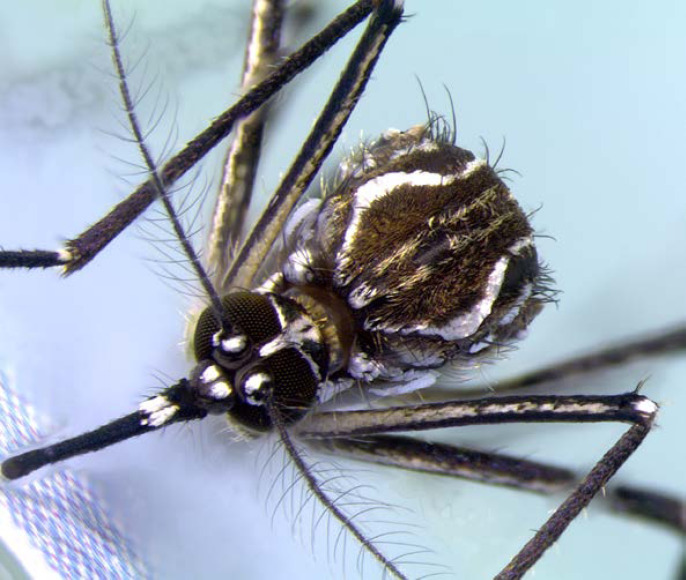
*Aedes aegypti* (crédit photo: Institut Pasteur de la Guyane; photo: P. Gaborit) Aedes aegypti *(photo credit: Institut Pasteur de la Guyane; photo: P. Gaborit)*

Les arbovirus présents en Guyane partagent des spécificités communes:

- DENV, CHIKV et ZIKV sont transmis par un vecteur commun, *Ae. aegypti* (Fig. 13). Il s'agit d'un vecteur urbain et anthropophile très implanté sur le territoire et en continuelle extension. Les études de séroprévalence ont mis en avant la forte atteinte des populations vivant sur le Maroni qui avaient longtemps été épargnées par ces arbovirus [232]. La présence de ce vecteur à travers tout le territoire tend à favoriser le risque de nouvelles épidémies (DENV en particulier par ses 4 sérotypes) mais aussi de nouvelles introductions d'arbovirus comme cela a déjà été le cas pour ZIKV et CHIKV.
- Au décours des épidémies de CHIKV et ZIKV, une surveillance active des arboviroses a été poursuivie, mettant en évidence l'absence d'installation d'une circulation endémique de ces virus.
- Comme beaucoup de maladies infectieuses, les maladies vectorielles ont un fort impact sur les populations les plus précaires. Cette problématique est prioritaire en Guyane avec 50% de la population vivant sous le seuil de pauvreté. La précarité a été décrite comme un facteur de risque d'infection par ZIKV et par CHIKV, en particulier en début d’épidémie [34, 157]. Il s'agit d'une population souvent plus exposée aux vecteurs et plus éloignée des campagnes ou actions de prévention, comme le projet WASH mis en place par la Croix-Rouge française (CRf) pour lutter contre les maladies hydriques et à transmission vectorielle dans les quartiers défavorisés [267].

#### Projet WASH

Margot Oberlis, Loïc Epelboin

L'acronyme anglais WASH pour WAter, Sanitation and Hygiene correspond aux programmes qui interviennent sur l'accès à l'Eau, à l'Assainissement et à l'Hygiène dont les acronymes français sont EAH ou EHA [267]. Ces programmes sont généralement déployés dans les pays du Sud, dans les régions avec des accès difficiles à l'eau potable mais aussi lors de catastrophes naturelles ou de situations d'urgence humanitaire. Mené par la CRf en Guyane, le projet WASH est né d'une instruction ministérielle de mars 2020 concernant la prise en charge et le soutien des populations précaires face à l’épidémie de Covid-19 et qui préconisait un accès à l'eau et aux produits d'hygiène de première nécessité. Ainsi de mi-septembre 2020 à fin mai 2022, ce projet s'est développé dans 2 zones principales: les habitats informels des villes du littoral et les communes isolées de l'intérieur le long des fleuves frontières. Un état des lieux a d'abord été réalisé concernant l'accès à l'eau potable dans les zones indiquées, profitant de la mise à disposition de différents systèmes d'approvisionnement gratuit de l'eau à l'occasion de la crise Covid, puis une cartographie des acteurs et la définition de problématiques prioritaires. De nombreuses actions ont été menées, à la fois sur la thématique de la Covid et sur celles des maladies à transmission hydrique et vectorielle, à l'aide d'agents de terrain, issus des communautés et formés à la promotion de la santé avec une approche communautaire et à la médiation en santé: distribution de kits d'hygiène, enquêtes sur les connaissances EHA, maraudes de sensibilisation sur les problèmes de santé publique, animations collectives, aide à la création de collectifs de quartiers et interventions ponctuelles en cas de survenue de cas de certaines pathologies dans les quartiers, telles que la leptospirose ou le syndrome pulmonaire à hantavirus, rendant les populations-cibles actrices du dispositif. Ce projet a rencontré un vif engouement auprès des populations, des différents partenaires institutionnels et des autorités sanitaires et administratives. La première partie du projet a pris fin en mai 2022 mais une réflexion commune entre la CRf et l'ARS est en cours afin de redéployer un projet pérenne répondant aux besoins innombrables du territoire en lien avec les problématiques EHA.

### Viroses respiratoires

#### Grippe

Antoine Enfissi, Luisiane Carvalho

Contrairement aux idées reçues, des épidémies de grippe saisonnière surviennent chaque année dans les régions tropicales. Leurs caractéristiques épidémiologiques sont toutefois différentes de celles observées en France hexagonale [169]. Une étude réalisée en Guyane de 2011 à 2016 montre qu'elles débutent après les épidémies de l'Hexagone (environ 1 mois) et qu'elles durent plus longtemps (22 semaines en Guyane *versus* 9 semaines dans l'Hexagone) [179]. La diversité virale rencontrée s'explique par les liens de la Guyane avec l'Europe et le continent sud-américain. Entre 2015 et 2018, pour chaque épidémie saisonnière (d'une durée comprise entre 13 et 23 semaines), entre 7 990 et 11 640 consultations pour syndrome grippal ont été estimées, 2 à 5 cas graves ont été admis en réanimation, et 0 à 1 décès ont été comptabilisés parmi ces cas graves. Il n'y a pas eu d’épidémie de grippe en Guyane en 2019 et 2020, probablement grâce aux multiples précautions individuelles et collectives prises dans le cadre de l’épidémie de Covid-19. L’épidémie saisonnière de grippe 2021-2022 s'est déroulée de fin décembre 2021 à courant mars 2022, soit une durée de 11 semaines, plus courte que les épidémies passées. Cette épidémie s'est caractérisée par la co-circulation de virus grippaux uniquement de type A, avec une majorité de prélèvements positifs au virus A/H3N2 (97%). Au total, 3 cas graves de grippe ont été admis en réanimation durant l’épidémie, il s'agissait de patients présentant des comorbidités [333]. Ces patients ont évolué favorablement. La vaccination reste le moyen le plus efficace de se prémunir des formes graves de la grippe et la campagne de vaccination en Guyane pourrait être adaptée aux caractéristiques du territoire.

#### Covid-19

Luisiane Carvalho, Cyril Rousseau

Bien qu'en partie protégée des formes graves par son jeune âge (50% de la population a moins de 25 ans), la population de Guyane a été confrontée à un fardeau infectieux inhabituel à l'occasion de la pandémie de Covid-19. La mortalité hospitalière observée est équivalente à près d'un semestre de mortalité « toutes causes confondues » et à 10 années de mortalité par accident de la voie publique. Pour un territoire de 300 000 habitants doté d'une offre sanitaire limitée, quoique aux normes européennes, le fardeau, représenté par près de 6 000 hospitalisations dont 800 en réanimation et plus de 400 décès quasi-exclusivement hospitaliers, a été majeur.

**Figure 14:**
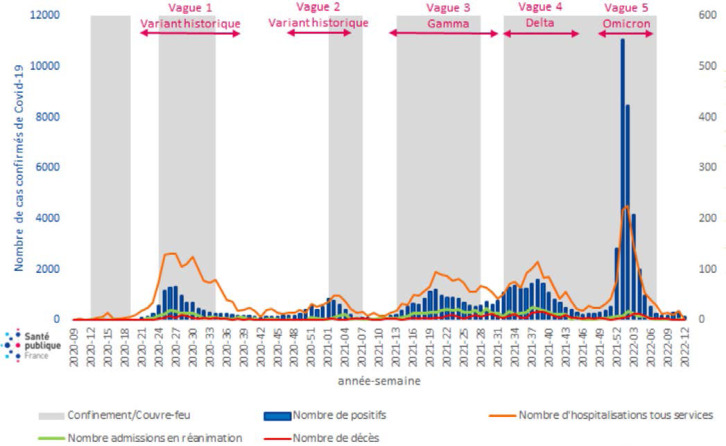
Évolution hebdomadaire du nombre de cas confirmés de Covid-19, du nombre d'hospitalisations tous services confondus et du nombre de décès à l'hôpital, Guyane, mars 2020 à mars 2022. Données SIDEP, SIVIC: Santé publique France en Guyane Weekly evolution of confirmed Covid-19 cases, hospital admissions including all wards, and in-hospital deaths, French Guiana, March 2020 to March 2022. SIDEP and SIVIC data, Santé publique France in French Guiana

Six vagues épidémiques se sont succédé jusqu’à fin 2022 au gré des importations de variants et des vagues de l'environnement latino-américain [234, 331] (Fig. 14). Les 3^e^ et 4^e^ vagues (dues aux variants Gamma puis Delta) ont rythmé les trois quarts de l'année 2021 avec un impact important sur le fonctionnement du système de santé guyanais et une mortalité hospitalière de 9,2 à 13,1% (respectivement pour Gamma et Delta). Le nombre de cas détectés durant ces deux vagues a été de 28 000, mais les études de séroprévalence ont montré que le nombre de personnes immunisées était probablement 4 fois plus élevé [129].

En effet, le jeune âge de la population ne fait pas tout. Les facteurs de vulnérabilité des Guyanais sont nombreux: forte prévalence du surpoids et de l'obésité morbide, du diabète (souvent méconnu ou non contrôlé), des maladies cardiovasculaires, dont l’âge de survenue est plus précoce d'une dizaine d'années par rapport à l'Hexagone [147]. Ainsi, les formes sévères de Covid-19 étaient hospitalisées en moyenne à 50 ans, en réanimation à 60 ans, et décédaient à un âge proche de 70 ans, bien inférieur à ceux observés dans l'Hexagone [9]. En dépit de ces fragilités, et de leur éloignement d'une partie de la population, les structures de santé guyanaises ont permis de limiter l'impact sanitaire, aidées par une politique volontariste et innovante d’« aller vers » en déployant une offre de dépistage au plus près de la population, puis de vaccination, mais aussi d'oxygénothérapie à domicile [123]. Cela a grandement contribué à éviter le désastre sanitaire observé dans nombre d’États voisins comme le Brésil, le Suriname ou l’Équateur, qui n'ont souvent pas été en mesure de massifier leur offre en soins critiques et en oxygène, lorsque la Guyane augmentait ses capacités de 500% durant plusieurs mois [263]. Outre les renforts obtenus, un des facteurs de résilience a aussi été l'expérience acquise sur un territoire qui connaît de longue date les épidémies récurrentes d'arboviroses.

Si le futur reste incertain, l'immunisation vaccinale reste insuffisante pour protéger les plus fragiles (un Guyanais sur deux, au-delà de 50 ans, a un schéma vaccinal complet, et à peine 41% de la population des plus de 12 ans a reçu 2 doses au mois de décembre 2022), tandis que l'hésitation vaccinale a aussi concerné les soignants [373], même si la Guyane a une séro-immunité parmi les plus élevées de France (63,9% en août 2021) [129]. Dans le futur, les plus vulnérables resteront exposés au risque de forme sévère. Ceci d'autant plus que les inégalités de santé, très présentes en Guyane, pour les populations les plus isolées et en situation de précarité, sont de puissants déterminants de l'accès au dépistage, aux soins et à la vaccination [21, 307].

### Autres infections virales aiguës

#### Syndrome pulmonaire à hantavirus

Loïc Epelboin, Hatem Kallel, Anne Lavergne

**Figure 15:**
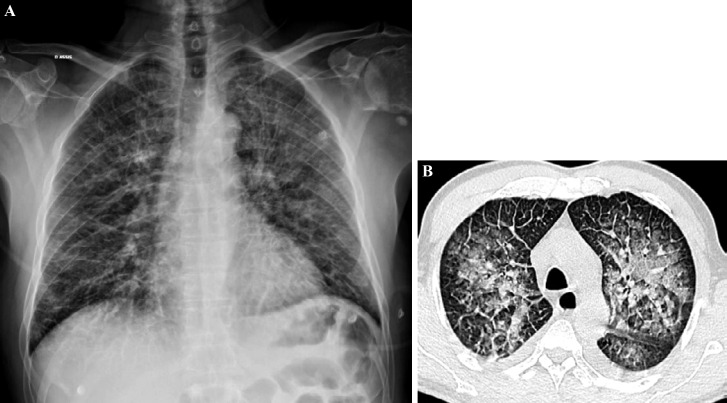
Patient âgé de 55 ans admis en réanimation pour prise en charge d'un syndrome pulmonaire à hantavirus compliqué d'une défaillance multiviscérale (respiratoire, rénale et hématologique) (crédit photo: M. Zappa) Légende: La radiographie pulmonaire (A) et le scanner thoracique (B) (fenêtre parenchymateuse passant par les lobes supérieurs) montrent des condensations alvéolaires et verre dépoli en plage prédominant dans les régions centrales associés à des lignes septales. Évolution favorable sous traitement symptomatique de réanimation et antibiothérapie. 55-year-old patient admitted in intensive care unit for management of a pulmonary hantaviral syndrome complicated by multivisceral failure (respiratory, renal and hematological) (photo credit: M. Zappa) Chest X-ray (A) and CT scan (B) (parenchymal window through the upper lobes) show alveolar condensations and ground glass predominantly in the central regions associated with septal lines. Favorable evolution under symptomatic resuscitation treatment and antibiotic therapy.

**Figure 16:**
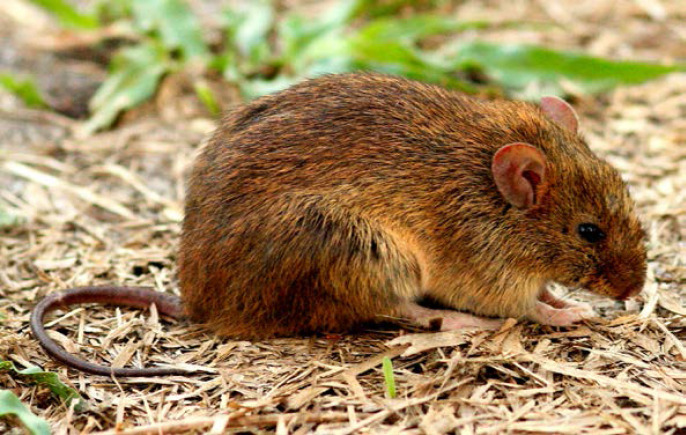
Rat des cannes à queue courte (*Zygodontomys brevicauda*), vecteur de l'hantavirus Maripa, pripris (marais) de Yiyi, Sinnamary (crédit photo: A. Baglan) *Short-tailed cane mouse* (Zygodontomys brevicauda), *vector of Maripa hantavirus, Yiyi pripris (marshes), Sinnamary (photo credit: A. Baglan)*

Les hantavirus sont des virus à ARN appartenant à l'ordre des *Bunyavirales*, à la famille des Hantaviridae et au genre *Hantavirus.* On distingue les hantavirus de l'Ancien Monde, responsables de fièvres hémorragiques avec syndrome rénal (FHSR) en Europe et en Asie, de ceux du Nouveau Monde, décrits pour la première fois en 1993, et responsables du syndrome pulmonaire (ou cardio-pulmonaire) à hantavirus (HPS). Ce syndrome associe une atteinte respiratoire fébrile hypoxémiante diffuse précédée de prodromes à type de myalgies et de troubles digestifs très marqués à cette phase. Il évolue vers le décès dans la moitié des cas, en 1 semaine en moyenne sur les séries nord-américaines [106]. Le tableau biologique se caractérise par un syndrome inflammatoire et une thrombopénie, sans la classique atteinte hémorragique retrouvée dans l'Ancien Monde [131]. La cytolyse, présente ailleurs dans les descriptions sudaméricaines, n'est pas retrouvée en Guyane [224]. L'atteinte respiratoire est très marquée, avec un tableau similaire à un œdème pulmonaire en rapport avec une fuite capillaire [191] (Fig. 15). L'atteinte rénale survient généralement secondairement, chez les patients qui survivent, comme observé chez les cas de 2022. En 2006 en Guyane, une première étude sérologique retrouvait une séroprévalence de 1,4% pour ces virus [226]. Entre 2008 et 2022, 11 cas (dont 4 cas entre mars et septembre 2022) de syndromes pulmonaires gravissimes dont 5 décès survenus en moins de 24 heures ont été attribués à un virus secondairement nommé virus Maripa, du fait d'une divergence importante des nucléotides et des acides aminés par rapport aux souches connues [223]. Le séquençage du génome a finalement montré que le virus Maripa était proche du virus Rio Mamoré, dont des souches avaient précédemment été rapportées au Paraguay, au Pérou, en Bolivie et dans la région amazonienne de l’État du Maranhão au Brésil, et il a récemment été classifié comme appartenant à l'espèce *Laguna Negra* [196, 225]. Les hantavirus pathogènes sont transmis par des rongeurs par excrétion dans le milieu extérieur de fèces et d'urines, comme en témoigne en Guyane leur présence chez 2 espèces de rongeurs *Oligoryzomys delicatus* et *Zygodontomys brevicauda* (Fig. 16) capturés près des maisons de 2 des patients atteints en 2009 et 2013 [83]. De nombreux rongeurs d'espèces communes urbaines comme le rat noir (*Rattus rattus*), le surmulot (*Rattus norvegicus*), la souris grise (*Mus musculus*) ainsi que le rat de Cayenne (*Proechimys cayennensis*) ont été testés et étaient tous négatifs. Une enquête de séroprévalence est actuellement en cours dans les quartiers informels où les cas ont été rapportés début 2022.

#### Rage

Brigitte Roman-Laverdure, Loïc Epelboin

**Figure 17:**
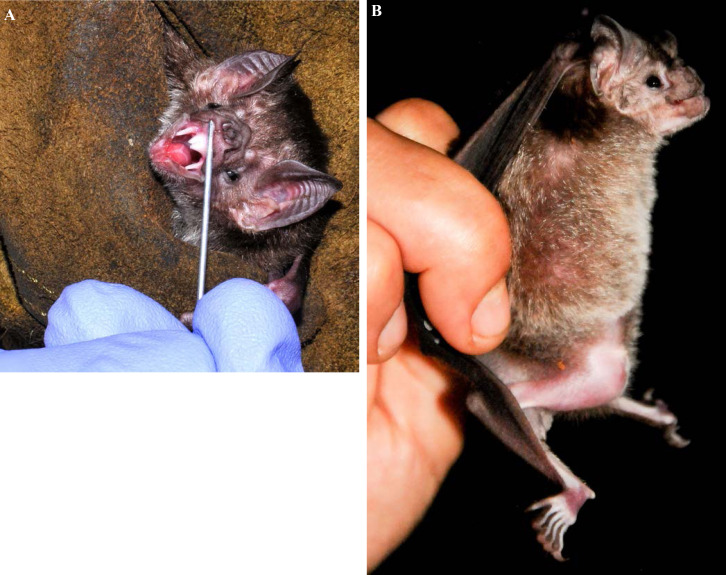
A. Prélèvement de salive pour recherche du virus de la rage par l'Institut Pasteur de la Guyane. Vampire commun *(Desmodus rotondus)*, vecteur de la rage en Guyane. B. *D. rotondus* capturé lors d'une mission inventaire dans la Réserve du Mont Grand Matoury (crédit photo: L. Epelboin) *A. Saliva collection for rabies virus testing by Institut Pasteur in French Guiana. Common vampire bat* (Desmodus rotondus), *vector of rabies in French Guiana. B.* D. rotondus *captured during an inventory mission in Mount Grand Matoury Reserve (photo credit: L. Epelboin)*

**Figure 18:**
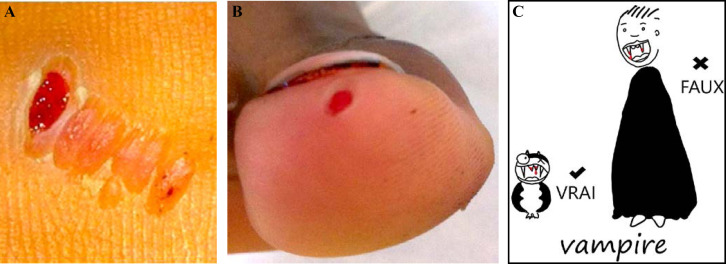
A. Morsure de vampire commun sur le talon (4 morsures consécutives) et B. Morsure sur un gros orteil (crédit photo: L. Epelboin). C. Dents utilisées pour la morsure (Illustration: É. Mosnier) A. Common vampire bite on the heel (4 consecutive bites) and B. Bite on a big toe (photo credit: L. Epelboin). C. Teeth used for biting (Illustration: É. Mosnier)

Encéphalomyélite virale due à différents génotypes viraux du genre *Lyssavirus*, la rage est presque toujours mortelle pour l'Humain et la plupart des mammifères atteints. Il s'agit d'une zoonose accidentellement transmissible à l’être humain. En Guyane, seul le génotype 1 (RABV) circule chez les chauves-souris et les mammifères terrestres, contrairement à l'Europe où le génotype 1 est retrouvé chez les chiens, les renards et autres mammifères terrestres, tandis que les génotypes 5 et 6 sont identifiés chez les chauves-souris (European bat Lyssavirus 1 & 2) [348]. Entre 1989 et 2022, le virus a été identifié à 16 reprises en Guyane chez 14 mammifères non volants (10 bovins, 3 chiens, 1 chat), 2 chauves-souris (*Artibeus planirostris*, une chauve-souris frugivore, et *Desmodus rotondus*, le vampire commun) et 1 homme qui en est décédé en 2008. Sa contamination a été attribuée à des chatons malades qui auraient été mordus par *D. rotondus* [233]. La prise en charge des expositions à risque rabique repose sur un centre antirabique (CAR) situé au Centre hospitalier de Cayenne où sont disponibles les immunoglobulines et les vaccins antirabiques, et sur des antennes antirabiques (AAR) réparties dans différents CDPS de Guyane ainsi qu’à Kourou et Saint-Laurent-du-Maroni. De nouveaux schémas vaccinaux proposés par l'Organisation mondiale de la santé (OMS) et validés par la Haute autorité de santé (HAS) en 2018 ont récemment été mis en place au sein du CAR selon les recommandations des groupes d'experts du Haut Conseil de la santé publique (HCSP). Ils permettent de raccourcir la durée de prise en charge des protocoles notamment en post-exposition mais aussi en pré-exposition. Ainsi dorénavant, en plus des injections intramusculaires et intralésionnelles d'immunoglobulines antirabiques, le vaccin est administré par voie intradermique, à raison de 2 doses (de 0,1 ml de vaccin) à J0, J3 et J7 (une injection par site) au lieu des habituelles injections IM d'une dose de vaccin complet à J0, J7 et J21-28. En pré-exposition, le protocole est de 2 injections intradermiques à J0 et J7. Et en cas d'exposition, pour une personne ayant déjà reçu auparavant au moins 2 doses de vaccin antirabique (soit lors d'une précédente exposition, soit en pré-exposition), elle reçoit 4 injections intradermiques à J0 ou une injection intradermique à J0 et à J3 au lieu d'une injection intramusculaire à J0 et à J3. Ce protocole reste accompagné d'un contrôle sérologique à distance. Seuls les chiroptérologues continuent de recevoir les injections en IM à J0 et J3 (Tableau II). La décision d'administration de la prophylaxie post-exposition est prise par un médecin au CAR en fonction de la gravité des lésions, de l'animal mordeur, des circonstances de l'incident et de son comportement, ainsi que de la possibilité d'effectuer une surveillance d'une durée réglementaire pour celui-ci ou un examen de laboratoire s'il est mort, abattu ou euthanasié. Le schéma vaccinal à appliquer sera ajusté en fonction de l’état de santé de la personne, des éventuels antécédents de vaccination antirabique et des derniers contrôles sérologiques effectués s'il s'agit d'un professionnel à risque d'exposition. Une étude réalisée en 2014 a montré que si la faune à l'origine d'une prise en charge du risque antirabique était assez variée en Guyane, les chiens représentaient 58,4% des consultations et les chauves-souris 23,8% (vs 59% et 5% respectivement en France) [124]. En effet, parmi la centaine d'espèces de chauves-souris identifiées en Guyane, 2 des 3 espèces de chauves-souris vampires hématophages d'Amérique latine sont retrouvées, parmi lesquelles seul *D. rotondus*, le vampire commun, est impliqué dans les morsures sur des humains (Fig. 17). Ces morsures surviennent le plus souvent la nuit, sur des personnes dormant en hamac sans moustiquaire ou dans des moustiquaires insuffisamment amples. La morsure est généralement indolore et la personne se réveille avec l'impression de baigner dans son sang, suite au saignement abondant lié à l'injection par l'animal de substances anticoagulantes. Si le risque de se faire mordre est globalement faible en Guyane, certains villages, notamment le long du fleuve Maroni, sur les sites d'orpaillage et certains sites touristiques en forêt, sont connus pour être propices. Ainsi, pour le voyageur venant visiter la Guyane, la vaccination pré-exposition peut être proposée en cas de séjour aventureux prévu en forêt ou sur le fleuve Maroni avec nuits en hamac. La moustiquaire adaptée au hamac reste le meilleur moyen d’éviter ces morsures, quoique le risque puisse persister, en cas d'orteil ou de coude malencontreusement collé à la moustiquaire pendant la nuit (Fig. 18).

**Tableau II:** Récapitulatif des schémas vaccinaux en pré- et post-exposition au centre antirabique du CHC *Summary of pre- and post-exposure vaccination schemes* at the anti-rabies centre of the CHC

**Figure:** 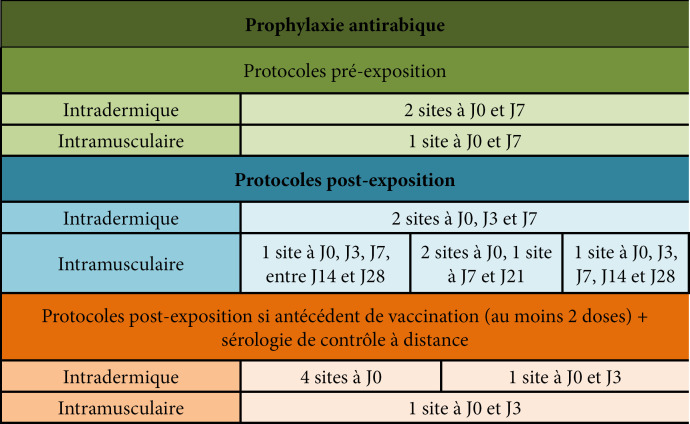

### Protozooses d'importance

#### Paludisme

Maylis Douine, François Delon

**Figure 19:**
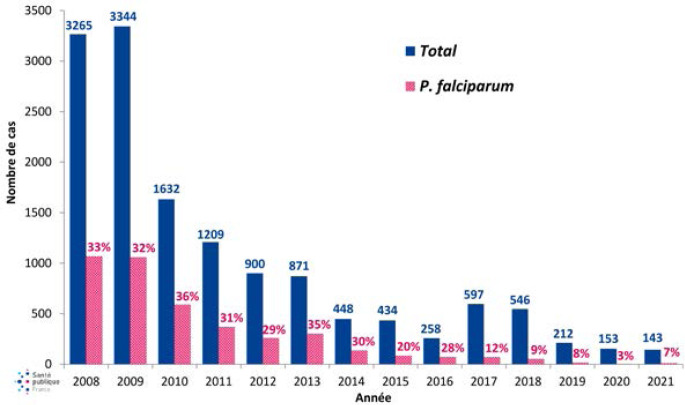
Nombre total d'accès palustres et part d'accès dus à *P. falciparum* diagnostiqués chaque année par les laboratoires de ville et hospitaliers, les centres de santé et l'Armée, Guyane, janvier 2008 à décembre 2021 (source: CDPS/CH Cayenne, LBM de Guyane, FAG - Exploitation: L. Carvalho, Santé publique France en Guyane) *Total number of malaria attacks and proportion of attacks due to* P. falciparum *diagnosed each year by city and hospital laboratories, health centres and the army, French Guyana, January 2008 to December 2021 (source: CDPS/CH Cayenne, LBM de Guyane, FAG - Analysis: L. Carvalho, Santé publique France en Guyane)*

**Figure 20:**
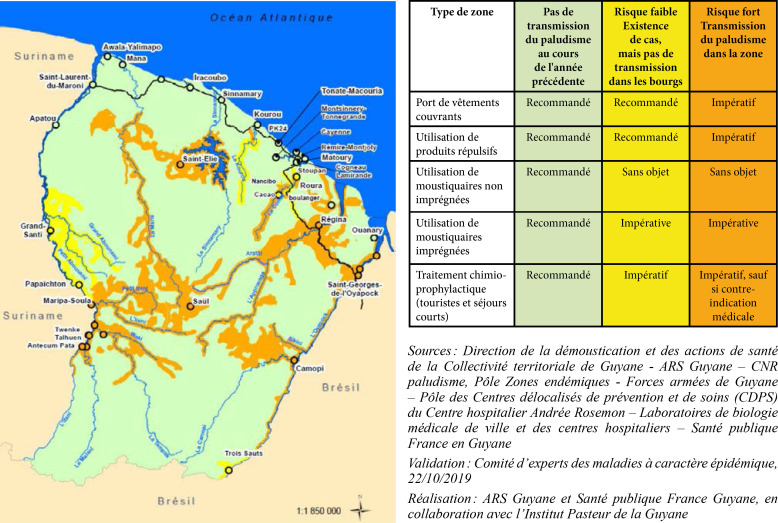
Carte du risque de paludisme en Guyane en 2019 (évalué selon les données épidémiologiques 2018) *(source: Rapport 2021 du CNR paludisme)* Map of malaria risk in French Guiana in 2019 (evaluated according to 2018 epidemiological data) (source: National Reference Center Malaria report 2021) Sources: Direction de la démoustication et des actions de santé de la Collectivité territoriale de Guyane – ARS Guyane – CNR paludisme, Pôle Zones endémiques – Forces armées de Guyane – Pôle des Centres délocalisés de prévention et de soins (CDPS) du Centre hospitalier Andrée Rosemon – Laboratoires de biologie médicale de ville et des centres hospitaliers – Santé publique France en Guyane Validation: Comité d'experts des maladies à caractère épidémique, 22/10/2019 Réalisation: ARS Guyane et Santé publique France Guyane, en collaboration avec l'Institut Pasteur de la Guyane

Historiquement endémique en Guyane, le paludisme y devient de plus en plus rare grâce à l'amélioration de l'accès au diagnostic et au traitement (notamment les dérivés de l'artémisinine, avec une diminution plus rapide des gamétocytes et donc un impact sur la transmission), au déploiement des tests diagnostiques rapides, à un programme orienté spécifiquement vers la population des orpailleurs clandestins (projet Malakit, www.malakit-project.org) et à la réalisation d'actions ciblées et à d'autres programmes de recherche comme Elimalar/Palustop [100, 249]. La transmission du paludisme reste possible à faible niveau toute l'année dans la région de Saint-Georges/Régina, dans les villages amérindiens du haut Maroni ainsi que sur les sites d'orpaillage clandestins où l'accès aux soins reste difficile et l'automédication très élevée [243]. Le nombre de cas par an était de 141 en 2021 contre plusieurs milliers dans les années 2000 [332] (Fig. 19). Aucun décès n'a été signalé depuis 2013. La France s'est engagée dans l’élimination du paludisme sur son territoire en 2025. Bien qu'encore mentionnée dans les recommandations aux voyageurs du Haut conseil de la santé publique, une prophylaxie n'apparaît donc nécessaire qu'en cas de déplacement dans des zones à risque, en situation d'isolement en forêt ou dans des conditions de séjour à risque particulier (par exemple, les militaires) (Fig. 20) [334]. Actuellement l'espèce prédominante est *Plasmodium vivax* (> 90%), les autres cas étant principalement liés à *P. falciparum*, à présent majoritairement importés. Le moustique *Anopheles darlingi* est le principal vecteur, mais d'autres espèces sont impliquées notamment en forêt avec des comportements encore mal connus [51, 109]. Le diagnostic se fait par des tests de diagnostic rapide différenciant *P. falciparum* des autres espèces, confirmé ensuite par microscopie et/ou biologie moléculaire. Le traitement du *P. falciparum* repose sur la combinaison artéméther-luméfantrine pour les formes simples (l'association pipéraquine-dihydroartémisine n'est pas distribuée en Guyane du fait de la détection de parasites de sensibilité diminuée) et artésunate IV pour les formes graves. La quinine IV est exceptionnellement utilisée pour les patients présentant des vomissements incoercibles et les femmes enceintes. Les accès à *P. vivax* étaient jusqu’à récemment traités par chloroquine à la dose de 25 mg/kg sur 3 jours (10-10-5 mg/kg/j), mais la fin de la commercialisation fin 2022 a nécessité de modifier les protocoles avec un traitement par dérivés de l'artémisine dorénavant administré [121]. Un traitement radical par primaquine doit être proposé à la dose 0,5 mg/kg/j (maximum 30 mg/j) pendant 14 jours afin d’éradiquer les formes quiescentes hépatocytaires et éviter les reviviscences. Celui-ci ne peut être proposé qu'après élimination d'un déficit en G6PD (risque d'hémolyse) puis est remis sur ATU de cohorte ce qui rend ce traitement radical difficile d'accès en pratique pour les populations isolées. Jusqu’à peu il était recommandé 14 jours après le début du traitement de l'accès palustre pour doser l'activité G6PD sans risquer que l'hémolyse perturbe ce dosage. Une étude rétrospective menée dans le cadre d'une thèse soutenue récemment semble indiquer qu'il serait sans risque pour le patient réaliser le dosage à J3 [76]. En cas de déficit en G6PD avec une activité inférieure à 30% chez l'homme et intermédiaire (entre 30 et 80%) chez la femme, il est recommandé que le traitement soit prescrit à la posologie de 0,75 mg/kg/semaine pendant 8 semaines, uniquement s'il existe une possibilité de suivi rapproché du patient ainsi que de transfusion rapide en cas d'anémie aiguë. La primaquine est aussi contre indiquée pendant la grossesse, l'allaitement et chez l'enfant de moins de 6 mois. Bien que devenu rare, il reste important d’évoquer ce diagnostic devant toute fièvre et ne pas hésiter à réaliser un test de diagnostic rapide, simple et peu coûteux, à compléter pour confirmation par un test diagnostic par une méthode sensible (goutte épaisse ou PCR) associé à un diagnostic d'espèce (frottis ou PCR d'espèce), étant donné la gravité potentielle de la maladie.

### Toxoplasmose amazonienne

Romain Blaizot, Philippe Abboud, Loïc Epelboin

**Figure 21:**
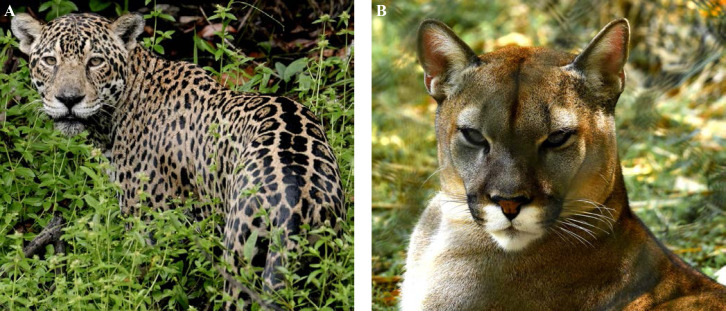
Félidés sauvages hôtes de la souche sauvage de *Toxoplasma gondii.* A. Jaguar *(Pantera onca)* au niveau du lac de Petit Saut (crédit photo: N. Defaux). B. Puma *(Puma concolor)* (crédit photo: L. Epelboin) *Wild felid hosts of* Toxoplasma gondii *wild strain. A. Jaguar* (Pantera onca) *near the lake of Petit Saut (photo credit: N. Defaux). B. Cougar* (Puma concolor) *(photo credit: L. Epelboin)*

**Figure 22:**
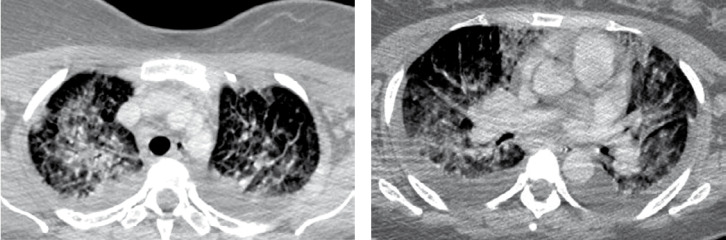
Militaire de 31 ans en mission de lutte contre l'orpaillage illégal, notion de consommation de suidé sauvage en forêt. Toxoplasmose amazonienne à tropisme pulmonaire. Scanner thoracique en fenêtre parenchymateuse montrant des plages de condensation alvéolaire et verre dépoli associés à des lignes septales touchant tous les lobes mais prédominant dans les lobes supérieurs, et un épanchement pleural d'abondance moyenne bilatéral, en rapport avec un SDRA (crédit photo: *M. Zappa)* 31-year-old soldier on a mission against illegal gold mining, notion of consumption of wild suid in the forest. Amazonian toxoplasmosis with pulmonary tropism. Chest CT scan in parenchymal window showing areas of alveolar condensation and ground glass associated with septal lines affecting all the lobes but predominantly in the upper lobes, and a pleural effusion of medium abundance bilaterally, in connection with ARDS (photo credit: M. Zappa)

En Guyane, la toxoplasmose est due à plusieurs souches de *Toxoplasma gondii* impliquées dans 2 cycles différents. On décrit un cycle domestique classique correspondant à la forme clinique habituelle de la maladie, réactivation chez les patients infectés par le VIH et primo-infection chez les femmes enceintes; et un cycle dit sauvage avec les félidés de la forêt amazonienne comme hôte définitif et leurs proies, mammifères herbivores, comme hôte intermédiaire, à l'origine de la toxoplasmose humaine dite amazonienne [53] (Fig. 21). Entre 2002 et 2019, 174 diagnostics de toxoplasmose amazonienne ont été posés en Guyane, parmi lesquels 100 patients avaient été hospitalisés et 74 présentaient des formes paucisymptomatiques, principalement dans les populations autochtones des communes isolées [372]. Les Businenges représentaient 11,5% et les Amérindiens 29,8% de tous les cas décrits. Des épidémies sont régulièrement décrites dans des populations autochtones, notamment sur le haut Oyapock [30, 88]. Ces épidémies représentent 30% des cas rapportés sur la période 2002-2019 [372]. L’être humain se contamine au contact du milieu forestier, le plus souvent en consommant du gibier peu cuit, mais la consommation d'eau de crique (petite rivière amazonienne) semble aussi pouvoir être à l'origine de la contamination [89]. Les cas de toxoplasmose amazonienne rapportés en Guyane semblent dus à des génotypes uniques, atypiques, et plus virulents que les souches habituellement décrites [90]. Cette entité particulière à l'Amazonie se caractérise par un tableau infectieux parfois sévère, parfois sous forme épidémique, résistant aux antibiotiques habituels, au cours d'une primo-infection touchant le sujet jeune immunocompétent et sain. Une étude portant sur 42 cas de toxoplasmose amazonienne diagnostiqués chez des patients des communes isolées entre 2008 et 2015 a montré une prédominance masculine, un âge médian de 16,5 ans, avec 29% d'enfants de moins de 5 ans et une prédominance des cas sur le haut Oyapock [194]. Les signes cliniques les plus fréquemment retrouvés étaient la fièvre > 38 °C (90%), l'asthénie (90%), la polyadénopathie (64%), la toux (44%), les myalgies (20%). La biologie retrouvait une élévation des CPK (73%), une élévation de la troponine (86%), une cytolyse > 2N (72%), des neutrophiles et des lymphocytes normaux (81 et 84%) et une CRP peu élevée (moyenne 17,5 mg/l). Deux tiers des patients ont été hospitalisés, avec une durée moyenne d'hospitalisation de 16 jours, et aucun n'est décédé. On retrouvait au moins 2 organes atteints dans 49% des cas (poumon, foie, cœur, cerveau, œil, notamment). Sur la série de 2002-2019, on retrouvait des CRP plus élevées, notamment chez les patients hospitalisés, avec une moyenne à 65 mg/l [372]. Une atteinte cutanée à type d’éruption diffuse ou de surinfection bactérienne peut être retrouvée, notamment chez les enfants [29]. L'atteinte oculaire était présente dans 7% des cas quand elle était recherchée. Le bilan biologique retrouve souvent une hyponatrémie, un syndrome inflammatoire et une cytolyse hépatique. Le diagnostic repose sur la séroconversion IgM et peut être complété par la PCR *T. gondii.* Son pronostic est lié à l'atteinte pulmonaire, le plus souvent interstitielle diffuse et parfois alvéolaire, qui est de gravité variable, mais potentiellement sévère, avec environ un quart de patients ayant présenté un syndrome de détresse respiratoire aiguë (SDRA) sur une série ancienne, tandis que sur l’étude des patients pris en charge entre 2002 et 2019, seuls 3 patients sur 174 sont décédés, soit moins de 2% [54, 372] (Fig. 22). Une atteinte rétinienne doit être systématiquement éliminée par un examen du fond d’œil. Un traitement par cotrimoxazole ou pyriméthamine-sulfadiazine est recommandé pour une durée mal connue de 3 à 6 semaines. En cas d'atteinte minime, le traitement n'est pas obligatoire, mais il doit être instauré devant un tableau de pneumopathie, plutôt interstitielle, surtout s'il existe des signes de gravité et des facteurs de risque tels que la consommation de gibier et bien sûr toute atteinte oculaire qui doit être systématiquement recherchée par un fond d’œil. Des données récentes montrent que des tableaux de gravité intermédiaire peuvent également être retrouvés, avec des adénopathies fébriles subaiguës sans gravité clinique. Ce tableau a notamment été observé chez des Amérindiens, sans qu'il soit possible de déterminer si cette sévérité intermédiaire était due à des facteurs génétiques de l'hôte ou à des souches moins virulentes.

### Maladie de Chagas ou trypanosomose américaine

Mélanie Gaillet, Philippe Abboud

**Figure 23:**
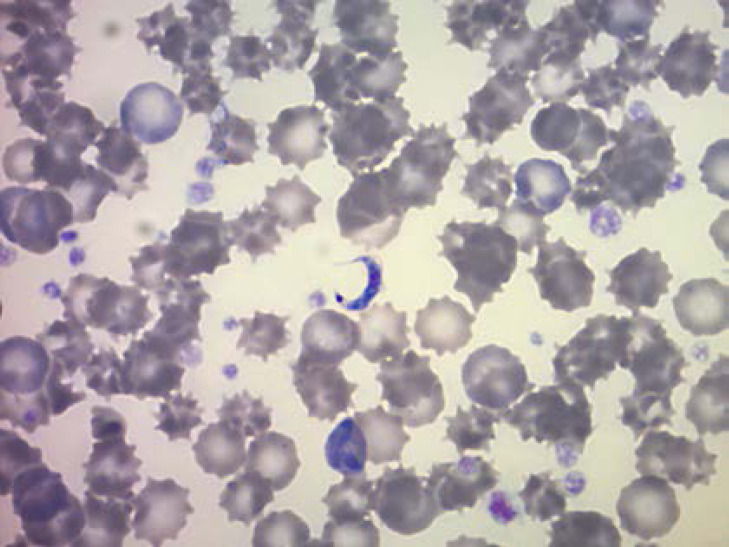
*Trypanosoma cruzi* dans le sang d'un opossum commun *(Didelphis marsupialis).* Coloration au May-Grünwald-Giemsa (crédit photo: D. Blanchet) Trypanosoma cruzi in *the blood of a common opossum* (Didelphis marsupialis). *May-Grünwald-Giemsa staining (photo credit: D. Blanchet)*

**Figure 24:**
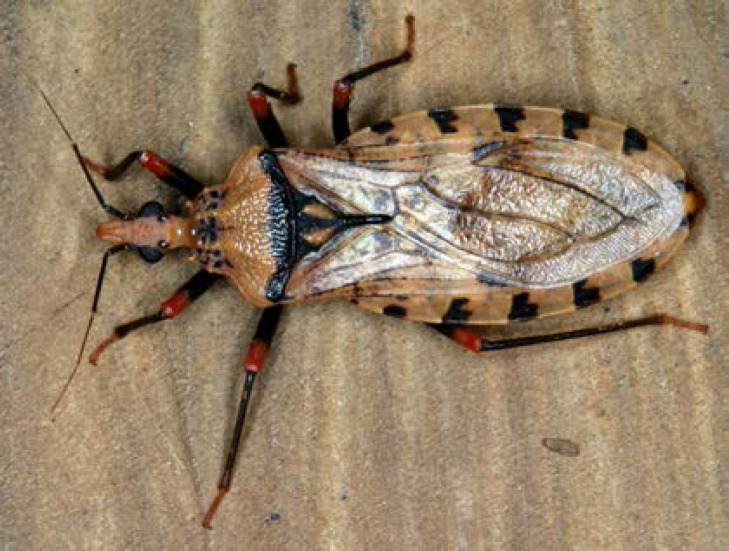
Triatome ou punaise hématophage *(Panstrongylus geniculatus)* de la famille des Reduviidae (crédit photo: D. Blanchet) *Triatomine or hematophagous bug* (Panstrongylus geniculatus) *of the Reduviidae family (photo credit: D. Blanchet)*

La maladie de Chagas ou trypanosomose américaine est une zoonose endémique dans 21 pays d'Amérique due à *Trypanosoma cruzi* [277]. Dans la région amazonienne, cette protozoose a été identifiée au milieu du xx^e^ siècle, avec des cas documentés en Guyane et dans l'Amazonie brésilienne. Cependant, la Guyane n'a été considérée comme endémique qu'après les années 2000 lorsque la transmission de *T. cruzi* y a été mise en évidence, principalement sous la forme de clusters familiaux dus à une contamination orale [33]. Ainsi, de janvier 1990 à mars 2005, 15 cas de maladie de Chagas cliniques ont été diagnostiqués, dont 9 cas autochtones qui ont été contaminés le long des fleuves Maroni et Oyapock [184]. Son agent infectieux (Fig. 23), *T. cruzi*, est principalement transmis par passage transcutané à partir de déjections de punaises hématophages (triatomes) de la famille des Reduviidae (Fig. 24) ou par voie orale, souvent par consommation de jus du palmier wassaï infecté. Bien que la transmission par voie transfusionnelle et congénitale soit rare, elle motive l'exclusion des donneurs de sang guyanais par l’Établissement français du sang. La maladie évolue en deux phases. La phase aiguë de l'infection survient 7 à 15 jours en moyenne après l'inoculation. Elle dure 4 à 8 semaines, est dans 95% des cas pauciou asymptomatique, parfois associée à des signes généraux peu spécifiques (fièvre, adénopathies, hépatosplénomégalie) [326]. Dans moins de la moitié des cas, des signes locaux pathognomoniques et témoins de la transmission vectorielle sont visibles (chagome ou signe de Romaña). Dans 1 à 5% des cas, des complications graves peuvent survenir à type de myocardite, de défaillance cardiaque aiguë droite ou globale parfois très grave [52], plus rarement de méningo-encéphalite. La phase chronique indéterminée, asymptomatique, succède à la phase aiguë et reste stable dans 70% des cas durant toute la vie. Dans les autres cas, elle devient « déterminée » après 10 à 30 ans d’évolution et s'exprime sous forme d'atteintes cardiaques, digestives ou mixtes. En Guyane, entre 2008 et 2018, 8 formes aiguës et 29 formes chroniques (3 réactivations, 18 formes cardiaques, 2 atteintes digestives et 6 formes indéterminées) ont été diagnostiquées [16]. Le diagnostic biologique repose à la fois sur les examens parasitologiques directs ou indirects (dont la PCR *T. cruzi)* et les techniques sérologiques. Le traitement est basé sur deux médicaments aux effets secondaires non négligeables disponibles dans le cadre d'une autorisation temporaire d'utilisation: le benznidazole et le nifurtimox. Le traitement antiparasitaire est indiqué dans les formes aiguës, les réactivations, les formes congénitales, les formes indéterminées et chroniques en l'absence de décompensation cardiaque chez les moins de 50 ans. Cependant l'indication du traitement peut être difficile à établir, en particulier en présence d'une sérologie isolée sans symptomatologie évocatrice [201].

### Infections fongiques

#### Histoplasmose américaine

Morgane Bourne-Watrin, Antoine Adenis

L'histoplasmose américaine est une infection fongique invasive *à Histoplasma capsulatum* var. *capsulatum.* Champignon dimorphique, il se présente sous la forme de filaments dans les sols enrichis en guano des oiseaux et des chiroptères, qui une fois inhalés se transforment en levures dans l'organisme. On décrit deux tableaux cliniques différents selon le statut immunitaire du patient.

**Figure 25:**
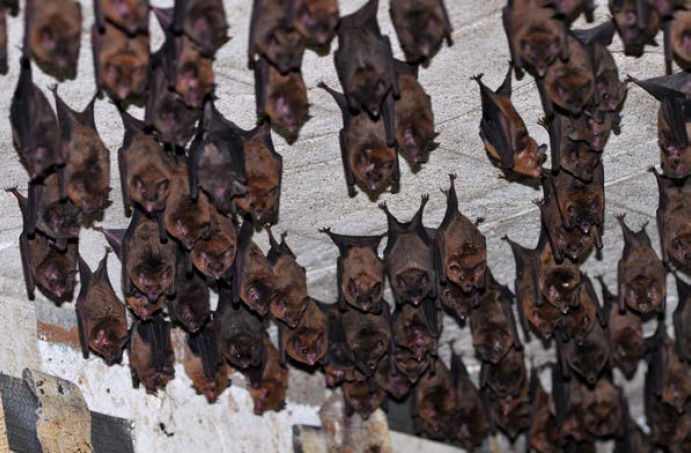
Colonie de chauves-souris dans l'ancienne écloserie de tortues d'Awala-Yalimapo (crédit photo: L. Epelboin) Bat colony in the old turtle hatchery of Awala-Yalimapo (photo credit: L. Epelboin)

**Figure 26:**
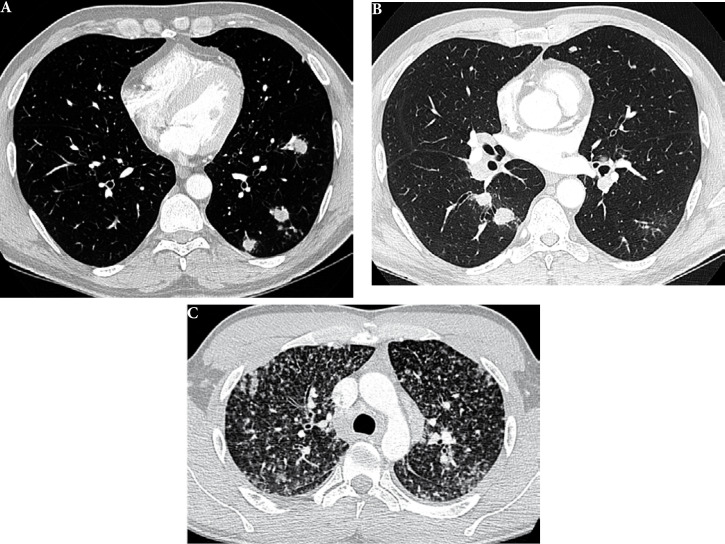
Scanners thoraciques d'histoplasmoses pulmonaires (crédit photo: M. Zappa) Légende: A et B. Scanner thoracique en fenêtre parenchymateuse d'une histoplasmose pulmonaire chez un militaire immunocompétent. Nodules multifocaux bilatéraux du parenchyme pulmonaire. C. Histoplasmose pulmonaire diffuse compliquée de SDRA chez un jeune Guyano-brésilien ayant inhalé une quantité massive de poussière de guano de chauves-souris CTs of pulmonary histoplasmosis (photo credit: M. Zappa) A and B. Parenchymal window chest CT of pulmonary histoplasmosis in an immunocompetent military man. Bilateral multifocal nodules of the lung parenchyma. C. Diffuse pulmonary histoplasmosis complicated by ARDS in a young Guyano-Brazilian man who inhaled massive amounts of bat guano dust

Chez le patient immunocompétent, la symptomatologie est d'autant plus importante que l'inoculum est important. Pour une exposition minime, l'infection sera le plus souvent asymptomatique, source de découvertes fortuites de nodules pulmonaires à l'imagerie et d'examens complémentaires invasifs pour le diagnostic différentiel de la tuberculose et du cancer broncho-pulmonaire [38]. Lors d'une exposition importante (visite de grottes ou bâtiments abandonnés et colonisés par des chiroptères, nettoyage d'enclos à poules avec nombreuses fientes, etc.) (Fig. 25), on observe des tableaux cliniques allant du syndrome pseudo-grippal spontanément résolutif à la pneumopathie aiguë avec SDRA rapidement mortelle en l'absence de traitement antifongique (Fig. 26). L'histoplasmose pulmonaire du sujet immunocompétent est rarement retrouvée en Guyane, peut-être du fait de la rareté des grottes ou d'arbres creux dans lesquels vivraient de très grandes quantités de chauves-souris. Ces infections sont également probablement sous-estimées car peu recherchées face à une symptomatologie peu spécifique, une sévérité rare et le manque d'outil diagnostique fiable dans ce contexte [7].

**Figure 27:**
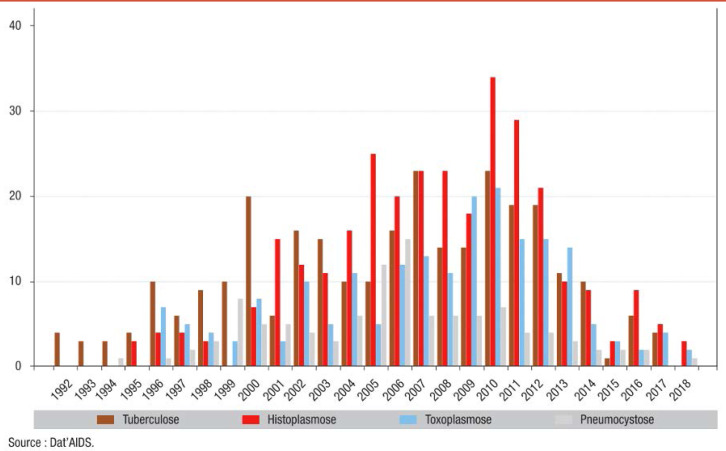
Incidences des 4 principales infections opportunistes en Guyane entre 1992 et 2018 (source: Dat'Aids) Incidence of the 4 main opportunistic infections in French Guiana (source: Dat'Aids)

Chez le patient immunodéprimé, notamment les personnes vivant avec le VIH (PVVIH), l'infection est disséminée dans plus de 90% des cas. Avec une incidence estimée à 1,5 cas pour 100 PVVIH-années, l'histoplasmose est la première cause d'infection opportuniste en Guyane (Fig. 27) avec une létalité précoce désormais faible (< 5%) [257, 262]. La fièvre, la perte de poids et l'altération de l’état général sont quasi constantes (> 80% des cas), associés ou non à des adénopathies, des signes digestifs ou respiratoires [71]. L'atteinte cutanée et muqueuse, amplement rapportée dans les manuels médicaux, correspond probablement à un stade tardif de la maladie, devenu rare en Guyane grâce à la détection précoce des cas [240]. L'histoplasmose est également la première cause de syndrome d'activation macrophagique chez les PVVIH, dont le pronostic est lié à celui de l'infection [264]. Le principal diagnostic différentiel de l'histoplasmose est la tuberculose, deuxième infection opportuniste la plus fréquente en Guyane. Devant les difficultés à faire le diagnostic différentiel au lit du malade, un score clinique d'orientation a été proposé, mais n'exclut pas de multiplier les prélèvements microbiologiques à la recherche de ces 2 agents pathogènes [260]. Ainsi, des prélèvements respiratoires (crachats, crachats induits, tubages et/ou lavages broncho-alvéolaires) doivent être proposés devant toute anomalie pulmonaire, biopsies œsophagiennes et/ou coliques en cas d'endoscopie, le myélogramme avec culture fongique est quasi systématique devant ces tableaux systémiques, et les biopsies ganglionnaires proposées quand un ganglion pathologique est accessible.

**Figure 28:**
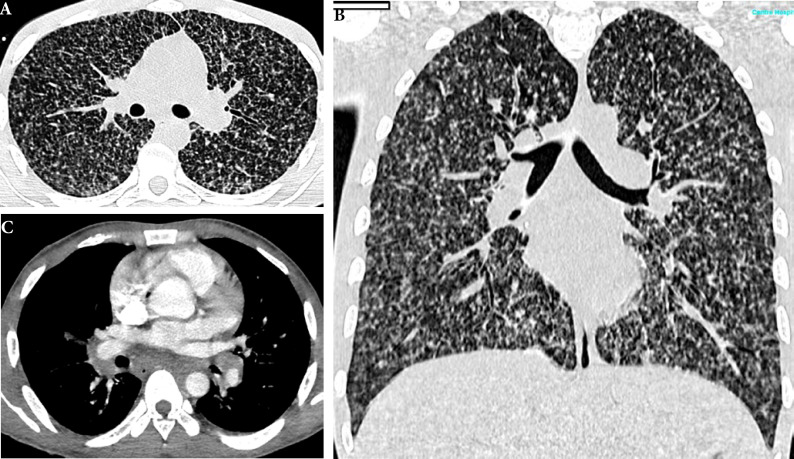
Histoplasmose chez des PVVIH (crédit photo: *M. Zappa)* Légende: A et B. Miliaire hématogène en coupes axiale (A) et coronale (B) sur un scanner thoracique en fenêtre parenchymateuse. C. Adénopathies médiastinales symétriques (hilaires bilatérales et sous carinaire) sur une coupe axiale d'un scanner thoracique en fenêtre médiastinale (crédit photo: M. Zappa) Histoplasmosis in PLWHIV (photo credit: M. Zappa) A and B. Hematogenous miliary in axial (A) and coronal (B) sections of a chest CT scan in parenchymal window. C. Symmetrical mediastinal adenopathies (bilateral hilar and subcarinal) on an axial section of a chest CT scan in mediastinal window

La présentation en imagerie est majoritairement interstitielle nodulaire et/ou micronodulaire, le plus souvent sous forme d'une miliaire hématogène, moins fréquemment sous forme de nodules ou micronodules épars. Dans la moitié des cas environ, les patients présentent des condensations alvéolaires qui sont soit isolées soit le plus souvent associées à des nodules ou micronodules; l'excavation des nodules est possible. Des adénopathies médiastinales sont présentes chez presque les 2/3 des patients; elles sont le plus souvent symétriques, homogènes, non nécrotiques (Fig. 28) [41, 180]. Face à ce profil non spécifique, l'histoplasmose doit être systématiquement recherchée dès lors qu'une tuberculose est évoquée.

Le diagnostic microbiologique de certitude de l'histoplasmose peut être long (médiane de culture 15 jours) et complexe (nécessite un laboratoire P3). Il repose sur l'examen anatomopathologique, l'examen direct et la culture fongique de prélèvements invasifs (biopsies tissulaires, lavage broncho-alvéolaire, myélogramme et hémocultures). La sérologie est peu contributive chez les PVVIH et la biologie moléculaire a peu de place en pratique courante pour l'instant. Des marqueurs antigéniques fongiques indirects sont actuellement utilisés pour l'orientation diagnostique avec de faibles niveaux de preuve, comme l'antigène aspergillaire et le (1-3)-ß-D-glucane [292]. Dans un futur proche, des tests antigéniques spécifiques urinaires seront disponibles sous forme de *Lateral Flow Assays* en cassette, réalisables sous forme de test rapide au lit du malade et avec de très bonnes performances diagnostiques [45].

Le traitement, qui doit le plus souvent être débuté de manière probabiliste au vu des latences diagnostiques, comprend soit l'amphotéricine B liposomale (AmBL) IV jusqu’à amélioration clinique dans les formes sévères, soit l'itraconazole dans les formes modérées à simples. L'itraconazole sera poursuivi en prophylaxie secondaire pour un minimum d'un an. Le posaconazole et l'isavuconazole sont parfois proposés comme alternative. Les cas de co-infections histoplasmose et tuberculose ne sont pas rares et compliquent la prise en charge thérapeutique avec des interactions rifampicine-itraconazole difficiles à gérer, invitant à proposer des schémas thérapeutiques alternatifs [46, 300].

Afin de guider les cliniciens, des recommandations internationales pour le diagnostic et la prise en charge de l'histoplasmose chez les PVVIH ont été publiées en 2020 par l'OMS avec la participation active des experts guyanais [277].

#### Cryptococcose

Loïc Epelboin

La cryptococcose est une infection fongique liée à une levure encapsulée du genre *Cryptococcus neoformans* qui présente 2 caractéristiques principales en Guyane: elle survient de façon non anecdotique chez des patients immunocompétents, et son incidence chez les PVVIH est plus élevée qu'en France hexagonale. Une étude rétrospective conduite entre 1998 et 2008 a permis d'identifier 43 patients atteints de cryptococcose admis dans les hôpitaux de Guyane [85]. Parmi ceux-ci, 14 (32,6%) n’étaient pas infectés par le VIH, dont seulement 2 (4,7%) présentaient une autre cause d'immunodépression. On retrouvait une fréquence élevée de patients positifs pour HTLV-1 (12,1%). *C. neoformans* var. *grubii* comptait pour 77% des cas et était principalement isolé chez les patients infectés par le VIH (13/17), alors que *C. gattii* (22,7%) était strictement isolé chez des patients VIH-négatifs ne présentant aucun facteur de risque apparent. L'incidence moyenne de la cryptococcose a été évaluée à 22,6 cas/million d'habitants/an au cours de la période 1998-2008, soit environ 10 fois plus élevée que dans l'Hexagone [85]. Une étude réalisée au CH de Cayenne sur la période 2011-2018 a identifié 6 patients non immunodéprimés atteints de cryptococcose, tous présentant un tableau neuro-méningé, parmi lesquels 5 étaient de sexe masculin, 5 étaient des adultes, tous d'origine ethnique différente [150]. Le germe incriminé était *C. neoformans* var. *grubii* dans 3 cas et *C. gattii* dans 2 cas. Tous les patients étaient négatifs pour le VIH et l'HTLV. Des recherches approfondies d'anomalies de l'immunité ont été réalisées et des auto-anticorps anti *granulocyte-macrophage colony-stimulating factor* (GM-CSF) ont été identifiés chez 2 patients, et aucun ne présentait de taux détectable d'auto-anticorps dirigés contre l'interféron gamma (IFNγ). Ainsi, la cryptococcose neuro-méningée doit être systématiquement évoquée et recherchée chez tout patient, même en l'absence d'immunodépression, présentant un tableau d'infection neuro-méningée au retour de Guyane voire de fièvre nue prolongée. Le diagnostic biologique et le traitement ne présentent pas de spécificités par rapport aux cas pris en charge dans l'Hexagone.

### Pathologies liées au péril fécal

Lucas Maisonobe, Loïc Epelboin

Si les directives européennes s'appliquent en Guyane en matière de distribution d'eau potable, la Guyane est aussi une terre de migration importante pour les populations des pays voisins (Brésil, Suriname, Haïti, Guyana) qui s'installent souvent en zones urbaines ou périurbaines, dans des habitats spontanés précaires dans des zones non raccordées au réseau d'eau potable. En outre, il existe un problème important d'accès à l'eau potable dans les écarts ou kampoes des communes des fleuves [217]. Ainsi, le taux de non-raccordement y est estimé à 15% contre 1% dans l'Hexagone. Les populations concernées ne disposent pas de système de gestion des eaux usées et ont souvent une méconnaissance du cycle de contamination féco-orale. C'est ainsi le dernier territoire français à avoir enregistré une épidémie de choléra en 1991, à l'exception de Mayotte où ont été rapportés 10 cas entre 1998 et 2000, sans compter le risque théorique d'importation d'Haïti qui a fait face à une épidémie intense [80, 217].

#### Parasitoses digestives

**Figure 29:**
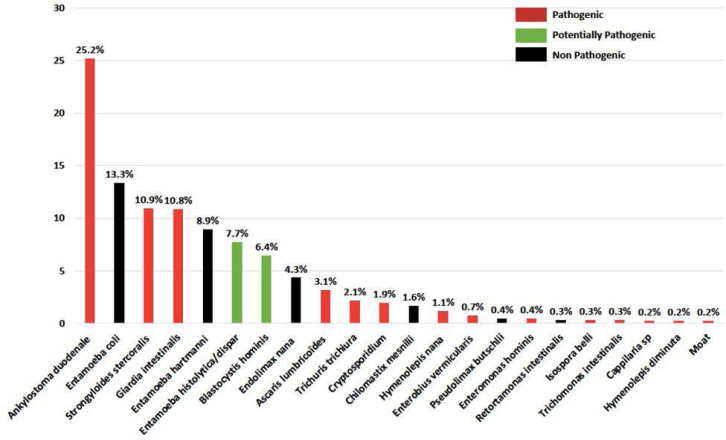
Répartition des espèces de parasites digestifs retrouvés aux laboratoires du CHC et du CHOG entre 2011 et 2016 [2] Distribution of species of digestive parasites found in laboratories of Cayenne general hospital (CHC) and West-Guyanese general hospital (CHOG) between 2011 and 2016 [2]

**Figure 30:**
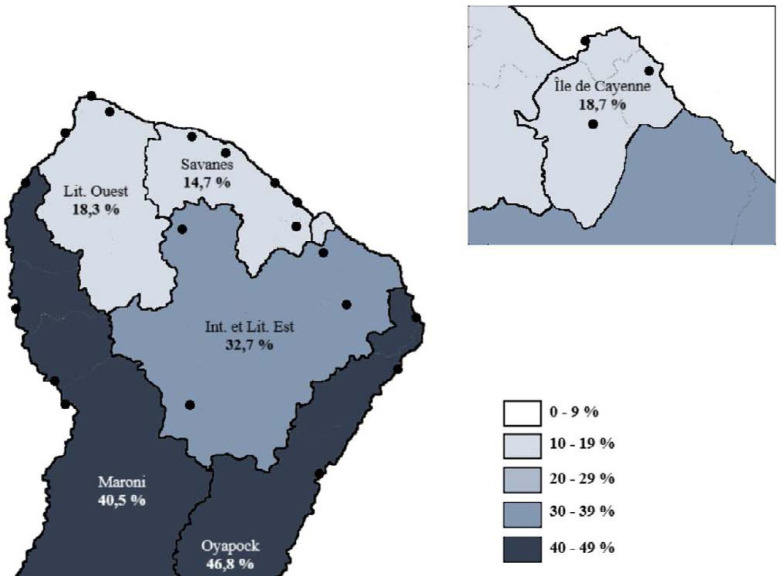
Répartition du taux d'infestation parasitaire (nombre de patients infestés par rapport au nombre de patients testés) entre 2010 et 2019 au laboratoire du CHC [200] Distribution of parasitic infestation rate (number of infested patients over number of patients tested) between 2010 and 2019 at the laboratory of Cayenne general hospital (CHC) [200]

Les parasitoses digestives touchent particulièrement les populations des communes isolées et celles des orpailleurs. Ainsi, une étude publiée récemment a analysé rétrospectivement l'ensemble des prélèvements parasitologiques des selles examinées dans les laboratoires du CHC et du CHOG entre 2011 et 2016 [2]. Au total, 15 220 échantillons de 9 555 patients ont été analysés et 2 916 étaient positifs chez 1 521 patients. Le taux d'infestation (TI) (pourcentage d’échantillons positifs par rapport au nombre total d’échantillons) et l'indice parasitaire (IP) (pourcentage de patients parasités par rapport au nombre total de sujets étudiés) moyens étaient respectivement de 19,2% et 16,0%. L'IP est resté stable entre 2011 (18,2%) et 2016 (18,3%). Les patients étaient principalement des hommes (66,4%), avec un âge médian de 33,0 ans (26,3% < 18 ans) et vivaient principalement sur l’Île de Cayenne (48,2%) et dans l'ouest guyanais (37,4%), en lien avec la sélection des personnes prélevées sur les deux centres hospitaliers. L'ankylostome était le parasite le plus fréquemment retrouvé (25,2%), suivi d’*Entamoeba coli* (13,3%), *Strongyloides stercoralis* (10,9%) et *Giardia intestinalis* (10,8%) (Fig. 29). Parmi les patients infectés, 31,0% présentaient des infections mixtes et 67,5% d'entre eux avaient au moins un parasite pathogène. Les patients âgés de moins de 18 ans présentaient significativement plus de polyparasitisme (30,9%) que de monoparasitisme (24,3%, p < 0,001). *Ancylostoma* sp. et *Strongyloides stercoralis* ont été principalement diagnostiqués pendant la saison des pluies (59,5% et 64,7% respectivement), chez les hommes (78,6% et 81,1% respectivement) et chez les patients âgés de 18 à 65 ans (86,6% et 76,6% respectivement) alors que *Giardia intestinalis* a infecté principalement les enfants de moins de 5 ans (59,5%). Une seconde étude similaire a été menée au sein du laboratoire du CHC sur la période de 2010 à 2019 [200]. Sur les 7 112 patients inclus, 1 655 ont présenté au moins un prélèvement positif soit un IP de 23,3%. Les patients vivant dans les communes du sud de la Guyane (secteurs Maroni et Oyapock) présentaient des IP > 40% (Fig. 30). Les deux nématodes à porte d'entrée cutanée *Ancylostoma* sp. et *Strongyloides stercoralis* étaient les principaux helminthes observés, avec des taux d'infestation respectifs de 7,4% et 3,5%. Les nématodes à porte d'entrée buccale *Ascaris lumbricoides, Trichuris trichiura* et *Enterobius vermicularis*, ainsi que le plathelminthe *Hymenolepis nana* avaient chacun un taux d'infestation inférieur ou égal à 1%. Les deux principales espèces de protozoaires pathogènes observées étaient *Giardia intestinalis* (IP 2,9%) et *Entamoeba histolytica/E. dispar* (IP 2,2%). Enfin, des enquêtes menées auprès de populations d'orpailleurs brésiliens illégaux ont montré un taux de portage d'ankylostome très élevé associé à des carences et des infections multiples [242, 265].

Si le portage chronique d'anguillule ne donne le plus souvent pas de symptômes ou des troubles digestifs modérés, il peut exister un risque d'anguillulose maligne ou un syndrome d'hyperinfection à *Strongyloides stercoralis*, notamment chez les personnes immunodéprimées et chez celles infectées par HTLV-1, ce qui motive l'administration d'un traitement d’éradication systématique par ivermectine [56, 74]. La PCR pourrait occuper une place importante dans le diagnostic de l'anguillulose chez les patients porteurs du HTLV-1, à qui certaines équipes proposent un déparasitage régulier systématique [31]. Chez les patients séronégatifs pour HTLV-1, le principal facteur de risque de cette forme grave est l'immunodépression, et notamment celle induite par une corticothérapie au long cours, retrouvée chez 83,5% des 133 cas étudiés dans 6 réanimations parisiennes [146]. Paradoxalement, cette forme grave de nématodose est rare en Guyane [256]. Ceci pourrait s'expliquer par le déparasitage assez systématique des patients mis sous immunosuppresseurs et corticoïdes au long cours, sans qu'aucune étude n'ait étayé cette affirmation. Néanmoins, une recherche d'anguillule et un déparasitage systématique par ivermectine devront être proposés à l'introduction de tels traitements chez tout patient originaire ou ayant voyagé en Guyane.

#### Salmonelloses typhiques et non typhiques

On a décompté 13 foyers et épidémies de fièvre typhoïde en Guyane entre 1995 et 2007 avec entre 2 et 17 cas, majoritairement sur le Maroni et l'Oyapock (et 2 cas liés en 2006 à Matoury) et le nombre de cas déclaré de fièvre typhoïde s’établit entre 2 et 10 par an depuis 2008 (avec un pic à 19 cas en 2019), ce qui en fait le deuxième territoire français le plus concerné après Mayotte [218, 219]. L'Institut Pasteur à Paris recommande à ce titre une vaccination « en cas de séjour prolongé ou dans des conditions d'hygiène précaires » en Guyane, ce qui paraît largement excessif au vu de la faible efficacité vaccinale (évaluée entre 50 et 65% contre les cas de fièvre typhoïde confirmés par hémocultures) et du risque insignifiant pour un séjour standard sur le littoral, même avec de courtes excursions en forêt [239]. En revanche, elle peut se discuter pour des séjours prolongés en communes isolées du Maroni ou de l'Oyapock. La place d'une éventuelle vaccination pour les populations isolées de Guyane vivant dans de mauvaises conditions d'hygiène reste à déterminer.

Il existe peu de données sur les salmonelles non typhiques en Guyane. Parmi les données récentes, on retrouve un article publié en 2014 sur les salmonelles de reptiles en Guyane et qui fait le parallèle avec les souches humaines [142]. En 2011, le CNR-Salm a reçu 154 isolats humains de *Salmonella enterica* acquises en Guyane dont 139 (90,3%), 14 (9,1%) et 1 (0,6%) appartenaient, respectivement, aux sous-espèces *enterica, houtenae* et *diarizonae.* Les sérotypes fréquemment isolés parmi les sous-espèces *S. enterica enterica* étaient les suivants: Panama (18/139, 12,9%), Oranienburg (11/139, 7,9%), Saintpaul (11/139, 7,9%) et Ouganda (10/139, 7,2%) et la sous-espèce *S. enterica houtenae* sérotype 50:g,z51:- (10/14, 71,4%). On peut noter que *Salmonella* est retrouvée parmi les causes de méningite bactérienne chez l'enfant représentant 4 sur 60 (7%) méningites bactériennes identifiées chez l'enfant dans les 3 hôpitaux de Guyane entre 2000 et 2010, puis 4 méningites à *Salmonella enterica* sérotype Panama identifiées entre 2011 et 2016 à l'hôpital de Cayenne [112, 114]. Les infections à salmonelles sont également fréquemment diagnostiquées chez les personnes souffrant de drépanocytose, maladie génétique fréquente en Guyane. Enfin, une bactériémie à salmonelle non typhique doit faire systématiquement évoquer et rechercher une infection par le VIH.

#### Shigelloses

*Shigella* est une des premières causes de dysenterie dans le monde. La plupart des cas de shigellose concernent des enfants de moins de 5 ans, principalement dans des pays du Sud. En Guyane c'est une cause fréquente d'infection digestive chez l'enfant. Ainsi, une étude rétrospective observationnelle a été réalisée au CHOG, identifiant 213 cas de shigellose entre 2000 et 2012 diagnostiqués sur des selles d'enfants de moins de 5 ans [282]. *Shigella* était la seconde bactérie isolée en fréquence dans les selles (taux de positivité de 210 souches/3366 coprocultures réalisées, soit 6%) derrière les *E. coli* entéropathogènes (280/3366 soit 8%). Les autres genres bactériens isolés étaient *Salmonella* (120/3366 soit 4%), *Campylobacter* (105/3366 soit 3%) et *Yersinia* (2/3366 soit 0,6%). Parmi les 209 souches identifiées, 161 (77%) appartenaient à l'espèce *Shigella flexneri* et 48 (23%) à l'espèce *Shigella sonnei*. Les souches de *S. sonnei* étaient le plus souvent résistantes au cotrimoxazole et sensible à l'amoxicilline, à l'inverse de *S. flexneri*, mais toutes les souches étaient sensibles aux fluoroquinolones et aux C3G (céphalosporines de 3^e^ génération). Une autre étude réalisée au CHOG a montré un lien entre les 34 femmes enceintes infectées par *Shigella* entre 2000 et 2014 et des symptômes de menace d'accouchement prématuré [281]. Enfin, les shigelloses touchent également les adultes vivant dans des conditions précaires comme cela a été rapporté chez des orpailleurs illégaux œuvrant en forêt [242].

#### Cryptosporidioses

La cryptosporidiose humaine est causée par *Cryptosporidium* spp., un parasite protozoaire qui peut infecter les humains et les animaux. En Amérique du Sud, la cryptosporidiose est fréquente chez les enfants immunocompétents et se manifeste par des épisodes à répétition associant généralement une diarrhée profuse d’évolution spontanément favorable et plus rarement des symptômes chroniques ou systémiques chez les patients immunocompétents. Le traitement est généralement symptomatique, mais le nitazoxanide a démontré son activité contre *Cryptosporidium* et constitue un traitement efficace dans certaines situations. La maladie humaine est principalement due à *Cryptosporidium parvum* qui infecte à la fois les humains et les ruminants, et *Cryptosporidium hominis* qui se limite presque exclusivement à l'Humain. De nombreux cas sont régulièrement diagnostiqués chez de jeunes enfants amérindiens wayanas et businenges aluku sur le haut Maroni [246]. Plusieurs cas groupés ont également été rapportés ces dernières années en Guyane, notamment à Maripasoula, avec des tableaux de gastroentérite aiguë à *Cryptosporidium hominis* à la fois chez de jeunes enfants, mais aussi chez des militaires adultes [231].

#### Hépatite A

Il n'existe pas d'estimation récente de la séroprévalence en Guyane. La dernière étude remonte à 1997 et retrouvait une séroprévalence à 68,7% d'IgG dans la population globale avec des taux allant de 84,2% chez les personnes d'origine hmong jusqu’à 52,9% pour les personnes dites caucasiennes [353]. À titre de comparaison, une étude menée en 1999 auprès des jeunes recrues au Service national français retrouvait une prévalence de 7,65% chez ceux n'ayant jamais séjourné dans des pays du Sud et en outre-mer alors qu'elle était de 46% chez les jeunes originaires des DOM-TOM et de 28% chez ceux ayant déjà séjourné en outre-mer [188]. Plus récemment, une étude brésilienne estimait la séroprévalence à 67,5% chez les jeunes de 10 à 19 ans vivant dans le nord du pays [288]. En Haïti, une étude récente chez les femmes enceintes fait état d'une séroprévalence de 96,8% Elle était de 94,9% chez les immigrés originaires d'Haïti résidant au centre du Brésil [360].

#### Hépatite E

Il n'existe aucune donnée récente concernant la séroprévalence de l'hépatite E en Guyane. Une étude de 1997 retrouvait une séroprévalence de 6,4% d'IgG anti-VHE, avec une différence significative en fonction de l'origine ethnique des personnes (de 14,6% dans les populations hmong et chinoises à 0,6% dans les populations businenges) [353]. Ces taux sont bien inférieurs à ceux retrouvés chez les donneurs de sang dans les dernières études en France hexagonale (24% en moyenne et jusqu’à 40% dans le sud de la France) [289]. Concernant les pays d'Amérique du Sud et notamment les pays limitrophes, les différentes études révèlent que le génotype 3 est le principal génotype circulant dans cette région chez l’être humain et chez l'animal. Le génotype 1 n'a été retrouvé qu'en Uruguay et au Venezuela dans des cas d'hépatite aiguë. La séroprévalence au Brésil dans la population générale est actuellement estimée autour de 4,2%, inférieure à celle retrouvée dans les pays européens ou aux États-Unis (9,0%) [172, 295]. Il n'existe pas de données concernant le Suriname. Concernant Haïti, la séroprévalence a été estimée à 10,3% chez les femmes enceintes et les personnes originaires d'Haïti représentaient l'intégralité des cas séropositifs dans une étude réalisée auprès d'immigrés du centre du Brésil, y compris des enfants nés au Brésil de parents haïtiens (2 de ces cas étaient aussi positifs en ARN VHE, traduisant une infection active) [342, 360]. Ainsi, face à cette lacune majeure dans la connaissance sur les hépatites virales à transmission féco-orale A et E, il est impératif de mettre en place des études visant à évaluer leur incidence et leur séroprévalence en Guyane.

## Agents Pathogènes Rares, Émergents Et/Ou Absents De Guyane, Mais À Potentiel D’Émergence

Loïc Epelboin

### Bactéries

Ces dernières années ont été l'occasion de voir identifiés des agents pathogènes jusque-là non rapportés en Guyane. Ainsi une nouvelle espèce d’*Anaplasma*, nommée pour l'occasion *Anaplasma sparouinii*, a été identifiée chez un orpailleur brésilien. Son nom a été choisi en rapport avec le site d'orpaillage où travaillait le patient [108]. Alors que ce genre n'avait jamais été identifié en Guyane, trois cas de brucellose ont été diagnostiqués entre 2017 et 2020, également chez des orpailleurs brésiliens. Si le premier, probablement lié à un élevage de porcs au Brésil, est à *Brucella suis*, les deux cas suivants ont été l'occasion de la découverte d'une nouvelle espèce dont le nom proposé est *B. amazoniensis*, possiblement liée à une acquisition en forêt auprès de suidés sauvages [3, 230]. La mélioïdose, infection bactérienne liée à *Burkholderia pseudomallei*, est une zoonose à transmission hydrique rapportée de plus en plus fréquemment au Brésil ainsi que dans les Antilles françaises [285]. Aucune suspicion n'a jusqu'alors été confirmée en Guyane, mais sa distribution cosmopolite laisse cette possibilité ouverte [286]. Enfin, plusieurs infections à *Chromobacterium violaceum* ont été décrites ces dernières années en Guyane [Maisonobe, données non publiées].

### Parasites

Suite à l'identification de plusieurs cas d'angiostrongylose aux Antilles et à l’émergence de ces pathologies au Brésil, un premier cas d'angiostrongylose nerveuse à *Angiostrongylus cantonensis* et un cas d'angiostrongylose abdominale à *Angiostrongylus costaricencis* ont été récemment rapportés [86, 91]. Une étude menée en Guyane parallèlement aux Antilles a retrouvé une prévalence élevée du portage d'A. *cantonensis* chez l'espèce invasive de l'escargot *Achatina immaculata* (achatine immaculée) et non pas *Achatina fulica* (escargot géant africain) comme ailleurs en Amérique latine avec 18,6% des gastéropodes porteurs de parasites [78]. Quelques cas d’échinococcose alvéolaire à *Echinococcus vogeli* ont été rapportés en Guyane et au Suriname, espèce majoritairement retrouvée dans la zone tropicale de l'Amérique latine [20, 84, 193].

Certaines pathologies parasitaires ne sont pas considérées comme actuellement existantes en Guyane. Ainsi la schistosomiase, pourtant présente au Suriname, n'est pas diagnostiquée en Guyane bien que le gastéropode hôte intermédiaire y semble présent [379]. Existant toujours de façon localisée au Guyana et au Brésil, la filariose lymphatique n'est plus signalée en Guyane [134]. Cette pathologie doit cependant être évoquée en cas d'hyperéosinophilie ou de symptomatologie compatible chez les patients originaires d'Haïti ou du Brésil, où la filariose à *Wuchereria bancrofti* sévit encore. Bien que présente dans de nombreux pays d'Amérique latine, d'Amérique tropicale ainsi qu’à Haïti et Cuba dans les Caraïbes, la cysticercose, forme tissulaire de l'infection à *Taenia solium*, n'est pas rapportée localement en Guyane. En revanche, il faut savoir la chercher en cas de tableau neurologique spécifique chez des patients originaires des zones d'endémie [212].

### Champignons

La paracoccidioïdomycose est une mycose profonde due à un champignon dimorphique appartenant à l'espèce *Paracoccidioides brasiliensis* avec une atteinte le plus souvent cutanéo-muqueuse, ganglionnaire et abdominale. La répartition géographique de cette affection est constituée par le continent sud-américain, notamment le Brésil, la Colombie et le Venezuela. Pour des raisons mystérieuses, les cas rapportés en Guyane sont très rares et surviennent chez des ressortissants brésiliens. Une co-infection avec une tuberculose pulmonaire ainsi que des localisations ORL ont été rapportées [155, 327, 336]. Les formes invasives d'infection à *Aspergillus* sp. sont rarement rapportées en Guyane, probablement du fait de la rareté des patients présentant les facteurs de risque habituels, notamment les patients présentant des neutropénies prolongées et les patients post-greffe de moelle, qui sont généralement pris en charge dans l'Hexagone. Un aspergillome est parfois diagnostiqué chez des patients présentant des cavités pulmonaires séquellaires d'infections mycobactériennes.

### Virus

Parmi les arbovirus, certains n'ont jamais été rapportés à ce jour, tel le virus West Nile pourtant observé de façon anecdotique chez des oiseaux et des chevaux au Brésil et possiblement chez l’être humain au vu des études de séroprévalence [70, 220]. Il est possible que, faute de recherche systématique chez l’être humain et faute de surveillance sérologique chez les chevaux et les oiseaux migrateurs, ce virus soit passé jusqu’à présent « sous les radars » en Guyane. D'autres arbovirus décrits en Amérique latine, comme le virus de l'encéphalite de Saint-Louis, et différents virus de l'encéphalite équine (à l'exception du virus Tonate) n'ont jamais été identifiés en Guyane. Certains virus observés dans les années 1970, chez des moustiques et/ou des oiseaux et même chez des humains, n'ont plus été retrouvés depuis cette époque: virus Mucambo (isolé néanmoins en 1973 chez deux laborantins qui avaient manipulé la souche de référence), virus Pixuna, virus Una, virus Aura, virus Ilheus (isolé chez un malade fébrile dans les années 1970), virus Murutucu, virus Guama et virus Cabassou [94, 95]. Il reste à attendre pour savoir si l'un d'entre eux sera à nouveau observé, ce qui semble être le cas au vu de nouvelles investigations menées actuellement au CNR des arbovirus de Cayenne.

## Maladies Infectieuses Et Pédiatrie

Lindsay Osei, Nicolas Vignier, Narcisse Elenga

Le poids des maladies infectieuses chez l'enfant est particulièrement élevé en Guyane puisque cette seule cause représentait 24,5% des décès d'enfants de moins de 15 ans entre 2007 et 2016. Les maladies infectieuses sont la cause la plus fréquente de consultation et d'hospitalisation pour les enfants des communes isolées [301]. De plus, le taux de mortalité des enfants de moins de 15 ans en Guyane était le plus élevé de tous les départements français [280]. Les enjeux débutent dès la naissance, la Guyane accueillant une démographie dynamique (taux de natalité de 27,5/1000 habitants *vs* 10,7 dans l'Hexagone) avec cependant des taux de prématurité et de mortinatalité deux fois plus élevés (13,5% *vs* 7% et 18,5/1000 naissances vs 8,5/1000 respectivement).

Comme en témoigne le calendrier de vaccination spécifique en vigueur (vaccin obligatoire contre le BCG avant la sortie de la maternité et vaccination anti-amarile à l’âge de 12 mois, dose précoce contre l'hépatite B à la naissance) [239], les maladies infectieuses constituent une préoccupation majeure pour la santé pédiatrique en Guyane. La couverture vaccinale est sous-optimale pour certaines maladies, en particulier dans les communes éloignées et dans les zones rurales avec certains enfants vivant dans des conditions extrêmement précaires (absence d'eau potable, insécurité alimentaire, dénutrition). Entre 2000 et 2010, *Streptococcus pneumoniae* et *Haemophilus influenzae* étaient les deux bactéries les plus fréquemment isolées dans le liquide céphalorachidien des enfants hospitalisés pour méningite bactérienne [114]. La majorité des enfants qui ont souffert de ces types de méningites bactériennes n'avaient pas été vaccinés contre ces bactéries, avec seulement 2 enfants sur 24 qui étaient vaccinés contre *S. pneumoniae* et trois enfants sur 14 contre *Haemophilus influenzae.*

L’épidémie de Covid-19, qui a révélé une attitude négative à l’égard de sa vaccination tant pour les personnels de santé que pour le reste de la population en Guyane, soulève des inquiétudes quant au risque de dégradation de l'adhésion aux vaccinations infantiles dans un contexte où il existe déjà des barrières structurelles à l'accès à la vaccination [123, 373]. C'est aussi dans ce contexte pandémique que la Guyane a vu les inégalités sociales s'aggraver avec notamment des niveaux d'insécurité alimentaire alarmants pour les familles. Cette carence d'accès en qualité et quantité ne doit pas faire oublier la fréquence inquiétante du surpoids et de l'obésité pédiatriques en Guyane. D'autres points peuvent être soulignés comme la fréquence des cas de gale observés dans les communes de l'intérieur et dans les zones d'habitat informel, et la fréquence des caries à confronter à un accès beaucoup plus faible au suivi odontologique que dans l'Hexagone [97].

## Dermatologie Tropicale De Guyane

### Lèpre

Roxane Schaub, Romain Blaizot

**Figure 31:**
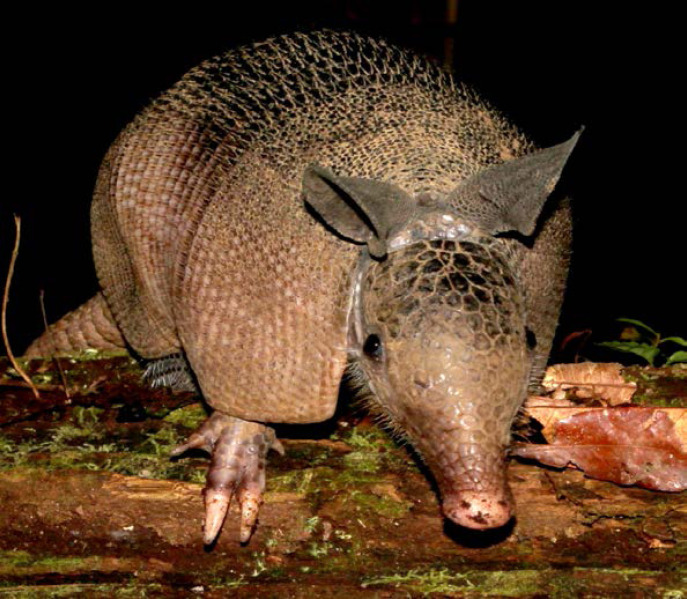
Tatou à neuf bandes *(Dasypus novemcinctus)* (crédit photo: S. Sant) *Nine-banded armadillo* (Dasypus novemcinctus) *(photo credit: S. Sant)*

**Figure 32:**
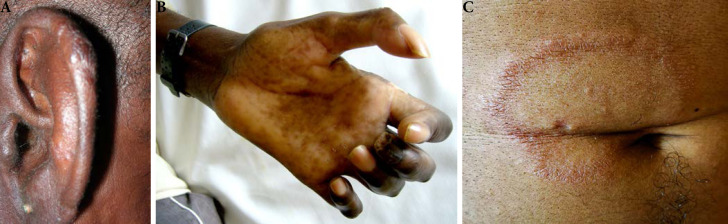
Lèpre Légende: A. Léprome dermique de l'oreille (crédit photo: J. Dufour); B. Griffe cubitale (crédit photo: L. Epelboin); C. Lésion hypochrome péri-ombilicale (crédit photo: J. Destoop) Leprosy A. Dermal leproma of the ear (photo credit: J. Dufour); B. Ulnar claw (photo credit: L. Epelboin); C. Periumbilical hypochromic lesion (photo credit: J. Destoop)

La lèpre ou maladie de Hansen est une infection cutanée chronique due à *Mycobacterium leprae* ou *Mycobacterium lepromatosis.* Le mode de transmission est principalement interhumain, en particulier si le patient est multibacillaire, mais la transmission du tatou (Fig. 31) à l’être humain a été mise en évidence aux Etats-Unis et au Brésil [75, 341, 367]]. La significativité de cette transmission zoonotique en termes de santé publique est encore à déterminer. Avec environ 10 nouveaux cas par an, l'incidence moyenne de la lèpre en Guyane était de 5,3 cas pour 100 000 habitants/an entre 1997 et 2021, dépassant le seuil OMS de problème de santé publique (1/100 000) dans les années 2010 avant de diminuer [98, 151, 152]. Bien qu'il y ait des cas autochtones témoignant d'une transmission locale, plus de la moitié des patients sont originaires du Brésil, dont beaucoup travaillent dans l'orpaillage illégal. La prédominance des formes multibacillaires [152] et la présence en Guyane du tatou, gibier particulièrement apprécié qui est infecté par *M. leprae*, témoignent de la persistance du risque de transmission de la maladie sur le territoire [337]. La lèpre est donc un problème de santé publique en Guyane, notamment chez les migrants originaires du Brésil, et doit être évoquée chez tout patient présentant des lésions évocatrices (Fig. 32). Le traitement est rendu complexe par le suivi chaotique des patients, notamment les orpailleurs brésiliens, du fait de leur situation irrégulière et de leurs conditions de vie en camp isolé. Dans cette population, de nombreuses interruptions rallongent la durée totale du traitement, laquelle est dans 80% des cas supérieure à la durée OMS théorique de 6 ou 12 mois selon la forme [Petiot *et al.*, en cours de publication]. Le traitement repose sur une polychimiothérapie composée de trois médicaments pour éviter l'apparition de résistance: rifampicine, dapsone (après élimination d'un déficit en G6PD) et clofazimine (https://apps.who.int/iris/bitstream/handle/10665/274127/9789290226963-fre.pdf). En cas de déficit en G6PD, la dapsone pourra être remplacée par de la clarythromycine. Ce traitement sera instauré pour une durée de 6 mois en cas de forme paucibacillaire et 12 mois en cas de forme multibacillaire. Les états réactionnels représentent un autre enjeu thérapeutique, car souvent à l'origine d'une corticodépendance. En Guyane, les moyens du système de santé permettent de tester des molécules coûteuses comme l'infliximab, avec des résultats prometteurs pour casser les réactions immunes et sevrer les patients en corticoïdes [368].

### Ulcère de Buruli

Justin Destoop, Maylis Douine

**Figure 33:**
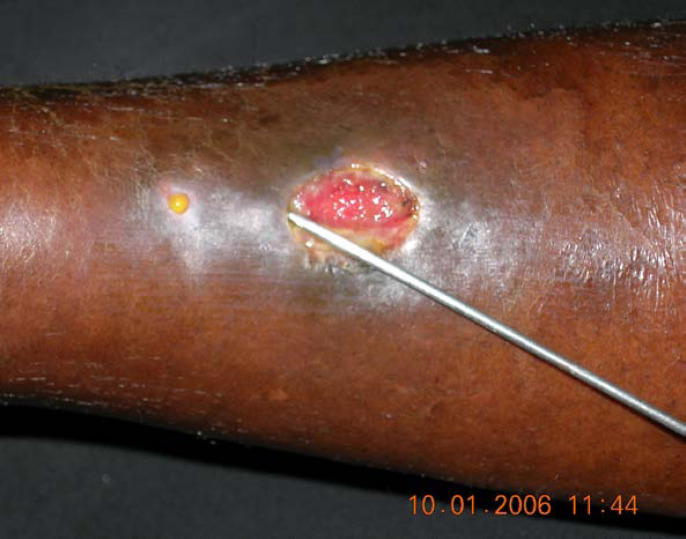
Ulcère de Buruli de la jambe, Guyane (crédit photo: J. Dufour) Buruli ulcer of the leg, French Guiana (photo credit: J. Dufour)

*Mycobacterium ulcerans* est responsable, chez l’être humain, d'une infection cutanée sévère appelée ulcère de Buruli (UB) (Fig. 33). Elle fait partie des 17 maladies tropicales négligées selon les critères de l'OMS [304]. Endémique dans les régions d'Asie du Sud-Est, d'Australie et d'Afrique centrale et de l'Ouest, elle est retrouvée dans une moindre mesure en Amérique latine. C'est en Guyane qu'on observe l'incidence apparente la plus élevée du continent sud-américain (3,49 cas /100 000 habitants/an (2,83 – 4,16) sur la période de 1999-2013) [99]. Une étude cas-contrôle réalisée en Guyane entre 2002 et 2004 a identifié les facteurs de risque d'UB suivants: habiter ou travailler à proximité d'un point d'eau douce (marais, rivières, zones inondables), pratiquer des activités de loisirs, chasser ou pêcher à proximité d'une rivière [115]. Ce lien étroit avec l'exposition environnementale a été corroboré par la découverte pour la première fois en Amérique du Sud d'ADN de *M. ulcerans* dans 5 prélèvements d'environnement dans les communes de Tonate et Sinnamary [241]. Depuis, de nouvelles études environnementales ont montré une présence plus large d'ADN dans l'environnement aquatique, principalement en milieu rural, mais aussi dans les environnements urbains perturbés, et favorisée par la saison des pluies [64]. L'UB se manifeste classiquement sous forme d'ulcération unique touchant préférentiellement les membres inférieurs des adultes [241]. La présence d'un décollement des berges de l'ulcère permet d'orienter le diagnostic d'UB par rapport aux autres causes d'ulcères tropicaux (leishmaniose, ecthyma). L'histologie, la recherche de BAAR, la culture de mycobactéries et la PCR de *M. ulcerans* prélevé sur des biopsies des berges de l'ulcère permettent de confirmer le diagnostic. Le traitement actuellement recommandé par l'OMS repose sur l'association de rifampicine (10 mg/kg/j) et de clarithromycine (7,5 mg/kg 2x/j) pour une durée de 8 à 12 semaines [278]. L'aggravation initiale après l'introduction du traitement, appelée réaction paradoxale, est fréquente et ne doit pas faire arrêter le traitement précocement [328]. Le patient doit en être prévenu à l'avance.

### Diphtérie cutanée

Mélanie Gaillet

**Figure 34:**
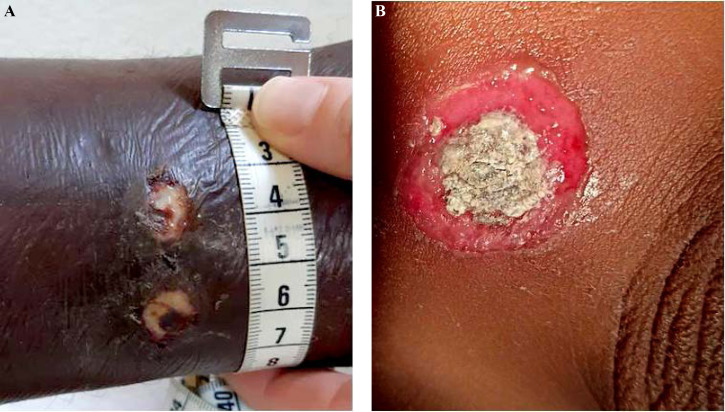
Diphtérie cutanée Légende: A. Patient du Maroni de 46 ans présentant depuis 5 jours des lésions multiples des deux jambes ulcérées, fibrineuses et inflammatoires de 1 à 1,5 cm de diamètre; B. Lésions cutanées multiples et diffuses sur l'ensemble du corps (oreille, visage, aine) associées à des adénopathies sensibles, apparues depuis 3 jours chez un enfant du Maroni de 3 ans (crédit photos: M. Gaillet) Cutaneous diphtheria A. 46-year-old patient from Maroni with multiple ulcerated, fibrinous and inflammatory lesions on both legs, 1 to 1.5 cm in diameter, since 5 days; B. Multiple diffuse skin lesions all over the body (ear, face, groin) associated with tender adenopathies, which appeared 3 days before in a 3-year-old child from Maroni (photo credits: M. Gaillet)

Alors qu'en Guyane, un seul cas d'infection à corynébactéries avait été rapporté depuis les années 2000 [339], 62 cas ont été diagnostiqués depuis 2016 touchant des personnes âgées de 29,1 ans en moyenne, avec un sex-ratio H/F de 1,7 [Gaillet, données non publiées]. Les estimations d'incidence augmentent chaque année: on comptait 2 cas en 2016 et en 2017, 9 en 2018, pour atteindre 19 cas en 2021. Trois espèces appartenant au complexe *diphtheriae* sont pathogènes pour l’être humain: *Corynebacterium diphtheriae* (98% des cas guyanais) dont le réservoir est humain, *C. ulcerans* (1 cas guyanais) et *C. pseudotuberculosis*, ces deux dernières étant des zoonoses [363]. Les infections cutanées surviennent le plus souvent sur une plaie préexistante et se repèrent par la présence de fausses membranes grises. Elles ne doivent pas être confondues avec les colonisations de plaies par corynébactéries, caractérisées par un environnement polymicrobien et l'absence de souche toxinogène. Dans ce dernier cas, le rôle pathogène spécifique de la corynébactérie n'est pas établi (Fig. 34). Le diagnostic n'est pas aisé du fait du caractère aspécifique des lésions (ulcérations plus ou moins creusantes, extensives, multiples et inflammatoires) et des diagnostics différentiels pouvant être évoqués, en particulier en milieu tropical tels qu'impétigo, ecthyma et leishmaniose [215]. La bactérie est généralement isolée à partir d’écouvillonnages réalisés au niveau de la lésion, ou à partir d'hémocultures. Les milieux ensemencés sont aspécifiques. L'identification se fait par spectrométrie de masse, complétée par l’étude de la toxinogénèse de la souche (recherche du gène *tox* par PCR). En cas de positivité, la production de la toxine est recherchée et la souche typée. L'antibiogramme est systématique du fait de l’évolution de l'antibiorésistancerécenteetenaccordavecles recommandations européennes [347]. Selon le tableau clinique, l'espèce, la présence de la toxine ou non, la prise en charge implique un isolement respiratoire (précautions complémentaires type gouttelettes, c'est-à-dire masque chirurgical pour patient et soignants) ou de la plaie (pansement couvrant la plaie), une antibiothérapie, la vérification et la mise à jour vaccinale, le dépistage et la prise en charge des cas contacts [346]. La présence de la toxine impose de faire une déclaration obligatoire et une sérothérapie [158, 160]. La vaccination généralisée contre la diphtérie a permis de limiter le nombre de cas autochtones *tox+* dans l'Hexagone. Alors qu'aucune diphtérie *tox+* n'avait été diagnostiquée en Guyane jusqu'en 2020, 4 infections cutanées *tox+* ont été récemment diagnostiquées sur le territoire, modifiant l’épidémiologie locale.

### Dermohypodermites bactériennes aiguës

Justin Destoop, Pierre Couppié

**Figure 35:**
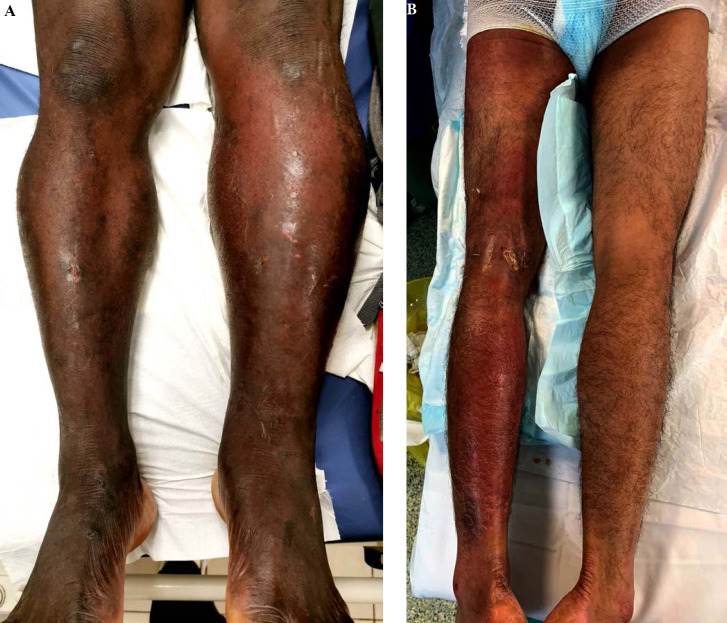
Dermohypodermites bactériennes Légende: A. DHBNN simple de la jambe gauche sur peau noire; B. Fasciite nécrosante du membre inférieur gauche à Aeromonas hydrophila suite à une piqûre de raie au niveau du creux poplité (crédit photos: J. Destoop) Bacterial dermohypodermatitis A. Simple non-necrotizing bacterial dermohypodermatitis of the left leg on black skin; B. Necrotizing fasciitis of the left lower limb with Aeromonas hydrophila following a stingray puncture in the popliteal fossa (photo credits: J. Destoop)

Les dermohypodermites bactériennes non nécrosantes (DHBNN) sont des infections fréquentes. Leur taux d'incidence est plus élevé dans les régions tropicales [216]. En Guyane, le profil clinique semble similaire aux régions tempérées, à l'exception d'une prédominance masculine [92]. En plus des facteurs de risque classiques de DHBNN des membres inférieurs, l'utilisation de produits dépigmentants à base de dermocorticoïdes est un facteur de risque à rechercher [296]. Les DHBNN se manifestent par l'apparition brutale d'un placard érythémateux associé à des signes généraux (fièvre, frissons). L'absence d’érythème sur peau noire peut rendre plus difficile le diagnostic (Fig. 35A). Les signes de gravité orientant vers une forme nécrosante sont à rechercher impérativement: hypoesthésie, hyperalgie, cyanose, nécrose, crépitations sous-cutanées. Les principaux germes impliqués dans les DHBNN sont les streptocoques ß-hémolytiques (principalement du groupe A) et le *Staphylococcus aureus* [92]. Cependant, le monde tropical expose fréquemment à des germes plus « atypiques » (par ex: *Aeromonas hydrophila* en cas de morsures de serpent ou d'exposition à l'eau douce (Fig. 35B), *Vibrio vulnificus* en cas d'exposition à l'eau de mer [166, 296]). Dans ces situations, les prélèvements locaux de la porte d'entrée s'avèrent utiles pour orienter le diagnostic bactériologique et guider l'antibiothérapie [93]. La prise en charge repose dans les formes simples sur une antibiothérapie probabiliste anti-streptococcique (+/- anti-staphylococcique) mais des situations « atypiques » doivent faire réfléchir le prescripteur à un élargissement de l'antibiothérapie (par exemple, l'antibiothérapie recommandée par l'Infectious Diseases Society of America pour les infections à *A. hydrophila* est l'association doxycycline 100 mg/12 h plus ciprofloxacine 500 mg/12 h ou ceftriaxone 1-2 g/24 h) [349]. La prise en charge des formes nécrosantes repose sur une prise en charge médico-chirurgicale (parage/nécrosectomie).

### Leishmaniose cutanée

Romain Blaizot

La Guyane est une zone d'endémie de la leishmaniose cutanée (LC), dont la transmission après piqûre de phlébotome est directement liée à l'existence du milieu forestier amazonien. Environ 200 cas sont rapportés chaque année, avec des variations importantes selon les années, possiblement du fait de facteurs climatiques encore imparfaitement compris [28, 210, 344]. Cinq espèces ont été décrites en Guyane comme étant à l'origine de la maladie chez l’être humain.

**Figure 36:**
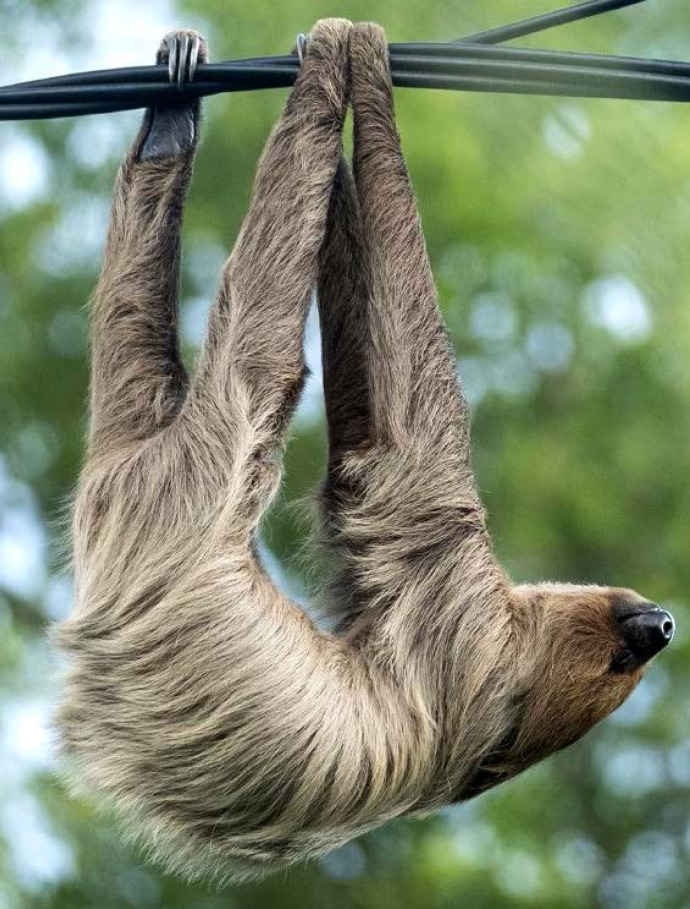
Paresseux à deux doigts ou didactyle *(Choloepus didactylus)* (crédit photo: N. Defaux) *Linnaeus's two-toed sloth* (Choloepus didactylus) *(photo credit: N. Defaux)*

**Figure 37:**
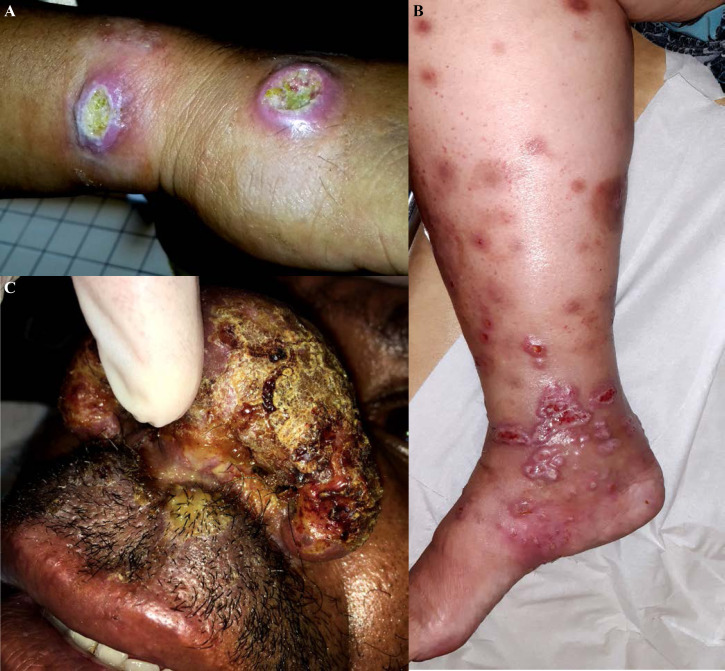
Leishmaniose Légende: A. Leishmaniose typique avec ulcères à bords surélevés avec peau saine entre les deux lésions; B Leishmaniose à L. guyanensis, multiples lésions entraînant un œdème de membre et un handicap à la marche chez une femme de 50 ans au retour de Saül; C. Leishmaniose muqueuse avec atteinte nasale chez un orpailleur, évoluant depuis plusieurs mois, espèce non identifiée (crédit photos: R. Blaizot) Leishmaniasis A. Typical leishmaniasis with ulcers with raised edges and healthy skin between the two lesions; B. L. guyanensis leishmaniasis, multiple lesions leading to limb edema and walking disability in a 50-year-old woman returning from Saül; C. Mucosal leishmaniasis with nasal involvement in a gold digger, evolving for several months, species not identified (photo credits: R.Blaizot)

*Leishmania guyanensis* est nettement prédominante (˜ 85%), suivie de *L. braziliensis* (˜ 10%). Les cas liés à *L. amazonensis, L. lansoni* et *L. naiffi* sont rapportés de façon plus anecdotique [105, 210]. Le cycle parasitaire de *L. guyanensis* se déroule dans la canopée avec le phlébotome arboricole *Lutzomyia umbratilis* comme vecteur, qui pique dès la tombée du jour et durant toute la nuit. Le paresseux didactyle (*Choloepus didactylus*) en est le principal réservoir, bien que ce phlébotome puisse s'adapter, notamment en milieux anthropisés, pour se nourrir sur des rongeurs [321] (Fig. 36). Le contact entre l’être humain et le vecteur se fait généralement à l'occasion d'une anthropisation du milieu sauvage, notamment la déforestation. Les patients se contaminent particulièrement au début de la saison des pluies [27, 319]. En Guyane, les formes humides sont majoritaires (80%) à type d'ulcère typiquement à bords surélevés et accompagnés d'une lymphangite froide [210] (Fig. 37). Des formes sèches à type de croûte, de nodules ou de papules sont également retrouvées. Les formes muqueuses représentent 1% des cas seulement, paradoxalement plus souvent causées par *L. guyanensis*, du fait de la fréquence de cette espèce. La LC touche les hommes dans deux tiers des cas. Le délai d’évolution de la lésion est généralement d'un mois avant la consultation. La leishmaniose viscérale n'est pas présente en Guyane malgré son existence au Brésil, probablement du fait d'une absence de vecteur compatible. Seuls 2 cas de leishmaniose viscérale à *L. infantum* ont été rapportés ces 15 dernières années. Il s'agissait probablement de cas importés chez des patients orpailleurs brésiliens vivant en forêt infectés par le VIH [87]. Le diagnostic parasitologique de la LC est fait à l'aide du frottis coloré au MGG, de la culture sur biopsie cutanée ou d'une PCR sur écouvillon en coton. Cette dernière technique est la plus sensible et permet un diagnostic d'espèce rapide [32]. La culture suivie d'une spectrométrie de masse peut également permettre une identification, mais avec un délai plus long [195]. En cas d'identification de *L. guyanensis*, le traitement curatif consiste en une seule injection intramusculaire de 7 mg/kg d'iséthionate de pentamidine (Pentacarinat^©^), avec une efficacité de plus de 90%. En cas d'identification de *L. braziliensis*, le traitement repose sur l'amphotéricine B liposomale (Ambisome^©^) 4 mg/kg/j pendant 5 jours [340]. En deuxième intention, la miltéfosine (Impavido^©^) à raison de 50 mg trois fois par jour, est efficace et présente l'avantage d'un traitement à prise orale. Les techniques de traitement topique ou local, utilisées dans « l'Ancien Monde » ou dans les pays d'Amérique du Sud à ressources limitées, n'ont pas lieu d’être utilisées dans le traitement des leishmanioses guyanaises, du fait du potentiel de dissémination cutanée et muqueuse et de la possibilité dans le contexte guyanais d'utiliser des traitements systémiques difficilement accessibles dans le reste de l'Amérique latine. La prophylaxie est basée sur le port de vêtements longs, l'utilisation de répulsifs et de moustiquaires imprégnées à fines mailles, et si possible la réduction des déplacements en forêt pendant la période de transmission maximale (reprise et premiers mois de la saison des pluies de décembre à mars). Cette pathologie est bien appréhendée par les populations guyanaises en général, et la multiplicité des remèdes traditionnels en témoigne [268, 270]. Un certain nombre d'entre eux sont d'ailleurs actifs *in vitro* et *in vivo*, mais les interactions entre ces remèdes et les traitements hospitaliers sont méconnues et mériteraient d’être explorées davantage [174].

### Gale

Romain Blaizot

La gale est présente de manière importante en Guyane. Des prévalences de 2 à 4% peuvent être retrouvées dans certains villages amérindiens du haut Oyapock ou parmi les populations migrantes vivant dans les habitats informels du littoral [215]. Le taux d’échec de traitement (persistance des lésions malgré une prescription de traitement approprié) y est d'environ un cas sur trois [Blaizot, données non publiées].

**Figure 38:**
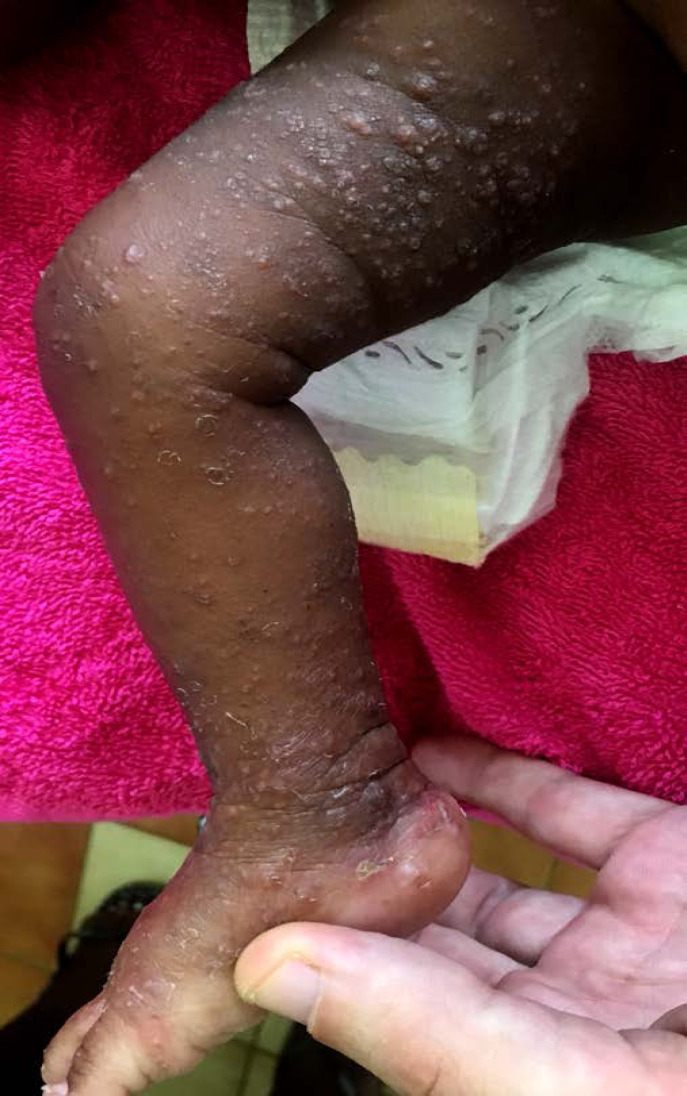
Lésions de gale (pustules, papules, nodules et sillons au niveau de la jambe) chez un enfant businenge (crédit photo: R. Blaizot) Scabies lesions (pustules, papules, nodules and grooves on the leg) in a Businenge child (photo credit: R.Blaizot)

Des conceptions spécifiques de la gale sont observées. La stigmatisation liée à la maladie est importante chez les Amérindiens, mais plus rare dans certaines communautés où la prévalence très élevée engendre une banalisation, comme à Javouhey (communauté ndjuka) (Fig. 38). Les Amérindiens ont souvent, du fait d'une acuité visuelle exceptionnelle, la capacité d'extraire à l'aiguille les parasites adultes, ce qui est également observé ailleurs en Amazonie [235]. En revanche, les capacités de décontamination (électroménager…) de l'environnement domestique sont souvent faibles. Les acaricides en spray peuvent être utilisés à condition d’être fournis gratuitement, du fait de leur prix élevé.

Les patients amérindiens et businenges présentent parfois une meilleure adhésion en cas de traitement topique (benzoate de benzyle en lotion, perméthrine crème), plus proche de leurs médecines traditionnelles et auxquelles une puissance plus importante est attribuée du fait de la sensation de brûlure occasionnée [Blaizot, données non publiées]. L'ivermectine *per os* peut être prescrite en association au topique ou en monothérapie pour les contacts asymptomatiques. Une discussion avec chaque patient est indispensable pour mesurer son adhésion au traitement et ses capacités de décontamination. L'utilisation de remèdes traditionnels est très fréquente et peut engendrer eczématisation ou surinfection. Hormis certains Amérindiens palikur, aucune communauté ne possède de remède traditionnel efficace contre la gale.

### Tungose ou « puce chique »

Justin Destoop, Pierre Couppié

La tungose ou puce chique est une ectoparasitose bénigne causée par la femelle fécondée de *Tunga penetrans*, de la famille des puces. L’être humain se contamine dans les zones tropicales par contact direct en marchant pieds nus sur les sols sableux chauds et humides, mais aussi forestiers. La puce mesure environ 1 mm lors de l'infestation, s'enkyste dans l’épiderme, ne laissant ouverts sur l'extérieur que les stigmates respiratoires et l'orifice de ponte.

**Figure 39:**
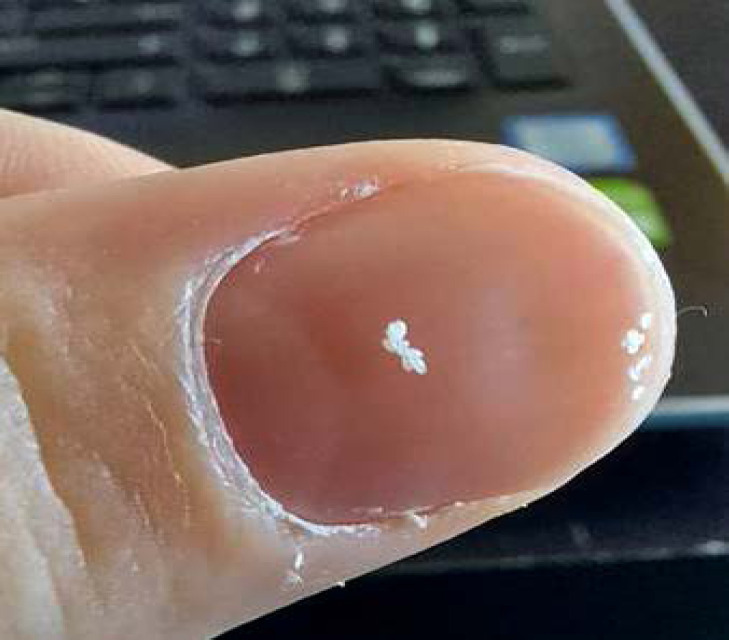
Œufs de puce chique (crédit photo: J. Destoop) Sand flea eggs (photo credit: J. Destoop)

Comme elle se nourrit du sang de son hôte, s'ensuit une phase de croissance où elle atteindra une taille de 5-7 mm, puis commencera sa phase de ponte au bout de 8-10 jours (200-250 œufs par puce) (Fig. 39). Cette dernière phase dure 3-4 semaines avant que la puce ne meure [168].

**Figure 40:**
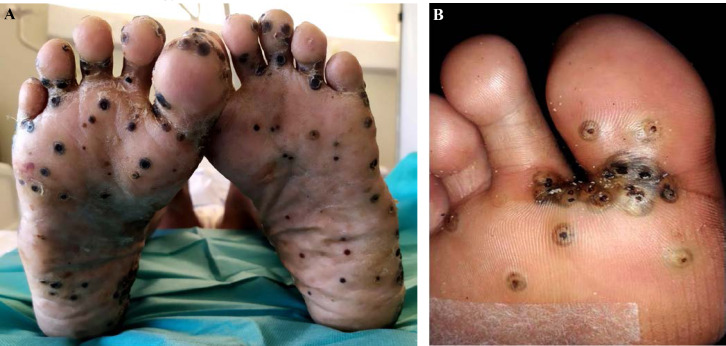
Tungose ou « puce chique » Légende: A. Patient de Cayenne (crédit photo: J. Destoop); B. Tungose profuse chez un patient alcoolique de Saint-Laurent-du-Maroni (crédit photo: P. Naudion) Tungiasis ou sand fleas infection A. Patient from Cayenne (photo credit: J. Destoop); B. Profuse tungiasis in an alcoholic patient from Saint-Laurent-du-Maroni (photo credit: P. Naudion)

Le diagnostic clinique est facile. La lésion est une papule arrondie, blanchâtre, centrée par un point noir, mesurant quelques millimètres. Elle se constitue en 5 à 7 jours et peut être symptomatique (prurit et douleur). Elle touche préférentiellement les pieds et notamment les zones péri-unguéales. Le nombre de puces peut varier d'une ou deux le plus souvent, jusqu’à des formes profuses retrouvées dans des contextes pathologiques avec hypo-esthésie: éthylisme chronique, lèpre et troubles psychiatriques (Fig. 40). Les complications possibles sont les infections bactériennes des lésions (streptocoque, staphylocoque), le tétanos et les réactions inflammatoires locales [364]. Le traitement consiste en une extraction mécanique après désinfection. Dans les formes profuses, une occlusion pendant 12-24 h avec de la vaseline salicylée à 20% a montré de bons résultats [63]. La vérification du statut tétanique et l’éventuelle séro-vaccination restent indispensables.

### Larva migrans cutanée ankylostomienne

Morgane Bourne-Watrin, Justin Destoop, Loïc Epelboin

**Figure 41:**
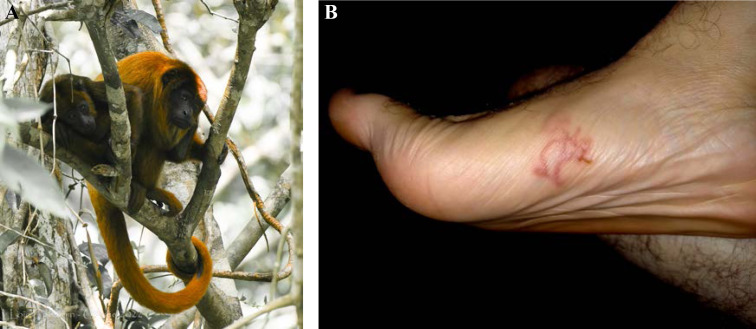
A. Singe hurleur roux (*Alouatta macconnelli*), femelle et petit, centre de sauvegarde ONCA, Montsinéry (crédit photo: L. Epelboin) B. Lésions de larva migrans cutanée ankylostomienne suite à des missions dans la Réserve naturelle nationale des Nouragues (crédit photo: J. Tribot) *A. Red howler monkey* (Alouatta macconnelli), *female and cub, ONCA rescue centre, Montsinéry (photo credit: L. Epelboin) B. Hookworm larva migrans lesions following missions in the Nouragues National Nature Reserve (photo credit: J. Tribot)*

Aussi appelée dermatite rampante ou « vers chien » par les créoles, il s'agit d'une dermatite urticarienne liée à une impasse parasitaire d'une larve de nématode dont les plus fréquents sont *Ancylostoma caninum* et *A. braziliense* [1]. Les hôtes habituels sont le chien et le chat, mais les primates semblent être porteurs et pouvoir transmettre la maladie comme en atteste la présence sérologique chez les primates de zoos américains et un cas de contamination après manipulation de selles de singe hurleur roux (*Alouatta macconnelli*) (Fig. 41A) [4]. Le mode de contamination le plus connu est sur la plage après s’être allongé près d'un sable contaminé par des selles de chiens, mais aussi chez les enfants en milieu rural pauvre jouant pieds nus sur du sol sablonneux en saison des pluies comme dans la région de Manaus au Brésil [3]. Plus atypiques, des cas ont été décrits après séjour en forêt amazonienne possiblement en lien avec un cycle chez les singes hurleurs [1]. L'incubation est classiquement de 10 à 21 jours, le prurit est quasi constant et il existe un trajet serpigineux avec migration de plusieurs mm par jour (Fig. 41B). Les cas autochtones sont classiquement multilésionnels et des cas atypiques comme la folliculite ankylostomienne sont possibles [2]. Cette forme correspondant à des papulopustules et non une dermatite rampante dans les zones ayant été en contact prolongé (dos et fesses) avec le sol (plomberie, mécanique…) chez des personnes possédant des chiens ou chats [3]. L'hémogramme peut retrouver une hyperéosinophilie. Les mesures de prévention sont le port de chaussure fermée à la plage ou en zone péridomicilaire, l'utilisation d'un matelas en plastique plutôt qu'une serviette à la plage ou s'allonger sur du sable mouillé, ramasser les déjections canines sur la plage. Le traitement repose sur l'ivermectine 200 µg/kg pouvant être renouvelé une fois puis albendazole 400 mg pendant 3 à 7 jours si échec [1].

### Myiases

Morgane Bourne-Watrin, Pierre Couppié

Les myiases humaines sont des infections parasitaires négligées et sous-étudiées pour lesquelles il est important d'améliorer les connaissances des patients et des professionnels sur leur traitement et leur prévention. Les plaies cutanées, la consommation de toxiques et les maladies mentales sont les principaux facteurs de risque [47].

#### Myiase furonculoïde sud-américaine ou « ver macaque »

Cette infection est liée aux larves du diptère *Dermatobia hominis* qui infecte l’être humain par un phénomène de phorésie: la mouche femelle va capturer un moustique et coller ses œufs sous l'abdomen de celui-ci. Lorsque le moustique pique l’être humain, il dépose sur la peau les larves qui la pénètrent suite au grattage [305]. La maturation des larves de *D. hominis* prend ensuite entre 3 semaines et 3 mois. Les régions touchées sont les zones découvertes car, contrairement à son cousin africain le ver de Cayor (*Cordylobia anthropophaga*), les larves du ver macaque ne se retrouvent pas sur le linge mis à sécher. La contamination est diurne et en milieu rural. Il faut donc faire attention aux patients qui présentent une plaie suite à une piqûre en zone urbaine moins d'une semaine auparavant et qui la manipulent en pensant être infectés par un ver macaque: si le cycle ne correspond pas, il faudra plutôt penser à un furoncle ou un abcès en voie de formation et d'aggravation suite aux manipulations !

**Figure 42:**
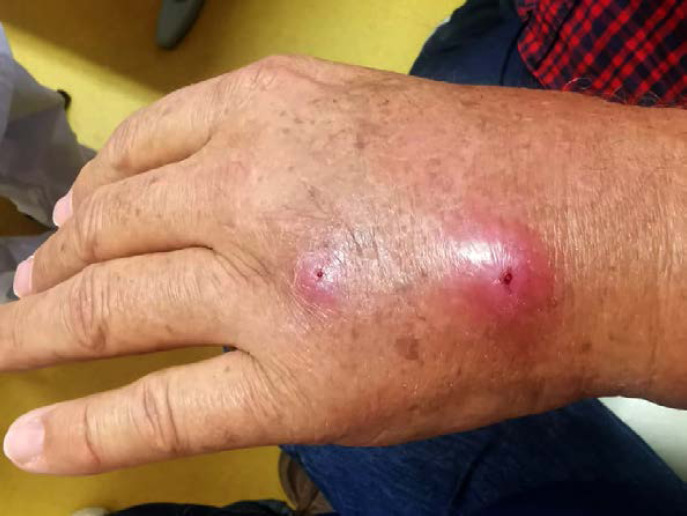
Lésion de ver macaque sur le dos de la main (crédit photo: J. Destoop) Human botfly (Dermatobia hominis) lesion on the back of the hand (photo credit: J. Destoop)

La lésion est un papulo-nodule qui ressemble effectivement à un furoncle (Fig. 42), d'intensité douloureuse inhabituelle, centré par un orifice d'où on peut voir le mouvement ou la respiration de la larve. Pour mieux l'apercevoir, on peut recouvrir la lésion avec de l'eau afin de la forcer à « sortir » pour respirer, et tenter de la voir avec une loupe. La douleur sera intermittente et il y aura une sensation de « mouvement à l'intérieur de la plaie ». Les atteintes multiples sont possibles, mais il n'y aura qu'une seule larve par lésion. La manipulation intempestive de la lésion est fréquente, le patient essayant en vain de réaliser une extraction manuelle de la larve avec risque de surinfection bactérienne. Si un bilan biologique est réalisé, il sera normal sauf en cas d'infestation récurrente ou de lésions multiples où un syndrome inflammatoire ou une hyperéosinophilie sont possibles.

**Figure 43:**
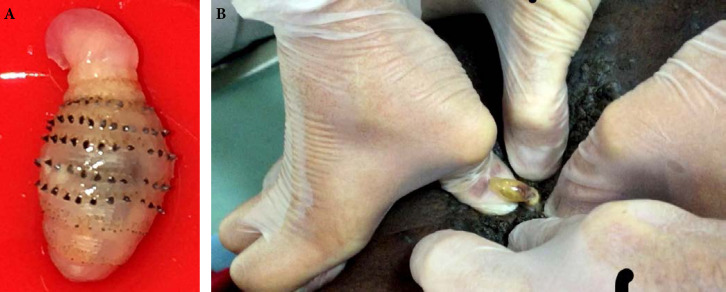
Myiase furonculoïde Légende: A. Larve de Dermatobia hominis (crédit photo: J. Destoop); B. Extraction à 4 mains d'un ver macaque de la fesse, Maripasoula (crédit photo: L. Epelboin) Furuncular myiasis A. Dermatobia hominis larva (photo credit: J. Destoop); B. Four-handed extraction of a botfly from the patient's buttock, Maripasoula (photo credit: L. Epelboin)

Le traitement consiste en l'extraction des larves, mais celles-ci ayant une forme de bouteille de Perrier^©^ (Fig. 43A) avec la partie la plus large à l'intérieur de la plaie, il n'est pas possible de la retirer seul. Il faut dans un premier temps essayer de la faire migrer à la surface en la recouvrant de vaseline pour l’étouffer. On peut aussi appliquer de l'ivermectine topique ou pilée dans la vaseline pour adjoindre un effet anti-parasitaire. L'ivermectine *per os* n'est pas nécessaire, de même que la prescription systématique d'antibiotiques s'il n'y a pas de signe de surinfection. L'extraction se fera après au moins quatre heures d'application de ce traitement topique, à quatre mains, en poussant sur les bords de la lésion et en profondeur pour faire « sauter » la larve (Fig. 43B). Attention à bien porter un masque pour ne pas avaler par mégarde celle-ci lorsqu'elle sera extraite !

#### Myiases des plaies et cavitaires

**Figure 44:**
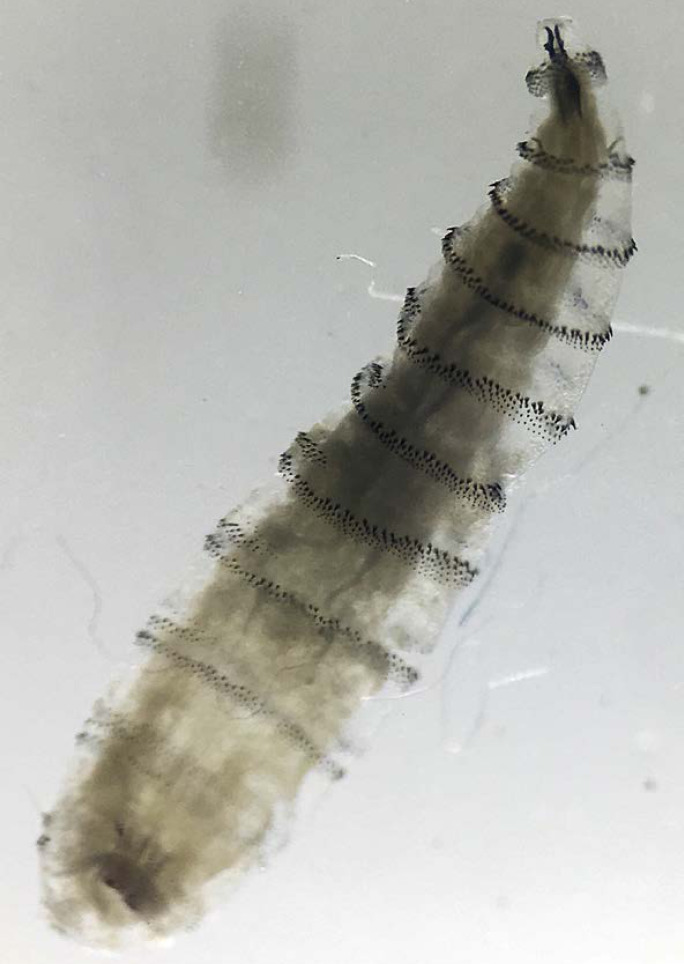
Larve de *Cochliomyia hominivorax* colonisant une cavité post-chirurgicale de cholestéatome (crédit photo: A. Mathieu) Cochliomyia hominivorax *larva colonizing a post-surgical cholesteatoma cavity (photo credit: A. Mathieu)*

**Figure 45:**
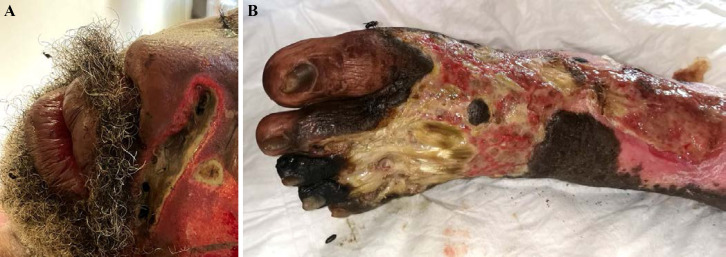
Myiases des plaies Légende: A. Myiases des plaies au niveau de la narine chez un patient épileptique hémiparétique (crédit photo: E. Borg); B. Mouche posée sur une plaie nécrotique lors de la réfection du pansement (crédit photo: M. Bourne Watrin) Wound myiases A. Nostril wound myiases in a hemiparetic epileptic patient (photo credit: E. Borg); B. Fly settled on a necrotic wound during the re-dressing process (photo credit: M. Bourne Watrin)

*Cochliomyia hominivorax* est le principal agent responsable des myiases des plaies et des cavités en Amérique du Sud [250] (Fig. 44). Ces infections sont plus fréquentes sous les tropiques en raison du climat chaud et humide et des mauvaises conditions socio-économiques et donc d'hygiène de certaines populations [47]. Elles peuvent également être acquises de manière nosocomiale si les fenêtres et portes des centres de soins sont ouvertes. Les larves vont contaminer toutes les plaies: ulcères, escarres, lésions cancéreuses avec plusieurs larves par lésion, parfois plus de cent. Il faudra penser à vérifier les cavités des patients dans le coma ou présentant des atteintes cognitives (Fig. 45A) et à recouvrir toute plaie, éviter de laisser les pansements trop longtemps ouverts lors de leur réfection (les refermer avec une alèse si on doit s'arrêter au milieu de la tâche) car les mouches veillent au grain (Fig. 45B) !

Les larves seront extraites à la pince après une application locale de comprimés d'ivermectine (4-5 comprimés) pilés avec de la vaseline dans un pansement occlusif pendant 24 heures dans la plaie ou dans la cavité à traiter [73]. Ce traitement local peut être plus ou moins complété par une prise d'ivermectine *per os* si le site à traiter est difficile d'accès, mais il n'y a pas de traitement consensuel. Il est urgent de faire le diagnostic car les larves de *C. hominivorax*, comme leur nom l'indique, vont « manger l’être humain », à savoir les tissus sains, y compris les cartilages et l'os, laissant des destructions irréversibles.

### Pou d'agouti

Morgane Bourne-Watrin, Pierre Couppié

**Figure 46:**
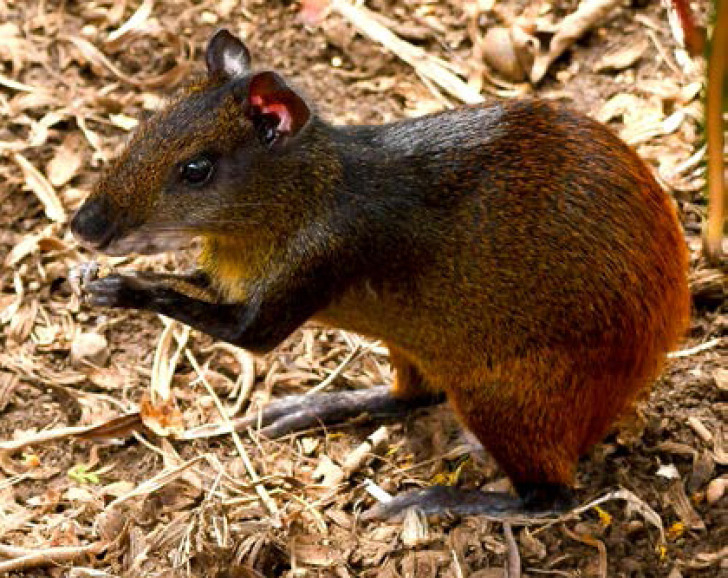
Agouti à cul roux *(Dasyprocta leporina)* aux Iles du Salut (crédit photo: L Epelboin) *Red-rumped agouti* (Dasyprocta leporina) *in Iles du Salut (photo credit: L. Epelboin)*

Cet aoûtat de la famille des Trombiculidae porte mal son nom car il n'est pas transmis par l'agouti (*Dasyprocta leporina*), un rongeur amazonien couramment observé dans tous les milieux (Fig. 46). Il tire simplement son nom de la couleur rose-cuivrée des lésions qu'il entraîne et qui rappellent le pelage du rongeur. Les larves sont présentes dans l'herbe, s'accrochant aux animaux et aux êtres humains à leur passage [68]. En impasse parasitaire, l'acarien va piquer sur son passage principalement aux zones de striction où il se sentira acculé (élastiques des sous-vêtements et chaussettes, grands plis).

**Figure 47:**
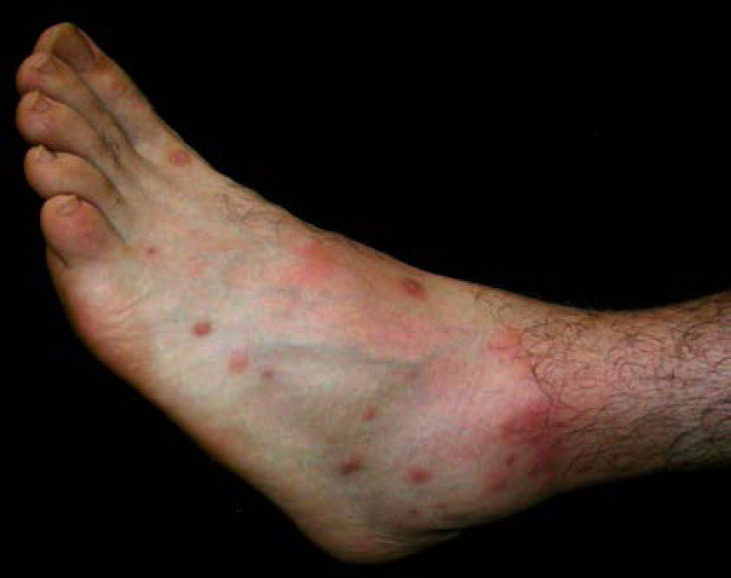
Poux d'agouti du pied gauche (crédit photo: P. Couppié) Agouti lice on left foot (photo credit: P. Couppié)

Les lésions secondaires à la piqûre sont des papules très prurigineuses (Fig. 47), pouvant aller jusqu'au prurigo ou aux bulles selon l'intensité du prurit et de la réaction inflammatoire locale. Ces acariens ne transmettent pas de maladie mais induisent un prurit féroce, insomniant et sont à risque de surinfection cutanée secondaire. Il existe une désensibilisation progressive aux piqûres chez des personnes vivant de façon prolongée en zone d'endémie.

Les mesures préventives sont le port de vêtements couvrants ou l'application de répulsifs notamment naturels comme l'huile de carapa (une plante de la famille des Méliacées, facilement trouvable en Guyane) qui aurait également des propriétés anti-infectieuses.

Le traitement est principalement symptomatique avec antihistaminiques, dermocorticoïdes dans les cas extrêmes ou l'huile de carapa de nouveau à visée anti-infectieuse, inflammatoire et apaisante [18]. Sans traitement, les lésions disparaissent spontanément en moins d'une semaine. L'Ascabiol^©^ est utilisé par certains, mais il a peu d'effet car le parasite ne reste pas sur le corps et ce traitement, plutôt irritant, ne soulage pas le prurit.

### Papillonite ou lépidoptérisme

Morgane Bourne-Watrin, Pierre Couppié

**Figure 48:**
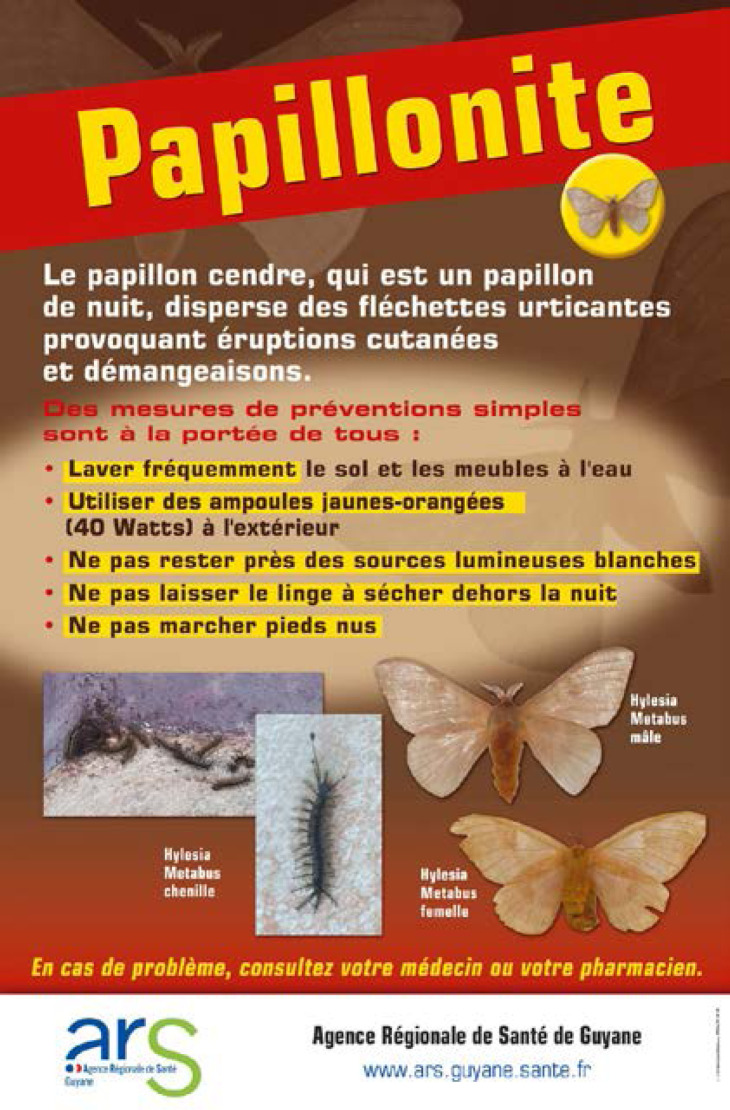
Affiche de prévention contre la papillonite *(Hylesia metabus)* (source: ARS Guyane) Lepidopterism prevention poster (Hylesia metabus) (source: ARS Guyane)

Il s'agit d'une dermatite liée à l'envenimation par les fléchettes microscopiques présentes sous l'abdomen des femelles du papillon cendre, *Hylesia metabus* (ex-H. *urticans)* [187] (Fig. 48). Le cycle larvaire de ce papillon dure trois mois avec des éclosions synchrones en janvier, avril, juillet et octobre, donnant lieu à des phénomènes épidémiques plus ou moins importants selon les années. Ces papillons sont présents sur le littoral au niveau des mangroves, là où se trouvent les palétuviers et ils ont une activité crépusculaire. Ils sont attirés par les lumières artificielles blanches. Les communes les plus touchées récemment étaient Sinnamary et Iracoubo. Les épidémies de papillonite étaient fréquentes sur l’Île de Cayenne il y a encore 10 ans, mais la destruction de la mangrove a fait disparaître l'habitat du papillon. Le retour cyclique de la mangrove peut entraîner le retour des papillons cendre et de la papillonite, comme cela s'est produit à Kourou durant l’été 2022 et à Cayenne en octobre de la même année.

Il faut rechercher à l'interrogatoire un contact avec ou l'observation de papillons en grand nombre, mais celui-ci est parfois absent. La réaction cutanée peut avoir lieu suite à un contact direct, mais aussi indirect suite à la chute de fléchettes sur des vêtements pendant la nuit par exemple. Il est possible d'en voir en dehors des pics épidémiques si le patient a mobilisé un cadavre de femelle du papillon. Il arrive que des cas surviennent de façon groupée chez des personnes ayant fréquenté les mêmes lieux.

**Figure 49:**
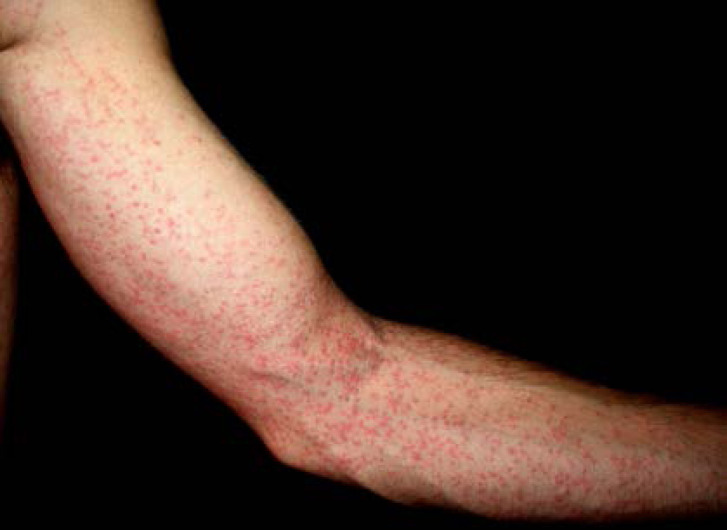
Lésions de papillonite du bras gauche (crédit photo: P. Couppié) Lepidopterism lesions on left arm (photo credit: P. Couppié)

L'examen clinique retrouve une dermatite urticarienne très prurigineuse avec de multiples points rouges correspondant aux points d'impact des fléchettes (Fig. 49). Elle peut évoluer vers un prurigo vésiculeux. L’éruption sera le plus souvent en zone découverte, sauf si le patient s'est envenimé par contact indirect en mettant ses vêtements qui avaient été laissés la veille à sécher sur une corde à linge: l’éruption sera alors en zone couverte. Les lésions apparaissent dans les 15 minutes après le contact et persistent entre 2 et 7 jours selon que la personne a déjà été exposée ou non. De rares atteintes oculaires ou respiratoires sont possibles si ces zones ont été exposées. Le prurit peut persister un an après l'atteinte et comme pour toute dermatose prurigineuse, il existe un risque de surinfection.

À titre de comparaison, cette atteinte s'apparente à celle secondaire au contact avec les chenilles processionnaires de l'Hexagone. Il s'agit d'une réaction histaminique et non d'une infection.

Le traitement symptomatique à base d'antihistaminiques et de dermocorticoïdes n'est pas toujours efficace. Les bains d'eau très chaude peuvent soulager au début par action sur le venin thermolabile. L'extraction des fléchettes peut aussi être tentée à la pince à épiler ou au ruban adhésif. Dans les formes très profuses avec atteinte muqueuse, des antalgiques de palier 2, anesthésiques locaux et corticoïdes systémiques peuvent être utilisés [279].

Les mesures sont surtout préventives: campagnes d'information, éviter les éclairages extérieurs de couleur blanche remplacés par du jaune-orangé, couvrir les plats et les verres pour éviter que les fléchettes ne tombent dedans, laver les sols, meubles, hamacs, ne pas laisser les vêtements sécher à l'extérieur. Il faut éviter les bombes insecticides car les insectes attaqués relarguent les fléchettes.

### Yens-yens

Loïc Epelboin, Morgane Bourne-Watrin

**Figure 50:**
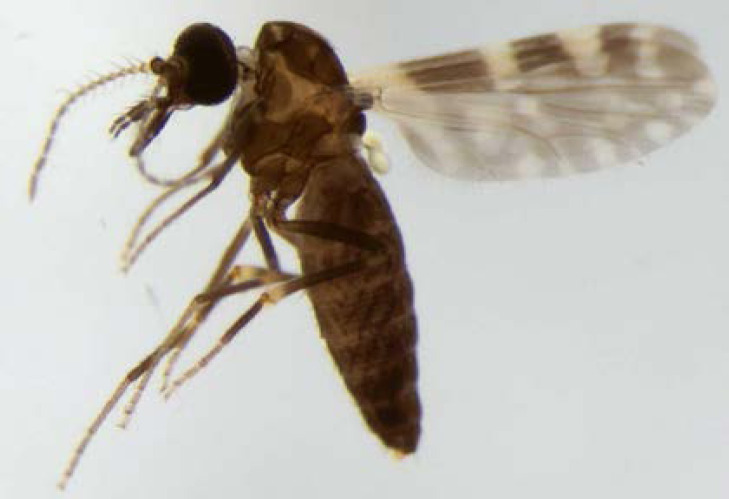
Femelle *Culicoides hylas*, collectée à Antecume Pata par Talaga, Lacour & Issaly, Mars 2018 (photo et identification: J.-B. Duchemin, IPG) *Female* Culicoides hylas, *collected at Antecume Pata by Talaga, Lacour & Issaly, March 2018 (photo and identification J.-B. Duchemin, IPG)*

Les culicoïdes dont le nom vernaculaire créole est yens-yens, sont des petits moucherons dont les larves se développent généralement dans le sable humide, la vase ou la boue^1^1Agence Régionale de Santé (ARS) Guadeloupe. Guide pour l’élaboration des plans communaux de lutte contre les moustiques et de prévention des maladies vectorielles. 2009. 2^e^ mise-à-jour Janvier 2018: 1-62. https://www.guadeloupe.ars.sante.fr/index.php/media/27091/download?inline. (Fig. 50). La morsure de la femelle est ressentie comme une piqûre parfois assez vive, souvent suivie d'une réaction érythémateuse et d'une démangeaison, qui disparaissent en quelques heures à quelques jours. Il existe de nombreuses espèces que l'on trouve sous pratiquement toutes les latitudes. L'aspect de la piqûre de yens-yens n'a rien de particulier ni dans sa topographie ni dans sa présentation clinique et ressemble à une piqûre de moustique. Ils sont fréquemment actifs sur les plages de l’île de Cayenne en fin de journée. Leur nuisance est surtout liée au prurit provoqué par les lésions qu'ils provoquent, mais ils ne sont pas connus pour transmettre des maladies en Guyane. La prévention repose essentiellement sur la protection vectorielle (vêtements longs++) et le traitement curatif, exceptionnellement sur des dermocorticoides en cas de prurit mal toléré.

## Pathologies Tropicales Non Infectieuses

### Intoxications

#### Intoxication par les plantes

Théo Blaise, Guillaume Odonne

La diversité du territoire guyanais revêt plusieurs aspects et notamment celle des écosystèmes et des pharmacopées traditionnelles. La population guyanaise utilise bon nombre de remèdes traditionnels pour se soigner, dont la plupart sont issus du monde végétal.

Les intoxications – accidentelles ou volontaires – impliquant des plantes ne sont pas rares; elles concernent toutes les tranches d’âge de la population et peuvent représenter un risque sanitaire important. La diversité botanique peut être source de confusion dans la préparation de remèdes traditionnels, tout comme la méconnaissance du danger de certaines plantes toxiques. Fort heureusement, dans la majorité des cas, ces intoxications sont bénignes et résolutives après quelques heures. Toutefois, certaines peuvent se révéler graves, voire mortelles.

À partir d'une base de données de toxicovigilance constituée au cours des 20 dernières années, environ 200 cas d'intoxication impliquant des plantes ont été identifiés sur le territoire [26]. Chez l'adulte, les intoxications accidentelles impliquaient le plus souvent le manioc (*Manihot esculenta*), qui provoque une intoxication cyanogénique se manifestant par des troubles neurologiques importants lorsqu'il est mal préparé. En effet, plusieurs variétés de manioc nécessitent une préparation particulière, souvent assez longue, qui lui permet de perdre sa toxicité. L'utilisation d'huile de ricin *(Ricinus communis)* mal préparée à visée purgative peut entraîner une défaillance multi-viscérale mortelle. On peut citer également une particularité du territoire, l'intoxication volontaire avec de la nivrée *(Lonchocarpus* spp.): des lianes ichtyotoxiques (toxiques pour les poissons), riches en roténone, qui sont utilisées sur les fleuves Maroni et Oyapock au cours des pêches traditionnelles.

**Figure 51:**
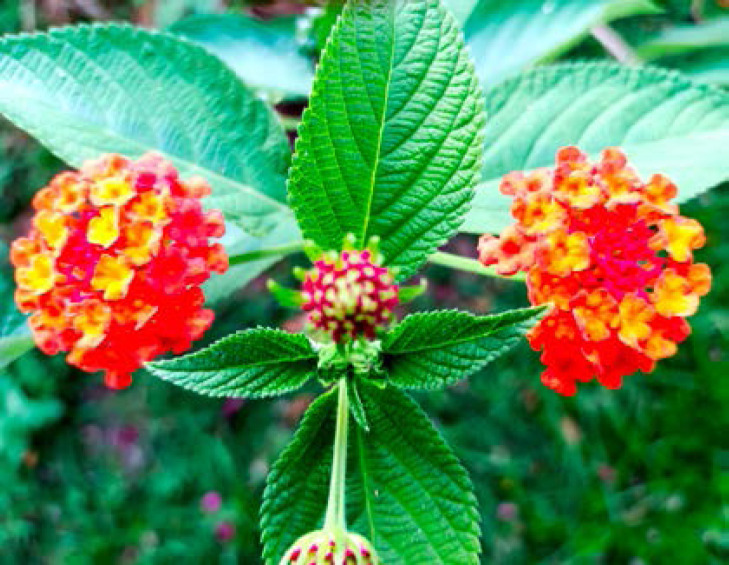
Lontana ou marie-crab *(Lontana camara)* dans un jardin de Cayenne (crédit photo: T. Blaise) Lantana camara *in a Cayenne garden (photo credit: T. Blaise)*

**Figure 52:**
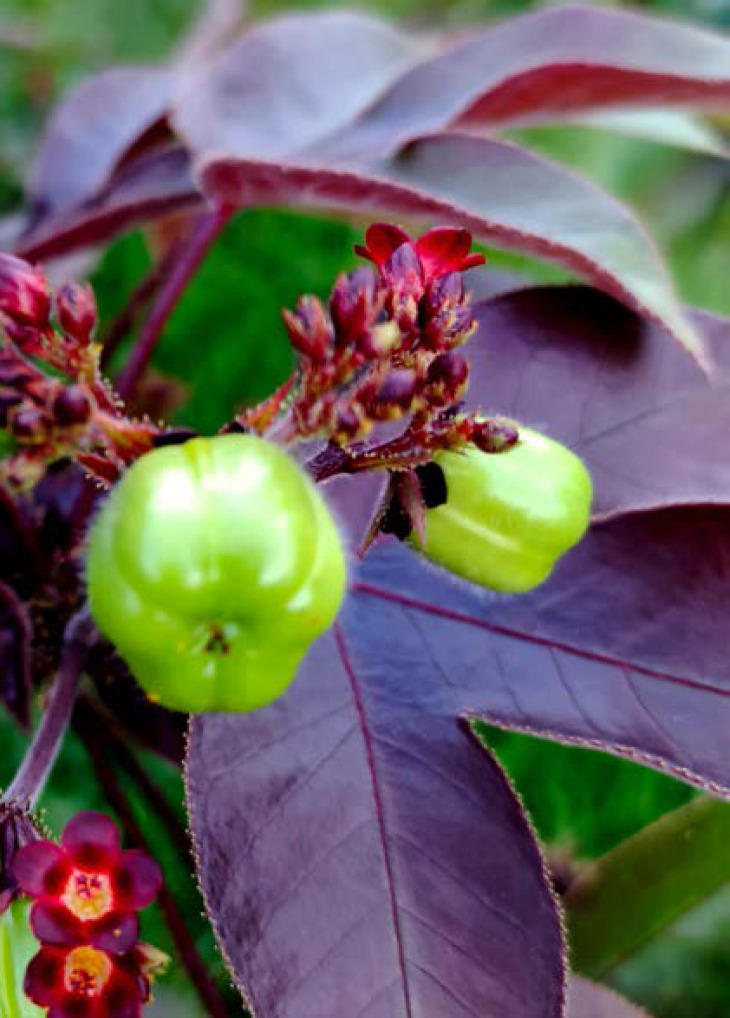
Médicinier Rouge *(Jatropha Gossypiifolia)* dans un jardin de Cayenne (crédit photo: T. Blaise) *Bellyache bush* (Jatropha Gossypiifolia) *in a Cayenne garden (photo credit: T. Blaise)*

Chez les enfants, c'est souvent l'ingestion de baies présentes dans les jardins qui est responsable des intoxications, bénignes dans la plupart des cas – avec le lantana ou marie-crab *(Lantana camara)* (Fig. 51) ou bien avec la famille du jatropha *(Jatropha curcas* et *Jatropha gossypiifolia)* de la famille des Euphorbiacées (Fig. 52). Une autre spécificité locale concerne les macérats de tabac *(Nicotiana tabacum)* destinés à être inhalés. Ces préparations sont largement répandues dans la culture businenge. Le liquide, souvent contenu dans des bouteilles de plastique, est ingéré par de jeunes enfants et peut provoquer des troubles cardiaques importants voir le décès.

Afin d'améliorer la prise en charge de ces intoxications dans un contexte d'urgence, un travail est en cours pour mettre au point des fiches pratiques qui seront associés à un réseau de botanistes permettant, à terme, d'accélérer l'identification des espèces incriminées et de proposer une prise en charge adaptée pour les soignants.

#### Intoxication à la bita

Frédégonde About, Guillaume Odonne

**Figure 53:**
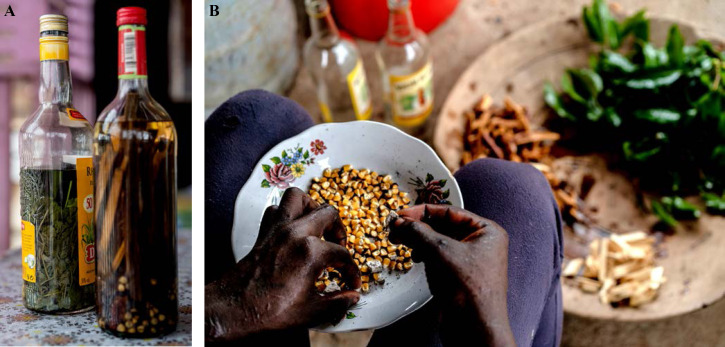
A. Bouteille de bita; B. Préparation d'une bouteille de bita par un tradipraticien ndjuka dans la commune d'Apatou (crédit photos: K. Joseph) A. Bottle of bita; B. Ndyuka tradipratician preparing a bottle of bita in Apatou commune (photo credits: K. Joseph)

La bita, du sranan tongo, la langue du Suriname, terme dérivé de l'anglais *bitter*, amer, est une boisson amère issue de la macération de plantes dans de l'eau, du vermouth ou/et du rhum, contenue dans un récipient en verre ou en plastique. Elle est consommée pour ses vertus médicinales fortifiantes, dépuratives, antalgiques (contre les douleurs abdominales) mais aussi aphrodisiaques (Fig. 53). La bita se boit sous forme de « shot » équivalent à un bouchon de bouteille. On peut également la consommer dans un récipient creusé dans le bois du *Quassia amara* (le couachi, une plante très utilisée dans les pharmacopées locales) que l'on remplit d'eau ou de rhum, qui prendra un goût amer en 20-30 minutes [269]. Ce récipient est appelé la « bita cup ».

Par tradition, la connaissance du monde végétal et son utilisation sont transmises de génération en génération au sein de la communauté businenge. Certains savoirs plus spécialisés et plus secrets sont réservés aux tradipraticiens. Cependant, la communauté businenge subirait une acculturation qui pourrait, selon certains, être génératrice d'erreurs de reconnaissance botanique et par conséquent responsable d'intoxications. En effet, des séries de cas sévères, parfois mortels, ont été observées en 2009 et 2010 suite à la consommation de bita. Une investigation menée par l'ARS a permis de la mettre en cause de manière certaine [209]. Les symptômes de ces intoxications à la bita étaient très variés: digestifs (mucite, vomissements, constipation), cutanés (épidermite périscrotale, alopécie, desquamation palmoplantaire, hyperpigmentation) et neurologiques (syndrome méningé, paresthésies, hypoesthésie, aréflexie, parésie sévère). La biologie pouvait retrouver des cytopénies (leucopénie, thrombopénie, anémie), une élévation des LDH, une cytolyse hépatique, une élévation de la CRP.

Plusieurs hypothèses peuvent être formulées quant à la raison de ces intoxications à la bita encore observées aujourd'hui: un mésusage de la bita (consommation trop importante), une méconnaissance actuelle de sa préparation (erreur d'identification botanique), ouune adultération intentionnelle avec d'autres substances chimiques tirées de spécialités pharmaceutiques, ce phénomène d'hybridation entre médecines étant régulièrement observé par ailleurs.

Depuis l'investigation par l'ARS en 2010, des projets de protocoles de surveillance et d'investigation ont été évoqués. Il convient de rechercher et déclarer tout cas de suspicion d'intoxication à la bita à l'ARS Guyane.

#### Le pemba

**Figure 54:**
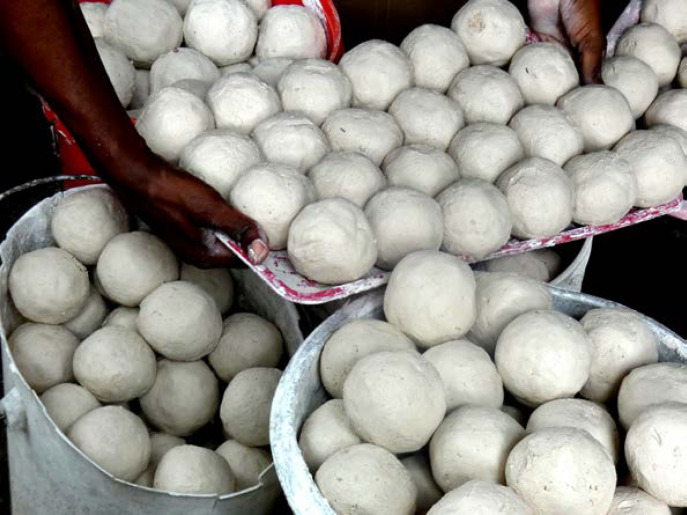
Boules de pemba en préparation dans un village côtier du Suriname (crédit photo: M.-A. Tareau) Preparation of pemba balls in a coastal village of Suriname (photo credit: M.-A. Tareau)

Le pemba est un kaolin, une argile fine et blanche préparée artisanalement sous forme de boules de différentes tailles [343] (Fig. 54). Cette pâte d'argile contient un fort taux d'aluminium. Une fois récoltée et séchée au soleil pendant un ou deux jours, la pâte devient une boule dure. Friable, elle redevient poussière. Mélangée à la salive, elle retrouve sa forme pâteuse. Sa consommation est fréquente chez les femmes enceintes de l'ouest guyanais ou parmi les migrantes à Cayenne et pourrait provoquer une anémie ou une intoxication, au plomb ou par d'autres métaux lourds, du fœtus [211, 313].

Ainsi, les intoxications par des produits traditionnels tels que la bita et le pemba sur l'ouest guyanais font l'objet d'une veille sanitaire sur le territoire. Ces signalements peuvent aboutir à des alertes sanitaires mais aussi à de nouvelles investigations qui permettraient de mieux identifier les causes des intoxications par produits traditionnels afin de pouvoir les prévenir au mieux.

#### Intoxication au paraquat

Lindsay Osei, Hatem Kallel, Loïc Epelboin

Le paraquat est un herbicide de contact non sélectif qui détenait la plus grande part du marché mondial des herbicides jusqu’à récemment, lorsqu'il a été dépassé par le glyphosate.

En raison de sa toxicité élevée, l'Union européenne l'a retiré de son marché en 2007. Alors que le paraquat a presque disparu de la France hexagonale, la Guyane se trouve dans une situation différente en raison des pays voisins qui autorisent encore son utilisation, notamment le Suriname. Son ingestion (souvent à des fins de suicide) peut provoquer une défaillance multiviscérale. En pratique clinique, aux urgences et en réanimation, la situation est considérée comme grave en cas d'ingestion d'un volume à partir d'un demi-verre, ou une gorgée de solution à 200 g/l soit 35 mg/kg chez l'adulte.

Après l'ingestion, le pic plasmatique est atteint en 2 heures, suivi d'une redistribution tissulaire vers les reins, le foie et les poumons avec une demi-vie de 12 heures. Les caractéristiques cliniques sont les suivantes: après l'ingestion de 20-40 mg/kg, les patients présentent rapidement des vomissements et des douleurs ulcéreuses pharyngées suivies d'une insuffisance rénale et d'une cytolyse hépatique. Ensuite, une altération progressive de la fonction respiratoire apparaît, due d'abord à une alvéolite inflammatoire avec destruction des pneumocytes, puis à une fibrose pulmonaire étendue, conduisant à une hypoxémie réfractaire et au décès en 15-28 jours. À l'inverse, lorsque la dose ingérée est supérieure à 40 mg/kg, la mort est rapide, en 24-72 h, due à une insuffisance circulatoire aiguë [132]. La détermination, le plus tôt possible, de la concentration plasmatique en paraquat a une excellente valeur pronostique, avec un taux de mortalité proche de 100% pour un taux plasmatique > 2 mg/l quatre heures après l'ingestion. Cependant, ce test n'est pas disponible partout et en son absence, la paraquaturie, appréciée par un test semi-quantitatif sur bandelettes colorimétriques au dithionite, permet une évaluation rapide et fiable de la sévérité de l'intoxication. De 2008 à 2015, 62 patients ont été hospitalisés en Guyane pour intoxication au paraquat, parmi lesquels 44 adultes et 18 enfants de moins de 16 ans, avec un sex-ratio de 1. Les âges médians étaient respectivement de 31 ans chez les adultes et de 13 ans chez les enfants. Sur la totalité, 67% provenaient du Maroni (Saint-Laurent, Grand Santi, Papaïchton et Maripasoula) et aucun cas n'a été enregistré dans l'est du pays. La durée médiane de l'hospitalisation était de 15,5 jours chez les enfants et de 2 jours chez les adultes. La majorité des cas étaient dus à un auto-empoisonnement (84%). La mortalité était élevée: 65% de décès chez les adultes contre 22% chez les enfants. Le principal facteur affectant le pronostic des patients était la quantité de paraquat ingérée. L'administration de charbon actif par voie orale ou de pemba, dans la première heure après l'ingestion de paraquat, est indispensable [113]. Une autre étude a été menée chez 26 patients intoxiqués au paraquat admis aux urgences du CHOG entre janvier 2008 et août 2014 [132]. Six patients ont survécu et 20 sont décédés dans un délai médian de 36 heures après l'admission. Le décès était associé à une défaillance hémodynamique (65%) et respiratoire (35%). Les facteurs prédictifs de décès comprenaient un âge avancé, une dose de paraquat ingérée plus élevée, une altération de la fonction rénale, une hypokaliémie, une acidose métabolique et un test au dithionite bleu foncé, évalué à l'admission à l'hôpital.

#### Intoxication au mercure

Maylis Douine

**Figure 55:**
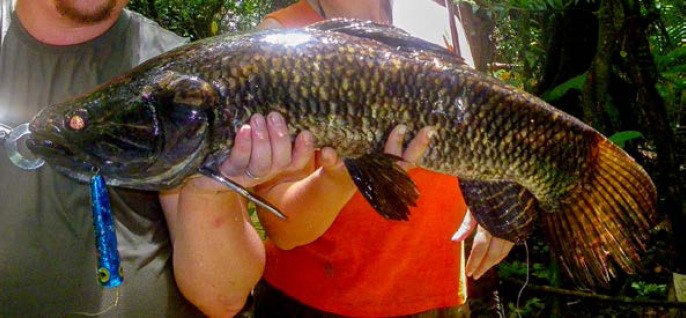
Pêche à l'aïmara au leurre (*Hoplias aimara*) (crédit photo: L. Epelboin) *Haimara* (Hoplias aimara) *lure fishing (photo credit: L. Epelboin)*

Les intoxications aux métaux lourds, notamment le mercure et le plomb, font partie des grandes priorités de santé publique en Guyane [161]. Le sol est naturellement riche en mercure, et l'utilisation pendant des décennies de ce métal pour amalgamer les particules d'or (interdit depuis 2006 par arrêté préfectoral n° 1232/SG du 8 juin 2004, mais encore utilisé par les orpailleurs illégaux) entraîne un relargage dans l'environnement de sa forme inorganique. Transformé en méthylmercure dans les écosystèmes aquatiques, il s'accumule par bioamplification dans la chaîne alimentaire. Les poissons carnivores peuvent ainsi contenir des concentrations très élevées [197]. La contamination humaine peut se faire par inhalation de vapeurs de mercure métallique et peut entraîner des tableaux respiratoires à type de bronchite ou pneumopathie interstitielle, comme ce cas de pneumopathie hypersensible décrit au CHC en 2021 chez un orpailleur [13]. Mais la contamination reste majoritairement digestive, par consommation de poissons notamment piscivores comme l'aïmara (*Hoplias aimara*) (Fig. 55) avec une neurotoxicité qui concerne particulièrement le fœtus et l'enfant en bas âge [136]. Depuis 1994, plusieurs études menées en population générale, chez des enfants et des femmes enceintes, retrouvent des concentrations en mercure plus élevées parmi les populations amérindiennes, en particulier vivant en dehors des bourgs, probablement du fait d'un mode de vie plus traditionnel avec une consommation importante de poissons [49, 293]. Une étude publiée en 1997 montrait une corrélation entre une augmentation des réflexes tendineux (non reproductible), une moindre coordination des jambes et une diminution du score « Copying test » (test non spécifique évaluant l'organisation visuo-spatiale) et la concentration capillaire de mercure de la mère notamment sur le haut Maroni. Un dépistage des femmes en âge de procréer ou enceintes et des enfants de moins de 7 ans résidant dans les bourgs isolés de Guyane ou consommant plus de 2 portions de poisson par semaine est recommandé (dosage capillaire = sur mèche de cheveux proche du crâne de 0,5 cm de diamètre, valeur seuil = 11 µg/g) [366]. La prise en charge repose essentiellement sur l’éviction des poissons piscivores, les traitements chélateurs n'ayant pas prouvé leur efficacité pour l'intoxication au mercure organique [366].

#### Intoxication au plomb

Maylis Douine

**Figure 56:**
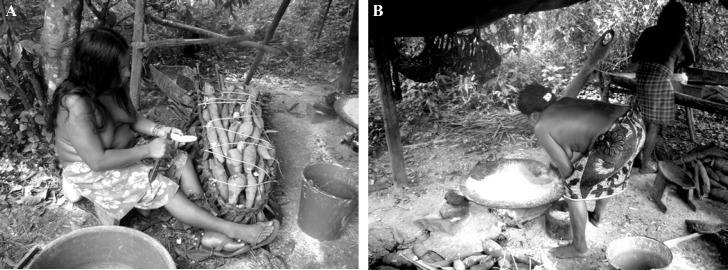
Préparation de la farine de manioc par des femmes Wayãpi à Trois-Sauts. A. Épluchage des tubercules de manioc; B. Cuisson du manioc râpé sur de grandes plaques de métal (crédit photos: L. Epelboin) Wayãpi women preparing manioc flour in Trois-Sauts. A. Peeling cassava tubers; B. Cooking grated manioc on large metal plates (photo credits: L. Epelboin)

À la suite d'un foyer de cas de saturnisme dans le bourg de Charvein sur la commune de Mana en 2011 (une fillette présentant une plombémie de 1724 µg/l, entraînant la découverte de 17 autres cas dans l'enquête épidémiologique), plusieurs études ont été réalisées en Guyane et ont retrouvé des niveaux d'imprégnation au plomb très élevés dans certaines populations. Avec un seuil de plombémie ≥ 50 µg/l – seuil de déclaration obligatoire depuis juin 2015, la prévalence du saturnisme est retrouvée à 20,1% (13 fois plus que dans l'Hexagone) chez des enfants de 1 à 6 ans en 2015-2016 ou 25,8% chez les femmes enceintes au CHOG [14, 186]. Les régions les plus concernées sont le haut Oyapock et le haut Maroni [Mosnier *et al.*, communication personnelle]. Des études environnementales suggèrent une exposition multifactorielle en Guyane différente des sources d'exposition habituelles décrites en Europe, possiblement en lien avec la consommation de manioc sous forme de couac (semoule de manioc: probable bioconcentration du plomb dans ce tubercule) (Fig. 56), l'utilisation d'ustensiles de cuisine non conformes, les plombs de chasse dans le gibier, l'utilisation de plombs de lestage pour les filets de pêche ou encore l'abandon de vieilles batteries dans l'environnement. Des études sont toujours en cours pour mieux identifier les facteurs de risques spécifiques. Le plomb est toxique sans seuil évident sur les systèmes nerveux, hématologique et rénal se manifestant par exemple par des troubles du comportement, des neuropathies périphériques, une baisse des performances scolaires, un retard de croissance, des douleurs abdominales ou des troubles digestifs, parfois irréversibles [330].

Le réseau Périnat recommande un dosage de la plombémie aux femmes enceintes présentant des facteurs de risque, aux nouveau-nés et à tout enfant présentant des signes cliniques évocateurs ou exposé à des facteurs de risque supposés [Réseau Périnat Guyane, www.reseauperinatguyane.fr/enfants-vulnerables/plomb-et-mercure]. Un parcours de soins réaliste est en cours d’élaboration [www.guyane.ars.sante.fr/metaux-lourds] prenant en compte l'ampleur du problème, la capacité de prise en charge du système de soins et les difficultés liées à l'isolement géographique.

La prise en charge médicale des enfants avec des plombémies supérieures ou égales à 50 µg/l repose sur la recherche et la correction de carence en vitamine D, calcium et fer (le calcium et le fer inhibant l'absorption du plomb) [162]. En cas de plombémie supérieure ou égale à 450 µg/l un traitement par chélateur est indiqué [159]. Celui-ci n'aura qu'un effet temporaire en cas de constitution d'un stock osseux étant donné la demi-vie de 27 ans du plomb. Une surveillance régulière de la plombémie est recommandée. Une enquête environnementale doit être réalisée ainsi que l’éviction des sources d'intoxication si elles sont identifiées [Réseau Périnat Guyane].

#### Ichtyosarcotoxisme

Loïc Epelboin

**Figure 57:**
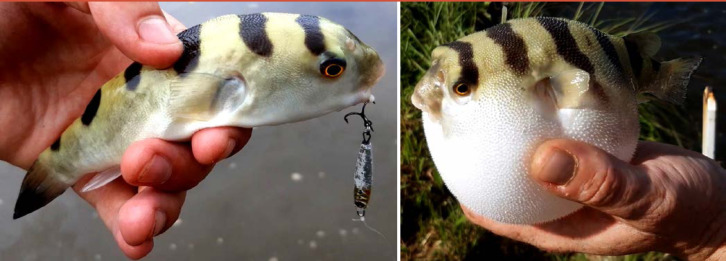
Tétrodon rayé ou poisson-globe *(Colomesus psittacus)* pêché et relâché à Kourou (crédit photos: E. Caussé) *Banded puffer* (Colomesus psittacus) *caught and released in Kourou (photo credits: E. Caussé)*

L'ichtyosarcotoxisme désigne les intoxications alimentaires dues à l'ingestion de poissons ayant accumulé des toxines dans leur chair, leur peau ou leurs viscères. Le plus connu d'entre eux est la ciguatera ou « gratte », rapportée régulièrement aux Antilles et dans les îles françaises de l'océan Pacifique. Il s'agit d'un syndromep olymorphe associé à la ciguatoxine par consommation de chair de poissons de récifs coralliens qui s'intoxiquent par ingestion de micro-algues sur les coraux. Cette intoxication n'est pas rapportée en Guyane. En revanche, quelques cas de tétrodotoxisme ont été signalés de façon sporadique, principalement liés à la consommation de poissons de mer de la famille des Tetraodontidae (par ex: poisson-globe) et Diodontidae (par ex: poisson porc-épic) [149] (Fig. 57). Les signes le plus souvent évoqués sont neurologiques (94,7%) avec paresthésies et ataxie, digestifs (63,2%) avec nausées et vomissements, et généraux (60,5%). Le taux de mortalité est élevé, de l'ordre de 10%.

### Envenimations par la faune sauvage

Rémi Mutricy, Loïc Epelboin, Hatem Kallel

En Guyane, les attaques par la faune sauvage représentent un peu moins de 1% des passages aux urgences [227, 238]. Les plus fréquentes sont, dans l'ordre, les envenimations par hyménoptères, les envenimations par scorpions et les envenimations ophidiennes (serpents). Il faut demander au patient de prendre une photo de l'animal responsable de l'envenimation, voire d'amener le cadavre s'il a été tué (à nuancer selon le type d'espèce, car il est interdit de transporter les espèces protégées, même mortes). Cela permettrait d'identifier plus sûrement l'espèce (en particulier pour les serpents) et le risque d'envenimation et d'adapter la prise en charge.

#### Envenimations par les hyménoptères

La morbidité induite par les hyménoptères (abeilles, guêpes, frelons et fourmis) est de deux ordres. Le premier est lié aux réactions anaphylactiques au venin, et peut donc survenir d'une à quelques piqûres. Le second est induit par la toxicité du venin lui-même qui s'exprime cliniquement en cas d'envenimation massive, soit plusieurs centaines de piqûres. Ce qui fait également le danger de ces envenimations en Guyane est la présence *d'Apis melliferas scutellata* ou abeille africanisée. Celle-ci dépose lors de la piqûre une phéromone (2-heptanone) qui attire les autres abeilles et entraîne une attaque en masse de nombreuses abeilles pouvant poursuivre la victime sur de longues distances (environ 800 m) [127]. Il est donc possible de voir des patients avec de nombreuses piqûres (> 500), et de nombreuses victimes en même temps, comme cela a été rapporté en Guyane [144, 145]. La classification de Schmidt semble la plus adaptée pour décrire le niveau de sévérité [338]. Une nécrose cutanée peut survenir en cas de piqûres multiples (envenimation massive). L'autre conséquence de l'envenimation par hyménoptère est un choc anaphylactoïde dont le traitement est en premier lieu l'adrénaline et une hydratation importante. Lors d'une envenimation importante, il apparaît sur le plan biologique dans un premier temps une insuffisance rénale, une élévation de la troponine et un syndrome inflammatoire, et dans un second temps une rhabdomyolyse et une cytolyse hépatique. En pratique, la première chose à faire, soit sur place, soit aux urgences, est de retirer les dards qui peuvent continuer à libérer le venin. Attention, en le retirant, à ne pas faire pression sur les glandes associées au dard, ce qui a pour effet d'augmenter la quantité de venin injecté.

#### Envenimations scorpioniques

**Figure 58:**
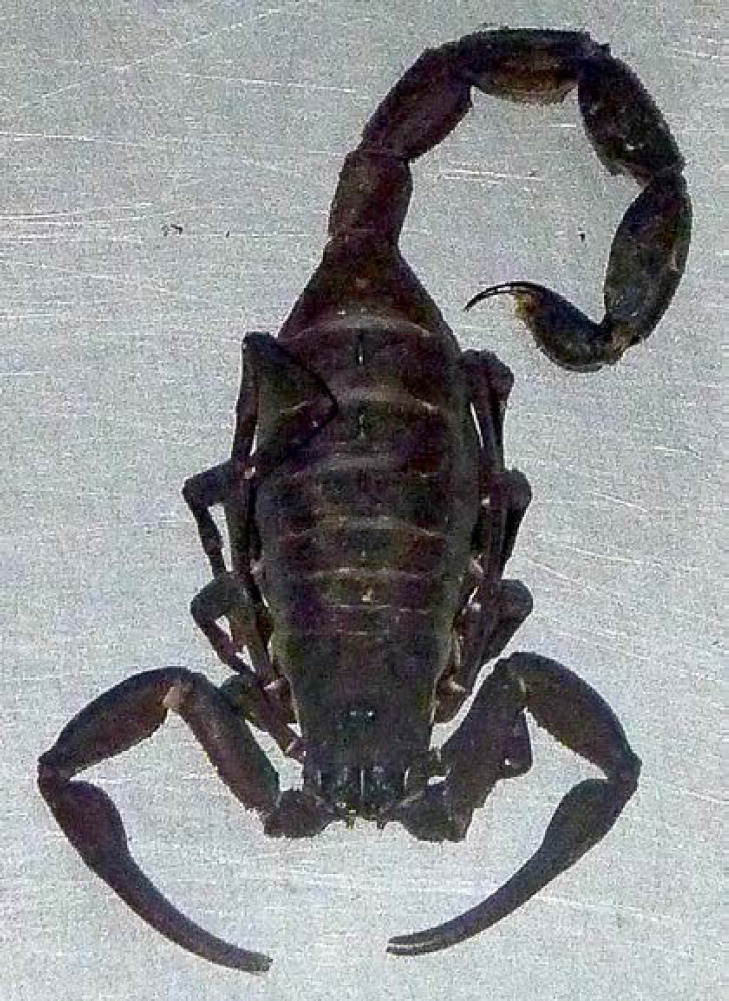
Scorpion « à petites pinces » *Tityus obscurus* dans un bac à douche à Stoupan (crédit photo: L. Epelboin) *Amazonian black scorpion* Tityus obscurus *in a shower basin in Stoupan (photo credit: L. Epelboin)*

Une trentaine d'espèces de scorpions a été identifiée en Guyane [378]. Trois espèces sont responsables d'envenimations dangereuses: *Tityus obscurus, Tityus silvestris* et *Isometrus maculatus* (Fig. 58). Ces scorpions sont reconnaissables à leurs pinces fines. Il y a entre 30 et 40 passages pour envenimation scorpionique par an aux urgences de Cayenne [25]. L'action du venin est à la fois muscarinique et adrénergique. Les enfants sont plus à risque d'envenimations graves [25, 181, 371]. Cependant, seulement deux décès ont été recensés ces 25 dernières années [140, 170]. Le tableau classique en Guyane est celui d'une douleur locale intense avec des paresthésies du membre et une dysarthrie avec hypersalivation régressive dans les 24 à 48 heures. Beaucoup plus rarement, et rapporté plutôt ailleurs qu'en Guyane, lors d'envenimations graves, les patients peuvent présenter une défaillance cardiaque (bradycardie, trouble du rythme ventriculaire, choc cardiogénique), une défaillance respiratoire (œdème aigu du poumon (OAP)), une défaillance neurologique (coma, paralysie) [181]. Les décès ont la plupart du temps lieu lors des chocs cardiogéniques et des OAP. Il n'y a pas d'antivenin disponible en Guyane, le traitement sera symptomatique. Les corticoïdes n'ont pas d'intérêt dans le traitement des envenimations scorpioniques. La lidocaïne en application locale pourrait avoir un effet intéressant sur les douleurs. En cas de défaillance cardiaque, le traitement de référence est la dobutamine. La prazosine semble avoir un intérêt dans cette même indication.

#### Envenimations par les myriapodes

**Figure 59:**
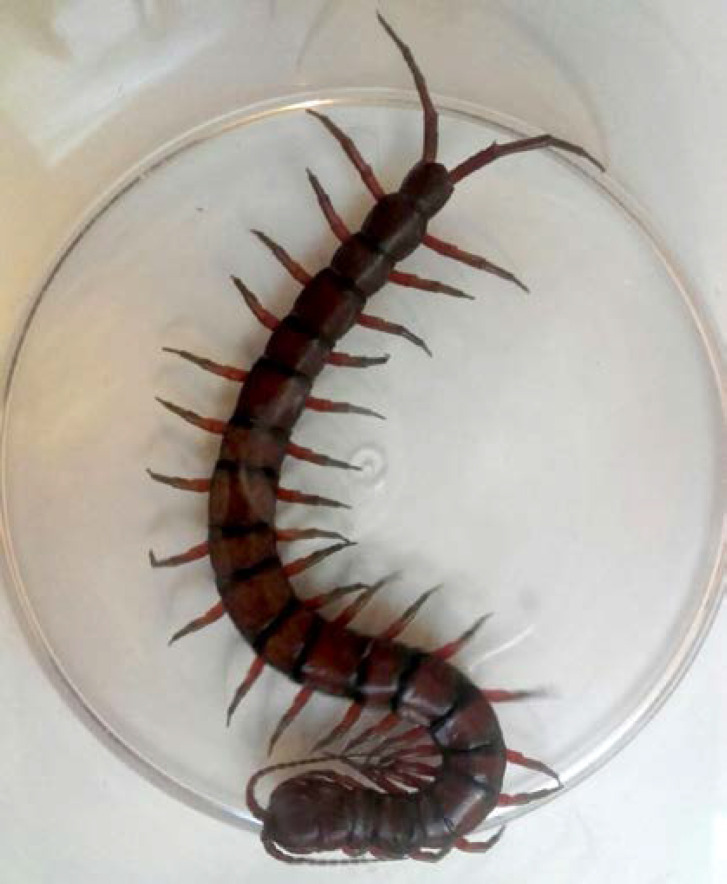
Scolopendre (crédit photo: Y. Epelboin) Scolopendra (photo credit: Y. Epelboin)

Les scolopendres (*Scolopendra* sp.) sont aussi responsables d'envenimations [320] (Fig. 59). À ce jour, il n'y a pas eu de description d'envenimation grave en Guyane. Lors d'une envenimation, la douleur est particulièrement importante. Il convient de désinfecter le site de piqûre, de réaliser un ECG en cas de douleur thoracique, de prendre en charge la douleur (la lidocaïne appliquée localement semble efficace); certains patients notent une amélioration à l'exposition à la chaleur. Une antibioprophylaxie ne semble pas nécessaire.

#### Envenimations par les araignées

**Figure 60:**
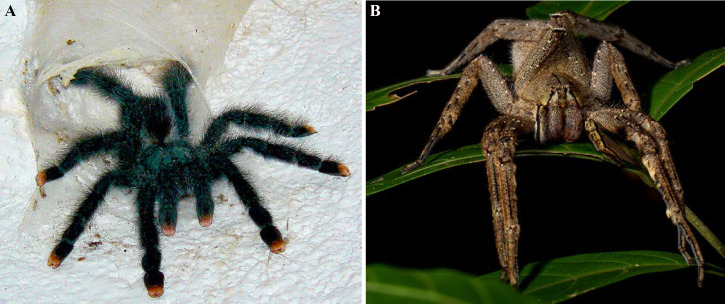
A. Matoutou *(Avicularia avicularia), à* l'entrée de son nid au plafond d'une maison de Stoupan (crédit photo: L. Epelboin); B. *Phoneutria* sp. sur une feuille en forêt (crédit photo: A. Merlin) *A. Pinktoe tarantula* (Avicularia avicularia) *at the entrance of its nest on the ceiling of a house in Stoupan (photo credit:* L. Epelboin); *B.* Phoneutria *sp. on a leaf in the forest (photo credit: A. Merlin)*

Peu de cas d'envenimations par araignées sont rapportés en Guyane. Contrairement à la légende urbaine, ce ne sont pas les mygales, en particulier la fameuse matoutou (*Avicularia avicularia)* qui fait son nid dans le plafond de certaines maisons, qui sont les plus dangereuses, mais les *Phoneutria* sp. [44] (Fig. 60). À notre connaissance, il n'y a pas eu de cas grave diagnostiqué à ce jour en Guyane. Lors d'envenimations, les douleurs sont très importantes. Quant aux envenimations sévères, on retrouve un syndrome muscarinique et adrénergique. Les envenimations sévères sont essentiellement rapportées dans la population pédiatrique.

#### Envenimations par les lépidoptères

**Figure 61:**
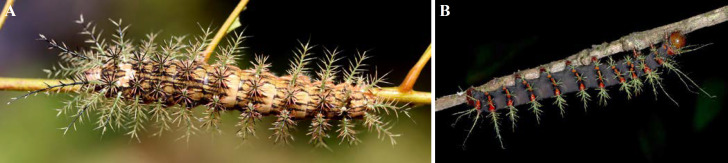
A. Chenille mature de *Lonomia diabolus (ex-Lonomia achelous diabolus)*, La Césarée, Macouria (crédit photo: J.-P. Champenois); B. Chenille de Saturniidae (crédit photo: N. Defaux) *A. Mature caterpillar of* Lonomia diabolus *(formerly* Lonomia achelous diabolus), *La Césarée, Macouria (photo credit: J.-P. Champenois); B. Caterpillar of Saturniidae (photo credit: N. Defaux)*

Les pathologies liées aux lépidoptères sont de deux ordres: celle provoquée par les adultes est appelée lépidoptérisme et inclut la papillonite (cf. supra) et celle provoquée par les chenilles est appelée érucisme [72]. En France, un exemple classique est celui de la chenille processionnaire du pin. En Guyane, les espèces en cause sont variées, appartenant aux genres *Automeris, Dirphia, Megalopyge, Lonomia* et *Hylesia.* Récemment, une envenimation grave par chenilles du genre *Lonomia* a été rapportée [228] (Fig. 61). Ce tableau exceptionnel survient après un contact cutané avec celles-ci. En premier lieu, on observe des douleurs locales à type de brûlure qui peuvent être suivies par des céphalées, des nausées, des douleurs abdominales et des vomissements. Ensuite, dans les envenimations sévères, le tableau typique est une coagulopathie retardée (24-48 h) qui dure longtemps (3 semaines). Habituellement, les envenimations sévères sont retrouvées lors de contacts avec de nombreuses chenilles du genre *Lonomia*. Parfois une seule suffit. Le traitement efficace lors d'apparition de coagulopathie de consommation est l'antivenin. Mais il n'est disponible qu'au Brésil.

#### Envenimations ophidiennes

**Figure 62:**
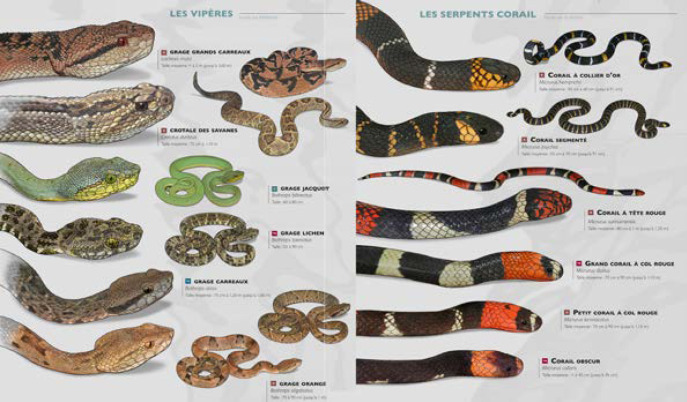
Serpents venimeux et dangereux de Guyane (source Good ID, Cerato et ARS Guyane) Poisonous and dangerous snakes in French Guiana (source: Good ID, Cerato and ARS Guyane)

Parmi la centaine d'espèces de serpents identifiées en Guyane, moins de 15 sont potentiellement dangereuses pour l’être humain (6 espèces de Viperidae et 6 espèces d'Elapidae) (Fig. 62) [61]. Les Viperidae sont les plus fréquemment responsables des morsures, principalement *Bothrops atrox*, localement appelé « grage carreaux » qui est responsable de la très grande majorité des envenimations du fait de sa grande fréquence et son anthropophilie, suivi de *B. oligobalus* (auparavant identifié comme *B. brazili* localement appelé « grage petits carreaux »), *B. bilineatus* (grage jacquot) et *Lachesis muta* (grage grands carreaux ou bush master) [253]. Les morsures d'autres espèces ont été décrites mais restent peu courantes: *Crotalus durissus*, serpent à sonnette sud-américain, est devenu rare, et les morsures de *Micrurus* sp., serpent corail, appartenant à la famille des Elapidae, sont exceptionnelles [Heckmann X, Marty C, Starace F, Louembé JD, Larréché S. Envenimation par *Micrurus psyches* en Guyane française. Bull Soc Pathol Exot. 2017 Oct;110(4):276-280. doi.org/10.1007/s13149-017-0567-9]. La symptomatologie classique observée lors d'une envenimation vipérine comprend des signes locaux au niveau du site de la morsure (douleur, œdème, saignement) et, dans les envenimations sévères, nécrose cutanée, des signes généraux (défaillance hémodynamique, défaillance rénale, hémorragies systémiques) [253]. Les venins du genre *Bothrops* sont hémorragiques, ils endommagent l'endothélium vasculaire et consomment les facteurs de coagulation selon un mécanisme connu sous le nom de coagulopathie de consommation induite par le venin. Il y a en moyenne 100 passages par an aux urgences des hôpitaux de Guyane pour envenimation par morsure de serpent: 30 à 35 au CHC, 10 au CHK et 40 à 50 au CHOG [165, 173, 253]. Parmi les patients envenimés, 15 à 32% ont des complications fonctionnelles locales telles que nécrose des tissus, abcès et dermohypodermites nécessitant des prises en charge à type de nécrosectomie, drainage d'abcès ou de phlegmon, voire amputation de l'extrémité d'un membre [253, 310]. Lors des complications infectieuses, les bactéries les plus souvent retrouvées sont *A. hydrophila* et *M. morganii* [309]. L'antibiothérapie prophylactique ne semble pas indiquée car une majorité d'envenimations ne s'infectent pas (environ 30% d'infection). L'antibiothérapie curative adaptée à la documentation microbiologique (espèce bactérienne et antibiogramme) est indiquée en cas d'infection locale avérée et dans les envenimations sévères. En l'absence d'antivenin, la mortalité peut être élevée en cas de morsure de grage [254]. Cependant, depuis 2016, seuls 3 décès ont été rapportés en Guyane. Le traitement repose sur l'antivenin et les traitements symptomatiques [190]. L'antivenin utilisé est l'Antivipmyn^©^ Tri, qui semble efficace; une comparaison avec d'autres antivenins disponibles serait néanmoins nécessaire.

#### Envenimations par les raies

**Figure 63:**
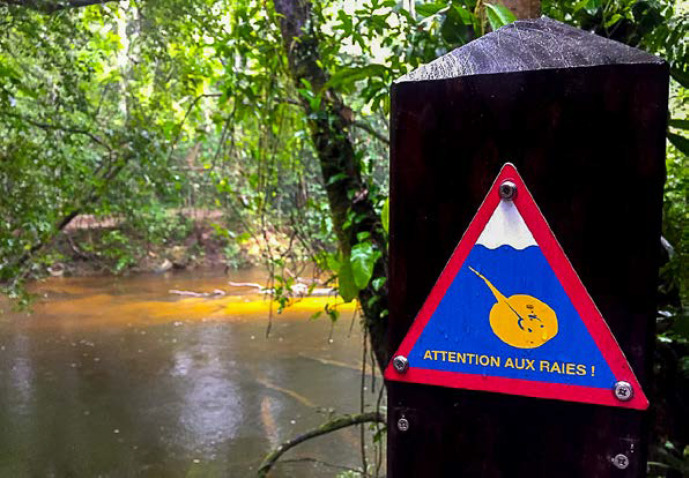
Panneau avertissant du risque de présence de raies au niveau de la crique Tonnégrande, au bout du sentier du Bagne des Annamites (crédit photo: L. Epelboin) Sign warning of the presence of stingrays near the Tonnégrande creek, at the end of Annamites Prison footpath (photo credit: L. Epelboin)

Enfin, il existe aussi en Guyane des envenimations par piqûre de raie (*Potamotrigon* sp.) (Fig. 63) ou de poisson-chat. Les personnes se font piquer en marchant sur les raies qui sont généralement posées au fond des rivières sur les fonds sablonneux. Les piqûres sont très algiques; en dehors des traitements antalgiques allant jusqu'au palier 3, il convient de placer la zone piquée dans de l'eau à une température élevée, mais restant supportable, c'est-à-dire inférieure à 50 °C, le venin étant inactivé à partir de 42 °C, jusqu’à disparition des douleurs (30 à 90 minutes). La réalisation d'anesthésie locorégionale semble efficace [370]. Suite à ces envenimations, il existe un risque de surinfection aux germes hydriques, en particulier *Aeromonas hydrophila* [92] (Fig. 35B).

### Pathologies tropicales non infectieuses diverses

#### Hémoglobinopathies

Lindsay Osei, Narcisse Elenga, Loïc Epelboin

La drépanocytose est une maladie génétique de l'hémoglobine à mode de transmission autosomique récessive. Elle est l'une des maladies génétiques les plus répandues dans le monde, essentiellement chez les populations originaires d'Afrique intertropicale. En Guyane, du fait d'une population majoritairement afro-caribéenne, c'est une priorité de santé publique comme dans les autres territoires ultramarins. La prise en charge de la drépanocytose en Guyane s'est progressivement développée avec la mise en place du dépistage néonatal en 1992, la création d'un réseau de professionnels de santé en 2008 et la mise en place en 2012, à Saint-Laurent-du-Maroni, d'activités d’éducation thérapeutique par l'infirmière coordinatrice de la drépanocytose. Ces différentes structures travaillent en étroite collaboration avec les deux associations des usagers (DrépaGuyane et Femmes en devenir). L'ouverture du Centre intégré de la drépanocytose (CID) au CHC en 2014 s'inscrit dans cette dynamique d'amélioration de la qualité de la prise en charge des patients drépanocytaires en Guyane. L'incidence des syndromes drépanocytaires majeurs à la naissance était de 1/227 en Guyane sur la période 1992-2010 (vs 1/784 dans l'Hexagone, 1/4 551 à la Réunion, 1/633 à Mayotte, 1/343 en Martinique, et 1/297 en Guadeloupe) [125]. La fréquence des porteurs du trait drépanocytaire est de 10% en Guyane, 5,2% pour l'ensemble des DOM *versus* 2,7% pour l'Hexagone pour la période 2006-2010. La file active des patients suivis en 2022 est pour le CID du CHC de 285 enfants et 394 patients adultes. Au CHOG, 300 enfants et 200 adultes ont été suivis entre 2011 et 2022. En 2022, on estime la population de patients drépanocytaires en Guyane entre 1 200 et 2 000 personnes. Une étude récente a montré que les haplotypes de l'hémoglobine S fréquents en Guyane sont les haplotypes Bénin (65,9%), Bantu (20,5%), Sénégal (7%), Cameroun (4%), Arabo-indien (1%) et Atypique (6%) [345]. Une étude a porté sur les 58 patients adultes drépanocytaires hospitalisés au CHC entre janvier 2010 et juillet 2011 [312]. Le sex-ratio H/F était de 0,75 et l’âge moyen de 28,5 ans; 67% présentaient un phénotype SS, 10% Sβ thalassémique (Sβ° et Sβ+) et 23% SC. La crise vaso-occlusive (CVO) représentait 86% des motifs d'hospitalisation et les infections 16%. Enfin, 80% ont nécessité la mise en place d'une analgésie auto-contrôlée (PCA, Patient-controlled analgesia) de morphine. Entre janvier 2010 et décembre 2016, 1739 passages aux urgences ont été enregistrés chez 384 patients suivis pour drépanocytose, ayant abouti à 856 hospitalisations (49,2%) [284]. La baisse de la température et la diminution de l'humidité étaient deux facteurs indépendants associés à une augmentation des cas de CVO (p = 0,0128 et p = 0,0004, respectivement). Avec ou sans passage par les urgences, 2104 hospitalisations ont été enregistrées pour CVO sévère pour 326 patients. Le seul facteur associé aux CVO sévères était les épidémies de grippe.

#### Béribéri

Frédégonde About, Loïc Epelboin

Le béribéri est une maladie grave, potentiellement mortelle, qui résulte d'une carence en vitamine B1 (thiamine). Le béribéri, faiblesse en sri-lankais, a été initialement décrit chez des peuples d'Asie qui se nourrissaient exclusivement de riz blanc décortiqué, tandis que la cuticule du riz contient de la vitamine B1 [248]. Une carence en vitamine B1 peut être aussi secondaire à une intoxication alcoolique chronique. La maladie se présente sous deux formes cliniques différentes: une forme dite sèche avec une atteinte neurologique prédominante (béribéri sec) et une forme humide avec atteinte cardiaque prédominante (béribéri humide) pouvant se présenter sous une forme fulminante avec insuffisance cardiaque aiguë et hyperlactatémie (shoshin béribéri).

L'atteinte neurologique peut se présenter sur forme d'une atteinte périphérique (polynévrite sensitivo-motrice) ou centrale (encéphalopathie de Gayet-Wernicke) qui peut s'associer à des troubles de la vigilance, un syndrome confusionnel, des troubles de l’équilibre ou encore une paralysie oculomotrice. En l'absence de supplémentation en vitamine B1, cette atteinte neurologique peut évoluer vers une amnésie antérograde avec désorientation, encore appelée syndrome de Korsakoff.

En Guyane, le béribéri était une maladie classiquement rapportée au temps du bagne, et avait disparu depuis. En 2013 une épidémie de béribéri est survenue chez des orpailleurs non loin de Maripasoula où 42 personnes ont consulté pour une symptomatologie de cardiomyopathie [248]. Cette épidémie était probablement d'origine carentielle par pauvreté des apports alimentaires chez ces personnes vivant en forêt profonde de façon prolongée. Les principaux symptômes retrouvés étaient la dyspnée (60%), les œdèmes (79%), les signes d'insuffisance cardiaque droite avec turgescence jugulaire (63%) et le reflux hépato-jugulaire (42%). Parmi ces 42 personnes, 67% avaient une forme humide, 31% avaient une forme mixte, et 1 avait un shoshin béribéri. Le diagnostic de carence en vitamine B1 peut être confirmé par un dosage sanguin de l'activité transcétolase érythrocytaire. Toutefois, les complications de la carence en vitamine B1 étant graves, ce dosage, rarement réalisable en communes isolées, ne doit pas retarder le traitement qui consiste en une supplémentation en vitamine B1 par voie orale, intraveineuse, ou encore intramusculaire [163]. L’évolution sous traitement est généralement rapidement favorable après supplémentation. La prévention du béribéri repose sur une alimentation diversifiée et équilibrée pour éviter la carence en vitamine B1.

#### Alphagal

Loïc Epelboin

**Figure 64:**
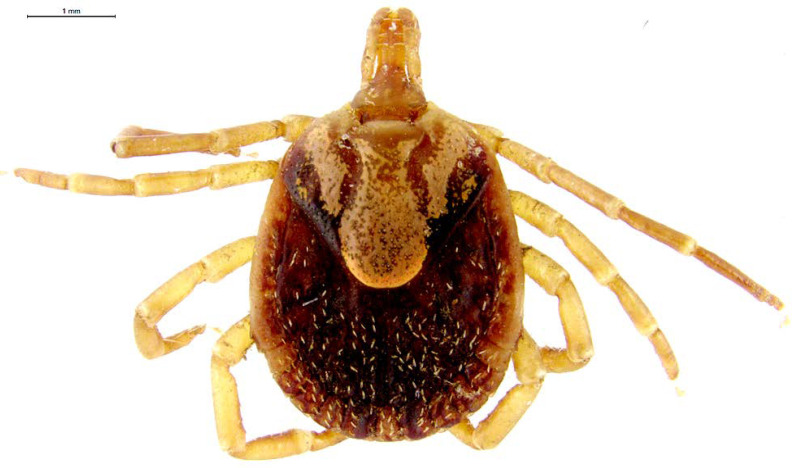
Femelle adulte de la tique *Amblyomma cajennense* (crédit photo: F. Binetruy, O. Duron) *Adult female of the* Amblyomma cajennense *tick (photo credit: F. Binetruy, O. Duron)*

La dernière décennie a vu l’émergence d'un nouveau type d'allergie alimentaire survenant de manière retardée quelques heures après l'ingestion de viande de mammifères. Cette allergie est médiée par un sucre, le Galactose-alpha-1,3-galactose qui a donné son nom à la maladie, l'alphagal. Elle a été décrite pour la première fois en 2009 aux États-Unis, puis elle a été rapportée principalement en Australie et dans plusieurs pays d'Europe [65, 66]. Une étude menée en Guyane a identifié 11 de ces patients entre 2017 et 2019, la majorité étant originaire de l'Hexagone et âgée entre 30 et 60 ans [122]. La plupart (9/11) présentaient des symptômes digestifs, 4/11 avaient des réactions cutanées et 3/11 des réactions respiratoires avec un œdème de Quincke. Le délai entre ingestion et réaction variait entre 1 h 30 et 6 h. Les viandes incriminées étaient le plus souvent le bœuf et le porc, le régime d’éviction faisait disparaître les symptômes. Tous les patients étaient régulièrement exposés aux morsures de tiques, probablement *Amblyomma cajennense* (Fig. 64), proche de la tique étoilée *Amblyomma americanum* retrouvée dans les cas nord-américains. Le diagnostic repose à la fois sur les tests cutanés et le dosage des IgE anti-alphagal et IgE anti-viande de mammifères spécifiques (porc, bœuf, mouton) épargnant la volaille et les poissons. Depuis cette étude, de nombreux cas ont été diagnostiqués en Guyane, majoritairement chez des Français de l'Hexagone fréquentant la forêt de façon répétée, souvent pour des raisons professionnelles, avec piqûres régulières de tiques. La rareté des cas dans les autres communautés de Guyane interroge, faisant évoquer une possible protection des personnes porteuses du groupe sanguin B, plus fréquente chez les Afro-Caribéens, mais absente théoriquement dans les autres communautés.

## Phénomènes Spécifiques À La Guyane

### Bodypacking

Alexis Fremery, Timothée Bonifay

**Figure 65:**
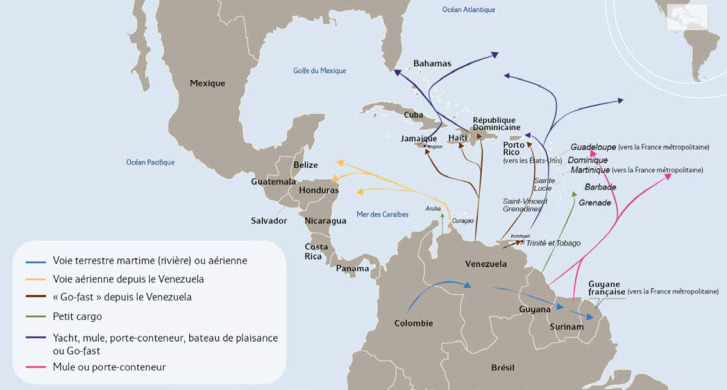
Carte des principales voies du narcotrafic depuis l'Amérique du Sud (source: OFDT, 2016) *Map of the major drug trafficking routes from South America (source:* OFDT, 2016)

La Guyane constitue une plaque tournante du trafic de cocaïne entre les Amériques et l'Europe (Fig. 65). L'Office anti-stupéfiants (OFAST) et l'Observatoire français des drogues et toxicomanies (OFDT) estiment que 15% de la cocaïne consommée en France proviendrait de Guyane et serait acheminée quotidiennement par 20 à 30 passeurs quotidiens (mules) sur la ligne aérienne Cayenne-Paris. Au cours des 10 dernières années, la quantité de cocaïne saisie a été multipliée par 15 en Guyane et le nombre de mules hospitalisées sur l'hôpital de Cayenne multiplié par 6, passant de 20 personnes en 2011 à plus de 120 en 2019 [375]. En a résulté une augmentation du nombre d'incarcérations pour trafic de stupéfiants, motif d'emprisonnement en 2021 de 20% des hommes et 80% des femmes au Centre pénitentiaire de Guyane [Bonifay, données non publiées].

**Figure 66:**
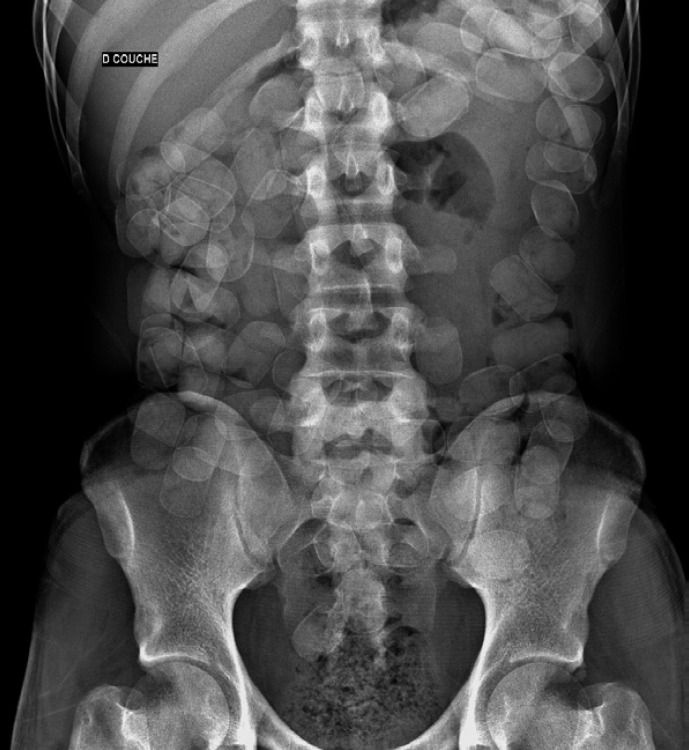
Cliché d'abdomen sans préparation de face montrant de nombreux corps étrangers (ovules de cocaïne ou « boulettes ») opaques dans l'ensemble du cadre colique aux urgences de Cayenne (crédit photo: M. Zappa) Plain abdominal film showing numerous opaque foreign bodies (cocaine eggs or “pellets”) throughout the colonic frame in the Cayenne Hospital emergency room (photo credit: M. Zappa)

Le transport de cocaïne prend deux formes: le transport externe et l'ingestion d'ovules de cocaïne (bodypacking). Sur le territoire guyanais les ovules utilisés proviennent essentiellement du Suriname et sont principalement thermo-soudés, de qualité semi-industrielle (de type 3) et sont à faible risque de rupture. Il convient de noter l'implantation récente de réseaux de fabrication locaux d'ovules de qualité intermédiaire (de type 2) qui présentent un risque plus élevé de rupture (emballage de moins bonne qualité). Chaque boulette avalée contient entre 8 et 10 grammes de cocaïne, soit 8 fois la dose létale potentielle pour le porteur. Certains ovules plus gros, allant de 50 à 200 g, sont insérés par voie rectale ou vaginale. Un bodypacker peut ainsi transporter jusqu’à 1 kg de cocaïne. Sur le plan médical, les deux principaux risques liés à la pratique du bodypacking sont l'intoxication aiguë sur rupture de boulette, avec un risque de décès élevé, et les complications mécaniques, notamment l'occlusion digestive. Au cours des 10 dernières années, et malgré l'augmentation croissante du nombre de bodypackers pris en charge en Guyane, peu de complications ont été recensées. Sur 731 « mules » prises en charge entre 2010 et 2019 sur l'hôpital de Cayenne, seules 3 (0,4%) ont présenté des complications sévères [37, 294]. Malgré tout, le risque de décès reste une réalité en cas de rupture d'ovules. En 2022 en Guyane, 4 personnes sont décédées en milieu extra-hospitalier tandis qu'un patient hospitalisé a été pris en charge chirurgicalement en urgence et a survécu. Ce risque est directement lié à la qualité de l'emballage des ovules. Devant l'augmentation de la fabrication « maison » d'ovules, les praticiens doivent rester vigilants et ne pas sous-évaluer le risque de rupture. Actuellement, la prise en charge du bodypacking non compliqué consiste en l’évacuation des boulettes, une surveillance hospitalière systématique et un contrôle recommandé par cliché radiographique plus ou moins simple par scanner low-dose, pré-et post-hospitalisation (Fig. 66). Une prise en charge endoscopique est recommandée en cas de retard d’évacuation des ovules: ainsi entre 2010 et 2019, 65 patients en ont bénéficié, soit 9% des bodypackers hospitalisés [37, 304]. Il faut noter une diversification du profil des mules: un plus grand nombre de femmes enceintes ou de personnes âgées sont recrutées, même si le public principal reste de jeunes Guyanais du fleuve Maroni [37]. Le bodypacking est plus que jamais un enjeu majeur de santé publique en Amérique du Sud et particulièrement en Guyane.

### Bouglous

Timothée Bonifay

**Figure 67:**
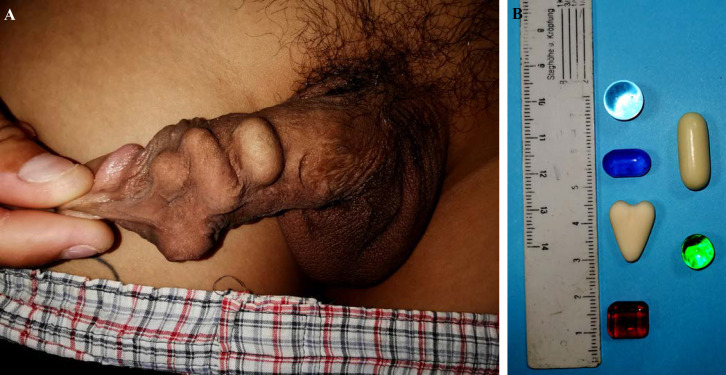
A. Plusieurs « bouglous » ou « dominos » sous-cutanés chez un patient incarcéré au Centre pénitentiaire de Guyane; B. Bouglous après extraction (crédit photos: T. Bonifay) A. Several subcutaneous “bouglous” or “dominoes” in a patient incarcerated at the French Guiana Penitentiary Centre; B. Bouglous after extraction (photo credits: T. Bonifay)

Les implants ou nodules péniens artificiels (NPA) sont des objets insérés sous la peau du pénis (Fig. 67). Bien que la littérature sur le sujet soit rare, cette pratique est décrite depuis longtemps, comme dans le Kama Sutra. Ces NPA sont appelés yakuza, bolitas (Philippines), tancho's nodules (Thaïlande), ruJu (Taïwan), penis marbles, penile beading, dolphins (Serbie) et bouglous ou dominos en Guyane. Le terme bouglou correspond à la traduction phonétique du terme *boegroe* utilisé au Suriname, signifiant « boulette » en créole surinamais [198, 259]. Il s'agit de petites boules sphériques, ovoïdes ou oblongues obtenues en chauffant à la flamme le plastique du manche de brosse à dents ou de pièces de domino préalablement râpé. Les NPA sont ensuite insérés sous la peau de la verge, après incision avec un objet contondant de fortune, comme le couvercle d'une boîte de conserve. Quoique maintenant répandue dans la population générale, cette pratique reste liée à l'univers carcéral. En 2020, sur 557 hommes nouvellement incarcérés dans l'année, 38,8% avaient déclaré avoir au moins 1 bouglou (n = 168/432). Parmi eux, 42,2% en avaient 5 ou plus et 5,4% en avaient plus de 15 [Bonifay, données non publiées]. Au total, 30% les avaient posés hors du centre pénitentiaire contre 16% en 2014 [259]. Cette pratique semble se diffuser de plus en plus dans la population générale, en particulier chez les hommes jeunes.

Deux problématiques d'ordre médical peuvent être soulevées: un risque aigu en lien avec la pose (infection cutanée bactérienne, paraphimosis infectieux, hémorragie locale, notamment) et un risque au niveau de la santé publique [198]. En effet, 52% des hommes concernés disaient avoir des difficultés à utiliser les préservatifs et 36% déclaraient en utiliser deux l'un sur l'autre afin d’éviter les déchirures [283]. Devant la forte incidence des IST en Guyane, 1,3% de PVVIH en population générale et 3,8% en milieu carcéral, les enjeux en matière de santé sexuelle sont importants.

### Phytothérapies de Guyane

Marc-Alexandre Tareau

La Guyane est largement connue pour son incroyable diversité culturelle, faisant cohabiter des communautés amérindiennes, businenges, créoles et migrantes dans un improbable mélange de pratiques linguistiques et culturelles. À cette riche diversité humaine se superpose une incroyable biodiversité végétale, faisant de ce territoire un lieu d'exception en termes de pratiques ethnobotaniques. Ainsi, 356 espèces à usages médicinaux sont employées sur le littoral guyanais uniquement [355] et plus de 600 en comptant les pratiques des populations de l'intérieur forestier [133, 153]. Dans cette vaste flore médicinale, il faut noter que nombreuses sont les panacées pantropicales exotiques et globalisées, souvent aussi comestibles (près d'un quart des espèces), et dont les usages sont transverses à de nombreux groupes culturels [357]. Cette transversalité est d'ailleurs souvent renforcée par un effet de visualisation médiatique dont bénéficient certaines espèces *via* les réseaux sociaux. Cependant, des spécificités apparaissent, avec des plantes qui détiennent une valeur symbolique et culturelle forte au sein de certaines communautés en s'inscrivant dans des schémas ethnomédicinaux particuliers, à l'image du tropisme pour les plantes et les préparations amères (en particulier les alcoolatures, appelées *anmè* chez les Créoles antillo-guyanais et *bita* chez les Businenges (Fig. 53); chez les Afro-descendants, l'amertume détient un rôle thérapeutique qui s'imbrique dans la lecture humorale du corps). Mentionnons également le fait qu'il s'agit le plus souvent de médecines holistiques qui prennent en compte des explications socio-cosmiques dans la survenue de la maladie [59, 355]. En réponse à cela, le volet médico-magique est souvent très important dans ces médecines qui mobilisent toute une panoplie de plantes propitiatoires et expiatoires employées à titre curatif ou à titre préventif contre les mauvais esprits, les attaques sorcellaires ou encore le « mauvais œil ».

Les plantes auto-cultivées constituent le mode d'approvisionnement le plus important sur l'ensemble du territoire, même si la cueillette en milieu sauvage demeure également un mode d'accès privilégié aux plantes médicinales, en particulier dans l'ouest guyanais où elle semble favorisée par l'importance des plantes rudérales dans les bains d'hygiène intime féminins [356]. Comme observé ailleurs, les femmes occupent une place centrale dans la transmission des connaissances autour des plantes médicinales. Sur le littoral guyanais, celle-ci reste très essentiellement intra-communautaire et intergénérationnelle, correspondant principalement à une transmission de type vertical [358]. Néanmoins, l'apport des migrants sur les pharmacopées locales (à travers, notamment, la relocalisation d'espèces et d'usages exogènes) n'est pas négligeable et contribue tout de même à une interculturalisation progressive des pratiques [355]. L'influence des Caraïbes et des pays limitrophes, d'où provient une grande partie de la population guyanaise, est notamment très importante, montrant le rôle constructif que joue la migration sur l’évolution de la composition des pharmacopées [355, 358]. D'ailleurs, les migrants récents, pour qui la phytothérapie constitue encore une alternative peu coûteuse à la biomédecine et un marqueur identitaire fort, contribuent très probablement au fait que l'usage des plantes médicinales se maintienne nettement dans les villes guyanaises [359].

### Problématique du suicide chez les peuples autochtones

Loïc Epelboin, Marc-Alexandre Tareau

**Figure 68:**
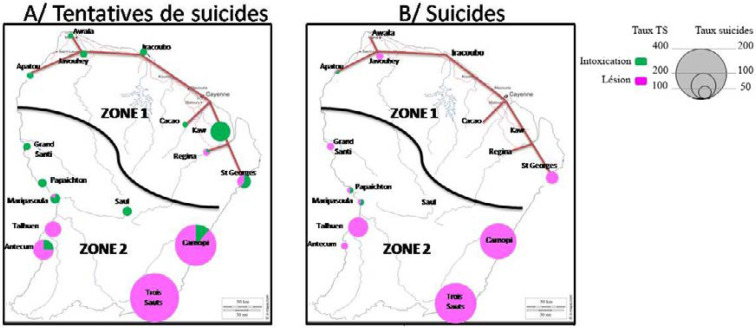
Nombre de cas de suicides et tentatives de suicide par localisation en Guyane, 2007-2016 [154] Number of cases of suicides and suicide attempts by location in French Guiana, 2007-2016 [154]

La Guyane connaît depuis les années 2000 des suicides en série chez les jeunes Amérindiens, principalement chez les Wayãpis du haut Oyapock, les Wayanas du haut Maroni, et les Teko présents sur les rives de ces deux fleuves (Fig. 68). Le taux de suicide parmi ces jeunes serait entre 8 et 20 fois plus élevé que celui relevé en Guyane et dans l'Hexagone [15, 154, 275]. Les peuples amérindiens côtiers (Kaliña, Palikur et Arawak) sont également concernés, mais à moindre échelle. Une étude réalisée à partir de la base de données des CDPS sur la période 2007 à 2016 a montré que les taux de suicide les plus élevés étaient observés à Camopi et à Trois Sauts (118 et 78 pour 100 000 habitants, respectivement) [154]. Les méthodes utilisées étaient principalement la pendaison (65%), l'intoxication au paraquat (22%), suivis du suicide par armes à feu (13%). Le taux de réussite des suicides allait de 8,3 à 19,7% selon la zone étudiée. Le taux de létalité de l'intoxication au paraquat était de 26%. La tranche d’âge 10-19 ans représentait 56,7% des suicides réussis dans les communes du haut Oyapock et du haut Maroni. Aucune différence significative n’était retrouvée entre les genres masculin et féminin.

En 2015, une mission parlementaire a fait 37 propositions « pour enrayer ces drames et créer les conditions d'un mieux-être » [15]. Les causes évoquées de ce dramatique phénomène sont multifactorielles: isolement géographique, désœuvrement et absence de perspectives, raisons économiques et sociales, causes sanitaires, intrafamiliales, identitaires et culturelles. D'autres phénomènes plus sensibles et débattus sont évoqués, tels que les violences intrafamiliales, l'alcoolisme aigu et chronique, les grossesses précoces, les abus sexuels dans l'enfance, les addictions aux drogues (alcool, tabac, cannabis, cocaïne, médicaments psychotropes…), le rôle des églises, le poids des contraintes sociales et des traditions, la pauvreté, le manque de perspectives d'avenir, notamment économique, les gérontocraties et les conséquences de la colonisation [15, 275]. La mission a identifié le trouble identitaire qui mine ces populations, et notamment les jeunes, tiraillés entre le monde dans lequel ont vécu leurs grands-parents et le monde moderne dans lequel ils sont baignés durant leur scolarisation au collège et au lycée sur le littoral. Le rapport ajoute que ce trouble est amplifié par un fort sentiment d'humiliation, lié aux jugements stigmatisants et aux commentaires désobligeants dont les Amérindiens sont régulièrement victimes, et au fort sentiment de frustration et d'abandon, lié aux conditions très dégradées de l'accès au droit et aux services publics les plus élémentaires, lesquels font très clairement défaut sur le haut des fleuves.

### Crises de baklou

Loïc Epelboin, Marc-Alexandre Tareau

Depuis une dizaine d'années, survient régulièrement sur le Maroni un phénomène dont la nouvelle est diffusée par les médias locaux, que l'on nomme les « crises de baklou ou baclou » Ces événements, qui surviennent en général dans les établissements scolaires du Maroni, sont toujours très impressionnants à vivre tant par les élèves, souvent issus de la commune, que par les enseignants et les soignants, originaires du littoral ou de l'Hexagone. Il s'agit de sortes de « crises d'hystérie collective » au cours desquelles des adolescentes, souvent issues des communautés businenges, se mettent soudainement à trembler, crier, et se jettent par terre, généralement suivies par plusieurs camarades de lycée, qui tombent les unes après les autres « dans une transe incontrôlable, possédées » [135]. Ces dernières années, de telles crises sont survenues dans des collèges et lycées de Maripasoula, Saint-Laurent-du-Maroni et Papaïchton. Dans la mythologie créole, le baklou est un mauvais esprit, incarné sous la forme d'un petit animal ou d'une sorte de « gnome visqueux » à forme humanoïde, et réputé de petite taille. Il alimente beaucoup de contes guyanais. On dit par exemple qu'il rend des services à son propriétaire (succès dans les affaires, règlement de comptes…) en échange d’être nourri de chair animale, voire humaine dans les cas les plus extrêmes. Il est généralement conçu à la période de Pâques, pendant laquelle un individu doit couver un œuf sous son bras pendant sept jours et réciter des incantations spécifiques. Ailleurs, comme chez les Businenges, on le considère comme un esprit malfaisant capable de posséder une personne, dans le cadre de rites dédiés ou à l'occasion d'attaques sorcellaires spontanées. Les jeunes femmes possédées sont généralement conduites au CDPS ou au CHOG, et se rétablissent en quelques heures. Ces événements marquent souvent les esprits, conduisant des élèves à refuser de retourner dans leur établissement, le considérant comme hanté. L'un des épisodes médiatisés en 2018 au lycée Lumina-Sophie à Saint-Laurent-du-Maroni aurait été attribué par certains à la prise par les adolescentes d'une drogue appelée « sukru ». Cette drogue, signifiant « sucre » en nenge tongo, la langue du fleuve, qui circulerait le long des fleuves frontières, contiendrait du MDMA (3,4-méthylènedioxyméthamphétamine) sous forme cristal [290,_291].

## Obstétrique

Gabriel Carles

### Épidémiologie des grossesses

Dans ce territoire français en pleine croissance démographique (+ 3,8% par an), la prise en charge des grossesses représente une part importante des besoins médicaux [236]. La Guyane est un carrefour de rencontres de plusieurs ethnies, chacune d'entre elles ayant des spécificités médicales et culturelles nécessitant une adaptation constante de la part des personnels de santé. Seul pays de la région offrant une médecine moderne, la Guyane représente un attrait pour les populations des pays avoisinants et de la Caraïbe, ce qui entraîne un taux élevé de grossesses pathologiques. Le taux de fécondité est de 3,6 enfants par femme contre 1,9 dans l'Hexagone, et une augmentation de 30% des naissances a été observée entre 2013 et 2019. Dans cette population où 60% des femmes enceintes sont d'origine étrangère, vont se concentrer les difficultés de suivi de grossesse en raison des situations de précarité, de faible couverture sociale et de difficultés d'accès aux soins. Seules 45% des femmes enceintes ont un suivi de grossesse conforme aux recommandations. Il en résulte une mortalité périnatale 2 fois plus élevée que dans l'Hexagone et une mortalité maternelle 5 fois plus importante. Les 3 hôpitaux du territoire sont situés sur le littoral et les patientes des communes isolées doivent y être transférées pour l'accouchement. Après avoir été très élevé dans les années 80, le taux d'accouchement à domicile se situe aujourd'hui autour de 1% malgré les difficultés de transport. Une particularité du territoire est le taux très élevé de grossesses chez les mineures, 8 fois celui observé dans l'Hexagone, secondaire à un processus de déculturation des populations du fleuve associé à un grand nombre d'abus sexuels. Par ailleurs, étant donné le faible impact de la contraception dans certaines communautés, les très grandes multipares ne sont pas rares.

### Grossesses pathologiques

#### Complications non infectieuses de la grossesse

Les taux d'HTA gravidique et de prééclampsie sont trois fois plus élevés que dans l'Hexagone, notamment dans les populations afro-descendantes (15 éclampsies par an au CHOG).

Le taux de prématurité est 2 fois plus important que dans l'Hexagone en raison de nombreux facteurs de risque: défaut de suivi de grossesse, forte prévalence de la prééclampsie, grossesses adolescentes, taux élevé d'IST.

Depuis quelques années, on constate une inflation de l'obésité et du diabète gestationnel secondaires aux changements des habitudes alimentaires avec leurs répercussions sur la grossesse.

La meilleure prise en charge des enfants drépanocytaires homozygotes entraîne une augmentation régulière du nombre de grossesses dans cette population.

Une pathologie importante est représentée par l'anémie gravidique qui touche 40% des gestantes dans l'ouest guyanais [211]. Il s'agit le plus souvent d'anémies ferriprives, parfois très sévères, secondaires à l'ingestion par les femmes enceintes de pemba, qui est un puissant chélateur du fer (Fig. 68).

Les femmes enceintes de Guyane sont confrontées à plusieurs intoxications aux métaux lourds:

- Saturnisme chez 34% des patientes des fleuves frontières en cas d'alimentation prédominante par le manioc et ses dérivés;
- Intoxication par le mercure chez les populations amérindiennes du haut Maroni et haut Oyapock secondaire à l'orpaillage clandestin;
- Intoxication par l'aluminium chez les patientes géophages.

### Complications infectieuses de la grossesse

La pathologie infectieuse est riche en Guyane et n’épargne pas les femmes enceintes:

- VIH: région d'endémie avec un taux stable depuis 10 ans autour de 1,2%.
- IST: prévalence très importante des infections à chlamydiae et à gonocoque dont le dépistage doit être systématique pendant la grossesse. Explosion récente des cas de syphilis chez les femmes enceintes dans l'ouest, grande pourvoyeuse de mort fœtale *in utero.* On observe également un taux très élevé de patientes porteuses d'HPV dont les conséquences sont retrouvées dans la forte prévalence du cancer du col de l'utérus dans ce département.
- Arboviroses:
  ° Les épidémies récurrentes de dengue ont pour conséquences sur les grossesses une augmentation de la prématurité, des morts fœtales et des hémorragies de la délivrance [22];
  ° L’épidémie de chikungunya en 2014-2015 s'est accompagnée d'un faible risque d'encéphalopathie néonatale;
  ° Plus d'un millier de femmes enceintes ont été contaminées par le virus Zika lors de l’épidémie de 2016 avec parfois de graves conséquences: malformations cérébrales et retard psychomoteur à long terme [298].
- HTLV-1: la Guyane est une des principales zones d'endémie du HTLV-1, dépisté chez toutes les femmes enceintes afin d'inciter à éviter l'allaitement maternel, voie principale de contamination.
- Paludisme: cause importante d'avortements et de morts fœtales dans les années 80-90, celui-ci est en voie de disparition dans le territoire.
- D'autres infections susceptibles d'avoir des conséquences sur la grossesse sont observées en Guyane, notamment la leptospirose et la fièvre Q.

Pour répondre à tous ces défis, les hôpitaux guyanais bénéficient d'un équipement adéquat mais ils sont souvent débordés par l'ampleur des grossesses pathologiques et l'augmentation constante du nombre de naissances. En dehors du littoral, les centres de santé répartis sur le territoire assurent souvent la surveillance des grossesses à la place d'une PMI surchargée.

L’éparpillement des populations, la taille du territoire, la faible conscience médicale de certaines populations rendent difficile la mise en place d'une politique de prévention et de dépistage [50]. Se heurtant à la réticence d'une partie de la population, la contraception reste peu utilisée en dehors des villes. Il est nécessaire d'adapter le système de prévention aux caractéristiques des patientes et à leur isolement: équipes mobiles, création d'hôtels maternels pour rapprocher les femmes enceintes d'un CH lors des dernières semaines de la grossesse, amélioration des conditions de transport, lutte contre les IST. Amorcée depuis plusieurs années, la coopération avec les pays voisins, Suriname et Brésil, peine à entraîner des réalisations concrètes.

Dans ce territoire français du bout du monde, la prise en charge des grossesses reste un défi malgré les énormes progrès réalisés, et nécessite la mise en place de protocoles adaptés.

## Pathologies Cardiovasculares Et Métaboliques

### Accidents vasculaires cérébraux (AVC)

Bertrand de Toffol

Les AVC sont un problème majeur de santé publique dans le monde. En 2019, ils constituaient la troisième cause de décès et/ou de handicap dans le monde [143] et en France, en 2013, la première cause de décès chez les femmes et la troisième chez les hommes [206]. Dans l'Hexagone, l'incidence des AVC ischémiques était en 2014 de 112,8/100 000 contre 189,5/100 000 en Guyane. Tous types d'AVC confondus, les taux standardisés sont supérieurs de 28% en Guyane par rapport à l'Hexagone [205] et leur taux de mortalité (72/100 000) y est 2 fois plus important [206]. Les patients admis pour un infarctus cérébral en Guyane diffèrent de ceux de l'Hexagone sur de nombreux aspects: les patients guyanais sont plus jeunes (en moyenne 62 vs 70 ans), et les facteurs de risque ont une répartition différente: obésité (18,0% vs 11,7%), diabète (7,7% vs 4,6%), hypertension artérielle (40,0% *vs* 18,6%) [23, 314, 351]. Il en résulte une sur-représentation des accidents lacunaires, complication classique de l'association d'une hypertension et d'un diabète [24, 308], alors que les AVC ischémiques de cause cardio-embolique semblent moins fréquents [23]. Ces caractéristiques épidémiologiques sont retrouvées dans les pays frontaliers de la Guyane, au Brésil [55] et au Suriname [12]. Cependant, un certain nombre d'AVC sont considérés comme de cause indéterminée en Guyane car les moyens d'exploration cardiaque et les ressources en cardiologues sont limités. Des nombreux patients quittent le service de neurologie (qui n'existe de manière autonome que depuis 2019) sans avoir eu d’échographie cardiaque ou le résultat de l'enregistrement Holter. Les facteurs qui rendent compte de l'incidence et de la prévalence des AVC sont nombreux et en interaction. La précarité, les facteurs ethniques, nutritionnels et culturels ont été étudiés en Guyane. Dans l’étude India, la précarité et un bas niveau d’éducation étaient deux déterminants significatifs de la sévérité des AVC [23]. La précarité rend également compte d'une moins bonne détection et prise en charge des facteurs de risque [317]. L’étude des facteurs génétiques et ethniques a montré que les populations d'origine africaine étaient plus à risque d'hypertension [314] et de diabète [11, 377]. La Guyane est un territoire multiethnique et multiculturel. En se basant sur la langue maternelle des patients, l’étude India a montré que la moitié de la population guyanaise victime d'AVC était d'origine étrangère (Haïti 41,2%, Suriname 22,5%, Brésil 10,9%, Guyana 5,8%, Sainte-Lucie 5,1%.) [316]. Il y a deux fois plus de personnes diabétiques en Guyane par rapport à l'Hexagone, et le diabète est souvent mal contrôlé du fait d'un régime inadapté, de la sédentarité et de difficultés à développer l’éducation thérapeutique [351].

Il n'y a pas encore d'Unité neurovasculaire en Guyane (ouverture prévue fin 2022). Le nombre de thrombolyses rapporté au nombre d'AVC est cinq fois moins élevé en Guyane que dans l'Hexagone et la thrombectomie n'est pas disponible.

### Cardiopathies

Loïc Epelboin

Les cardiomyopathies sont un problème clinique fréquent et grave. La fréquence des causes infectieuses et l'atteinte dilatée sont des arguments pour les cardiomyopathies à particularités tropicales. Une étude transversale a été réalisée au CHC portant sur toutes les cardiopathies prises en charge de 2009 à 2014 [208]. Avec 182 patients inclus, la prévalence des cardiomyopathies parmi les maladies cardiaques a été estimée à 4,3%. Le spectre morphologique était: dilatée pour 114 patients (62,6%), hypertrophique pour 27 (14,8%) et mixte pour 27 (14,8%). Douze patients avaient une étiologie familiale ou génétique (6,6%) et 170 une étiologie non familiale ou non génétique (93,4%). En termes étiologiques, 70,1% étaient d'origine indéterminée, 8,7% d'origine infectieuse (dont 7,0% liées à la maladie de Chagas et 0,5% liées au VIH), 6,5% d'origine familiale ou génétique, toxiques dans 6% des cas. Les autres causes étaient plus anecdotiques. Un tiers des patients de l’étude étaient au stade de l'insuffisance cardiaque chronique.

### Coronaropathies

Loïc Epelboin

La Guyane ne dispose pas de cardiologie interventionnelle. La thrombolyse, avant evasan, est donc l'unique traitement étiologique disponible pour la prise en charge du syndrome coronarien aigu ST+ avec sus-décalage du segment ST (SCA ST+). Une étude a été réalisée à partir d'une cohorte rétrospective descriptive hospitalière au CHC entre 2012 et 2014 [315]. Parmi les 266 patients, l’âge moyen était de 64 ans, et le sex-ratio 1,83. La proportion de patients nés dans un territoire français d'outre-mer (44%) était similaire à celle des patients nés dans un pays étranger (44%), et 12% étaient nés en France hexagonale. Les principaux facteurs de risque étaient l'HTA (74%), le diabète (40%), l'hypercholestérolémie (40%) et le tabagisme (38%). La thrombolyse n'a été pratiquée que chez 21% des patients. La mortalité à un mois était de 8/82 (10%) pour les patients avec un ST+, similaire à celle du Brésil. Une étude rétrospective et monocentrique portant sur 111 patients d'une moyenne d’âge de 58 ans, pris en charge pour un SCA ST+ de moins de 24 heures sur la période entre 2014 et 2016 au CHC, a montré que le taux de thrombolyse était faible (69,4%) ainsi que le taux de revascularisation de 52,7% [171]. Les délais de prise en charge et d'evasan étaient longs: le délai de consultation médian était de 1 heure et 44 minutes, et le délai douleur-thrombolyse de 3 heures. Soixante-neuf pour cent des patients ont bénéficié d'une evasan dans un délai médian de 36 heures. Le taux de mortalité à 24 heures était de 7,2% contre 2,6% dans l'Hexagone. Le taux d'appel au SAMU était de 50,9% (76% en Île-de-France). Si une sensibilisation de la population guyanaise ainsi qu'une optimisation des pratiques médicales locales peuvent l'améliorer, la gestion du SCA ST+ en Guyane ne saurait être comparable à celle des autres départements français tant que le territoire guyanais ne disposera pas de son propre plateau de coronarographie interventionnelle.

### Diabète sucré

Nadia Sabbah

Le diabète sucré en Guyane est un problème prioritaire de santé publique dont la prévalence augmente de manière inquiétante (plus de 10% de la population entre 2018 et 2019), parallèlement à celle de l'obésité et du surpoids (cf. section suivante sur l'obésité) [323, 351]. La transition épidémiologique en Amazonie française entre les maladies infectieuses et les maladies métaboliques est largement réalisée, mettant au premier rang de la morbi-mortalité les pathologies métaboliques et cardiovasculaires [316]. Les dernières estimations de prévalence du diabète sucré en Guyane retrouvaient en 2014 sur les données du baromètre santé un chiffre de 9,3%, et plus récemment (2019) Fuentes *et al.* retrouvaient chez les personnes de plus de 45 ans, respectivement 14 et 20% chez les hommes et les femmes, soit presque le double de la moyenne nationale [137, 324]. Contrairement aux autres territoires de France hexagonale, on note une large prédominance féminine (tout comme pour l'obésité) expliquée probablement par les difficultés sociales, la sédentarité et les problèmes d'accès aux soins qui sont majorés chez les femmes [311]. Les prises en charge éducative et thérapeutique sont complexes eu égard aux différentes cultures, à l'importance de l'illettrisme, à l'isolement géographique d'une partie de la population avec un accès limité aux hôpitaux, et à la forte précarité [323]. En effet, les plus de 22 groupes ethniques qui composent le territoire ont chacun leurs croyances, leur langue, et les méthodes éducatives doivent prendre en compte cet aspect, mettant ainsi en exergue l'importance des médiateurs en santé issus des communautés afin de faire passer les informations et messages essentiels. Plusieurs publications mettent en évidence le lien entre mauvaise observance, équilibre glycémique et difficultés de compréhension par les patients [266]. En Guyane, l'accès aux soins est parfois compliqué et le renoncement à celui-ci est important, la faible densité médicale participant à cette problématique. Une publication récente retrouvait un décalage très important entre les recommandations nationales de suivi des patients ayant un diabète sucré et ce qui était effectivement réalisé sur le territoire. Cette étude notait qu'en fonction de la situation géographique il existait de grandes disparités dans le suivi, avec de manière attendue des difficultés pour les patients situés dans les zones forestières isolées nécessitant parfois plusieurs jours de pirogue ou de piste pour accéder au premier centre de santé ou hôpital. Elle retrouvait également un faible contrôle des examens essentiels: 19% des patients diabétiques réalisaient un fond d’œil annuel, 56% un contrôle de l'HbA1C (hémoglobine glyquée) tous les six mois, 43% effectuaient leur bilan rénal annuel et 25% seulement réalisaient un bilan dentaire annuel [351].

L'isolement géographique et la précarité (avec parfois l'absence de droits sociaux) sont également des freins à l'application des recommandations nationales, notamment lors de la mise à l'insuline où les difficultés de conservation (du fait de l'absence d’électricité) de celle-ci s'additionnent au risque d'erreur de dose par mauvaise compréhension. Certains quartiers dits « informels » et certaines zones du territoire sont difficiles d'accès pour les infirmières à domicile et les patients sont parfois seuls dans la gestion de leur traitement. L'enjeu des équipes soignantes est d'essayer de mettre en place des traitements adaptés au mode de vie et à la situation sociale des patients, tout en essayant de garder un objectif glycémique le plus proche possible d'une cible réalisable et acceptable.

La prise en charge du diabète en Guyane est un objectif de santé majeur du territoire, qui nécessite une coordination parfaite de tous les acteurs impliqués et surtout un maillage du territoire complet afin d'atteindre les populations les plus isolées qui sont souvent les plus compliquées en terme glycémique. Une filière ville/hôpital spécialisée a ainsi débuté en 2020 avec l'aide d'un outil de télémédecine associant de l’éducation thérapeutique mobile, des médiateurs culturels, des consultations diététiques gratuites en ville, la prise en charge de séances d’éducation physique avec un éducateur sportif et le déploiement de formations des soignants en diabétologie et pathologies métaboliques sur l'ensemble du territoire. La recherche clinique se développe également, constituant un axe important d'amélioration de la prise en charge des patients grâce à une meilleure connaissance de la maladie sur le territoire.

### Obésité

Nadia Sabbah

En Guyane, l'obésité est une priorité de santé publique et représente l'un des principaux facteurs de risque cardiovasculaires associés au diabète et à l'hypertension artérielle. Les pathologies cardiovasculaires sont au niveau du territoire la première cause de mortalité, en particulier liées aux accidents vasculaires cérébraux souvent précoces (cf. paragraphe supra) [316]. Malgré une prévalence très élevée des pathologies métaboliques en Amazonie française, il existe peu de littérature sur le sujet. On note à partir des données du baromètre santé 2014 une prévalence respectivement d'environ 19% et 36% d'obésité et de surpoids dans la population adulte, avec une prédominance féminine forte et une obésité de l'enfant qui tend à augmenter depuis l’étude PODIUM (2008) qui retrouvait déjà une prévalence à plus de 6% [77, 222]. L'obésité est une maladie multifactorielle influencée par une prédisposition génétique, un mode de vie sédentaire, un apport alimentaire accru ou mal équilibré, et des facteurs psychologiques et socio-économiques. Les inégalités sociales sont très fortes en Guyane avec un taux de précarité très important par rapport aux autres territoires de France, occasionnant des difficultés dans la prise en charge éducative et thérapeutique des pathologies métaboliques [369]. Les inégalités de santé sont favorisées par des difficultés d'accès aux soins majeures liées aux infrastructures routières peu développées surtout pour les territoires les plus à l'intérieur, à la faible densité médicale, et à la précarité. La Guyane est un territoire complexe dont la diversité culturelle fait partie de ses plus grands atouts mais constitue également l'une de ses problématiques en particulier dans la prévention et la prise en charge des pathologies métaboliques. L'aspect culturel est en effet un point majeur dans la mise en place des stratégies de prise en charge. En Guyane, la perception du surpoids est souvent faussée, et on retrouve souvent un décalage entre l'image corporelle et le surpoids/obésité, ce qui constitue un obstacle majeur à l'amélioration des comportements alimentaires et du mode de vie. Une étude récemment publiée mettait en évidence une mauvaise perception de l'image corporelle chez plus de 75% des personnes en surpoids ou obèses en Guyane, qui se définissent juste comme étant « un petit peu trop gros » [222]. Cette étude retrouvait également que les femmes, célibataires, issues de l'immigration, avec peu d'instruction scolaire et sans emploi étaient les plus touchées par l'obésité [222]. L'enjeu dans la prise en charge de l'obésité et du surpoids en Guyane réside dans la mise en place d'une filière graduée coordonnée entre la ville et l'hôpital et surtout en lien avec les structures de proximité (notamment les Centres délocalisés de prévention et de soins) en tenant compte des particularités sociales, culturelles, et de l'isolement géographique d'une partie de la population. Un projet de filière de chirurgie bariatrique est en cours de mise en place.

## Cancers Et Hémopathies Malignes

Jean-Pierre Droz

### Données épidémiologiques

#### Incidence et mortalité

**Figure 69:**
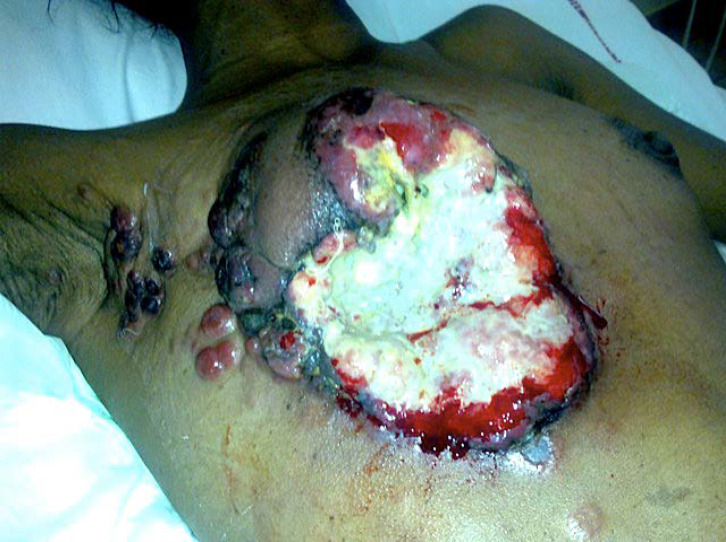
Patiente chinoise de 37 ans habitant Cayenne. Cancer du sein évoluant depuis 2 à 3 ans (caché, déni) et métastatique (os et foie). Carcinome canalaire infiltrant avec récepteurs hormonaux positifs et HER2 négatif (crédit photo: J.-P. Droz) Traitement par chimiothérapie 37-year-old Chinese woman living in Cayenne. Breast cancer evolving since 2 to 3 years (hidden, denial) and metastatic (bone and liver). Invasive ductal carcinoma with hormone receptor positive and HER2 negative (photo credit: J.-P. Droz) Chemotherapy treatment

**Figure 70:**
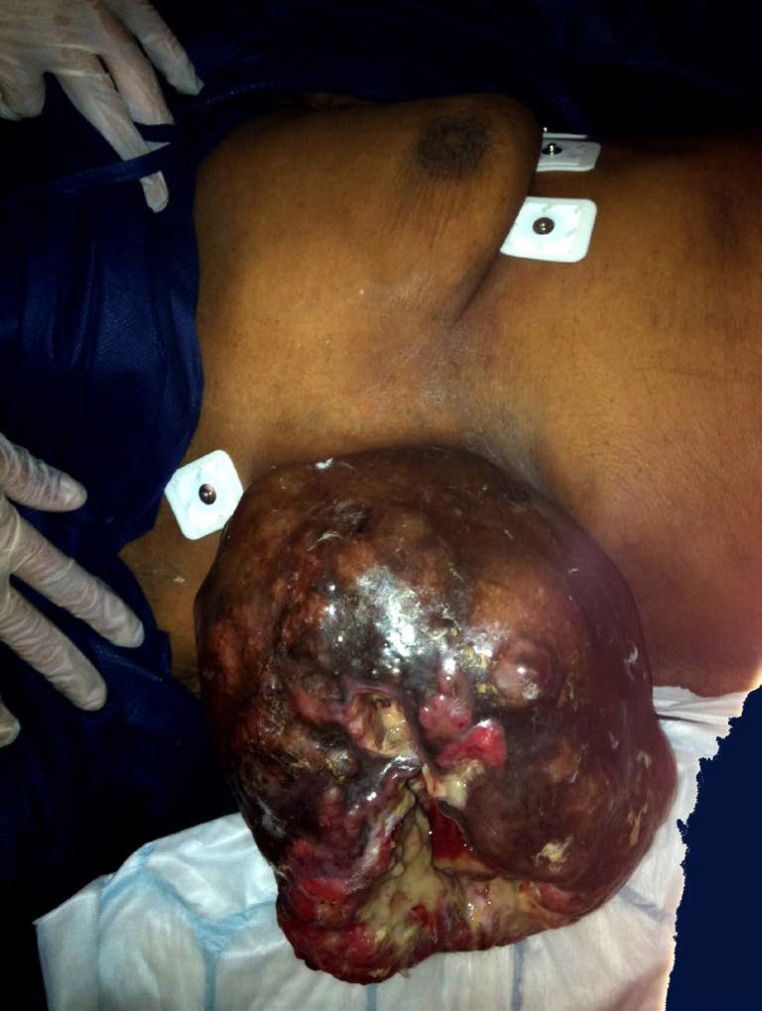
Patiente créole de 70 ans habitant Kourou, atteinte de cancer du sein droit classé T4BN0M1 (os, poumon). Carcinome canalaire infiltrant avec récepteurs hormonaux positifs et HER2 négatif (crédit photo: J.-P. Droz) Traitement par mastectomie, chimiothérapie et evasan dans l'Hexagone pour radiothérapie et chirurgie de consolidation rachidienne (métastases osseuses) 70-year-old Creole woman living in Kourou with right breast cancer classified T4BN0M1 (bone, lung). Invasive ductal carcinoma with hormone receptor positive and HER2 negative (photo credit: J.-P. Droz) Treatment by mastectomy, chemotherapy and medical evacuation to the Hexagon for radiotherapy and spinal consolidation surgery (bone metastases)

**Figure 71:**
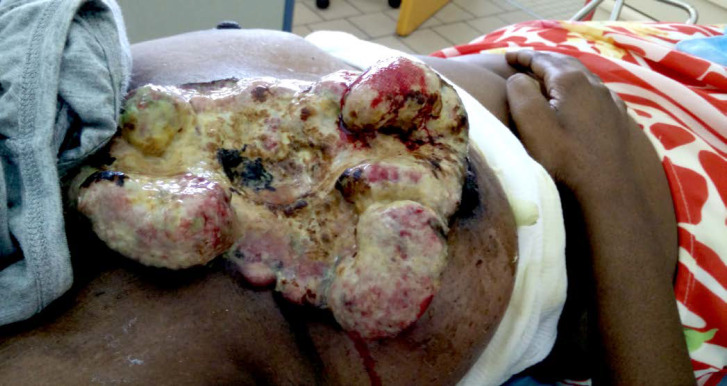
Patiente ndjuka, de nationalité surinamaise, de 45 ans habitant Mana (près de Saint-Laurent-du-Maroni) atteinte d'un cancer du sein T4dN1M0. Carcinome canalaire infiltrant avec récepteurs hormonaux et HER2 positifs (crédit photo: J.-P. Droz) Traitement par chimiothérapie et anticorps monoclonaux contre HER2 et inhibiteur de l'aromatase, puis prise en charge au Suriname pour radiothérapie de la paroi thoracique (réponse complète) 45-year-old Ndyuka woman, Surinamese nationality, living in Mana (near Saint-Laurent-du-Maroni), with T4dN1M0 breast cancer. Invasive ductal carcinoma with hormone receptor and HER2 positive (photo credit: J.-P. Droz) Treatment by chemotherapy and monoclonal antibodies against HER2 and aromatase inhibitor, then taken to Suriname for chest wall radiotherapy (complete response)

**Figure 72:**
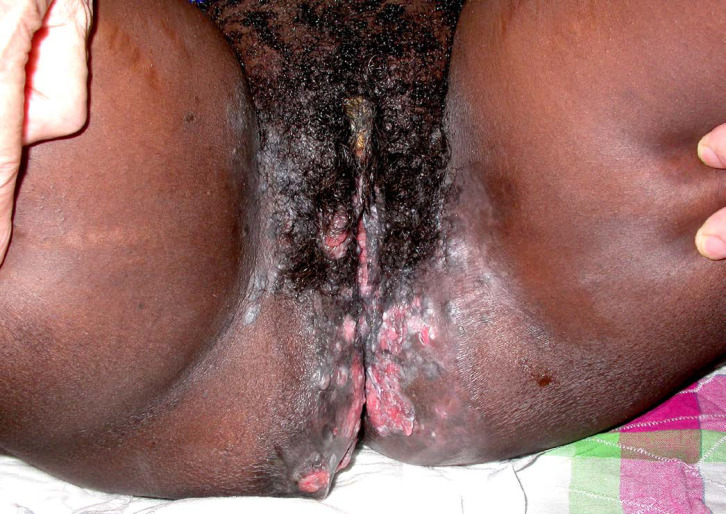
Patiente ndjuka de 29 ans, surinamaise, habitant Saint-Laurent-du-Maroni, VIH+ connue depuis l’âge de 17 ans, toxicomane au cannabis. Elle est atteinte d'une condylomatose HPV+ de tout le périnée avec des zones de carcinome in situ et micro-infiltration (crédit photo: J.-P. Droz) Traitement par chirurgie d'exérèse cutanée locale (avec autogreffe), contrôle de l'infection VIH. Développement d'un carcinome épidermoïde de la vulve traité par radiochimiothérapie (evasan dans l'Hexagone). Puis développement de métastases. 29-year-old Ndyuka Surinamese woman living in Saint-Laurent-du-Maroni, HIV+ known since the age of 17, cannabis addict. She has HPV+ condylomatosis of the entire perineum with areas of carcinoma in situ and microinfiltration. Treatment by local skin excision surgery (with autograft), control of HIV infection (photo credit: J.-P. Droz) Development of squamous cell carcinoma of the vulva treated with radiochemotherapy (medical evacuation to the Hexagon). Followed by development of metastases.

**Figure 73:**
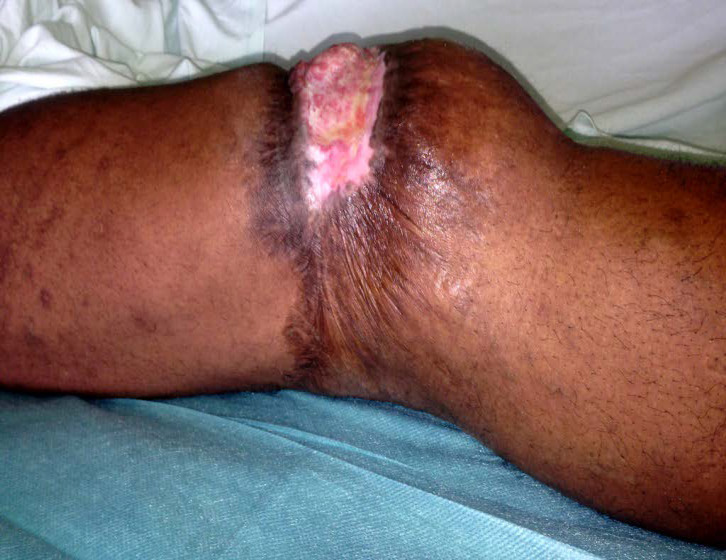
Patient brésilien de 58 ans, sans aucune prise en charge médico-sociale ni administrative, orpailleur sur le Maroni. Carcinome spinocellulaire infiltrant sur ancienne cicatrice de traumatisme de la cuisse droite audessus du genou, ganglion du triangle de Scarpa lié à une mycose (crédit photo: J.-P. Droz) Sur le scanner, découverte d'une passe surrénale droite qui évoque un incidentalome. En IRM, atteinte des muscles de la loge antérieure de la cuisse et de la partie supérieure de la loge antéro-externe de la jambe. Surrénalectomie montrant une métastase nécrosée du carcinome épidermoïde puis amputation tiers de cuisse. 58-year-old Brazilian gold digger on the Maroni, without any medical, social or administrative care. Invasive squamous cell carcinoma on old trauma scar of the right thigh above the knee, Scarpa's triangle lymph node related to a mycosis (photo credit: J.-P. Droz) On the CT scan, discovery of a right adrenal pass that suggests an incidentaloma. *On MRI, involvement of the muscles of the anterior thigh and the upper part of the antero-external bed of the leg. Adrenalectomy showing necrotic metastasis of squamous cell carcinoma and third of thigh amputation.*

Pendant la période 2007-2014, le Registre des cancers de Guyane a enregistré en moyenne 456 nouveaux cas (54% chez l'homme) et 128 décès par an [57]. Les taux standardisés (monde) d'incidence (259/100 000) et de mortalité (79/100 000) sont inférieurs à ceux de l'Hexagone [176]. L’âge de survenue est aussi plus jeune (surtout chez la femme). Le « top 5 » en incidence est: sein, col de l'utérus, colon-rectum, thyroïde, poumon chez la femme et prostate, colon-rectum, poumon, estomac, lymphomes non hodgkiniens (LNH) chez l'homme. Par rapport à l'Hexagone, on observe une sur-incidence des cancers de l'estomac dans les deux genres et chez la femme une sur-incidence du cancer du col de l'utérus et du myélome multiple. Le diagnostic du cancer est souvent tardif et le stade avancé localement ou même métastatique, par exemple le cas du cancer du col (44% des patientes) [101], mais aussi le cancer du sein et de l'estomac (Fig. 69, 70, 71, 72, 73). Le « top 4 » des hémopathies malignes sont les LNH, dont les leucémies/lymphomes à cellules T de l'adulte (ATL), le myélome multiple et la leucémie myéloïde chronique [103].

#### Facteurs de risque

Beaucoup de cancers en Guyane sont liés à des micro-organismes [82]: les génotypes oncogéniques du virus HPV (cancer du col, de l'oropharynx, de l'anus, vulve, verge), virus des hépatites B et C (hépatocarcinome, LNH), virus EBV (LNH, maladie de Hodgkin, cancer indifférencié du naso-pharynx), virus HHV8 (sarcome de Kaposi et tumeur de Merkel), virus HTLV-1 (ATL), *Helicobacter pylori* (cancer de l'antre gastrique, LNH gastriques). Par ailleurs, l'incidence de l'infection VIH chez les patients atteints de pathologies malignes est élevée (6 à 9% – résultats personnels). Les cancers classant sida sont: LNH, maladie de Kaposi et cancer du col. Beaucoup de ces cancers sont viro-induits. Enfin, surpoids et obésité sont des facteurs de risque importants en Guyane [57].

### Spécificités de la prise en charge

#### Moyens de prise en charge

Le diagnostic anatomo-cytopathologique est assuré incluant l'immunohistochimie, quelques hybridations *in situ*, cependant la biologie moléculaire doit être réalisée dans l'Hexagone. L'imagerie courante est assurée (scanner, IRM, échographie, mammotome, certains actes de radiologie interventionnelle) mais le TEP scan n'est pas accessible en Guyane (pour le bilan d'extension, l’évaluation thérapeutique et éventuellement le centrage en radiothérapie). La chirurgie de base du cancer est accessible (sénologie, chirurgie pelvienne, chirurgie viscérale digestive) mais il n'est pas possible de faire sur place la neurochirurgie, la chirurgie thoracique, celle des sarcomes osseux et des parties molles, ni les chirurgies digestives complexes (foie et pancréas) ni enfin la chirurgie pédiatrique. Le territoire ne dispose d'aucune structure de radiothérapie, ni à visée curative ni à visée palliative. Tous les traitements médicaux (chimiothérapie, thérapeutiques ciblées, immunothérapie) sont accessibles dans les trois établissements hospitaliers. Les soins oncologiques de support sont accessibles, y compris les SSR (soins de suite et de réadaptation) et soins palliatifs, mais trois aspects sont fragiles: suivi des effets graves de l'immunothérapie, accueil des urgences oncologiques, réseau ville-hôpital à cause du déficit de médecins et de soignants.

L'hématologie est difficile à prendre en charge faute de moyens diagnostiques complexes, de recours à la réanimation hématologique, de thérapie cellulaire (greffe de cellules souches) [103]. Les évacuations sanitaires concernent donc l'indication de radiothérapie, de chirurgie complexe, de thérapies cellulaires, une grande partie de l'hématologie et entièrement l'oncopédiatrie.

#### Contexte socio-culturel

La Guyane est une mosaïque culturelle, la précarité et l'illettrisme sont beaucoup plus fréquents que dans l'Hexagone, beaucoup de patients n'ont pas de titre de séjour en règle et/ou de prise en charge médico-sociale, ce qui induit de grandes inégalités d'accès aux soins. Par exemple, la « consultation d'annonce » rendue obligatoire par l'Institut national du cancer (INCa) doit être adaptée au contexte de la culture businenge [42], l'outil de dépistage G8 de la fragilité des personnes âgées est plutôt inadapté et peu discriminant [185], certains patients ne peuvent pas bénéficier d'une évacuation sanitaire par absence de documents médico-administratifs.

#### Perspectives

Le dépistage des cancers, le diagnostic précoce, même s'ils sont efficients sur le littoral, doivent être développés dans les zones isolées (particulièrement les problématiques du cancer du col, du sein, de la prostate, les pathologies liées aux virus VIH et HTLV-1). La prévention doit être une priorité de santé publique avec les vaccinations HPV et VHB, la prise en charge précoce des gastrites à *H. pylori*, la lutte contre le surpoids. Une réflexion puis un plan d'action doivent être mis en place pour adapter les dispositifs de l'INCa aux patients de culture non occidentale (Businenges, Amérindiens, Haïtiens, etc.). Enfin, il faut développer l'offre médicale et de soins, une vision « santé globale » et une lutte contre les inégalités demandée par l'INCa. En somme, on peut dire que la Guyane est un laboratoire de la cancérologie tropicale.

## Recommandations Aux Voyageurs

### Voyageurs vers la Guyane

Nicolas Vignier, François Delon, Loïc Epelboin

La Guyane est une destination touristique encore balbutiante pour les voyageurs originaires des zones tempérées, mais la question des recommandations aux voyageurs au départ de l'Europe se pose de plus en plus souvent.

#### Protection personnelle antivectorielle (PPAV)

La protection antivectorielle est souhaitable pour se prémunir du risque d'arbovirose, mais n'est pas toujours aisée à mettre en œuvre la journée – *Ae. aegypti* est un vecteur diurne – au vu du climat chaud et humide. Elle est en revanche plus souvent recommandée pour se prémunir de la leishmaniose et du paludisme dans les rares zones où il persiste. Ainsi la PPAV passe par l'utilisation de répulsifs à dosage suffisant, le port de vêtements longs et l'utilisation de moustiquaires notamment pour les hamacs en forêt.

#### Chimioprophylaxie antipaludéenne

La dernière carte de répartition des cas de paludisme remontant à 2018 (Fig. 20) montre globalement de nombreux fleuves en rouge et le littoral préservé. La situation a beaucoup évolué au cours des dernières années et, bien que le vecteur soit présent, le risque résiduel de paludisme est devenu très faible et ne devrait plus faire recommander un traitement préventif, y compris pour les voyageurs ou les professionnels se rendant dans les communes isolées (contrairement à ce que pourraient laisser entendre les recommandations françaises) [334]. Une prophylaxie n'apparaît donc nécessaire qu'en cas de déplacement dans des zones à risque, en situation d'isolement en forêt ou dans des conditions de séjour à risque particulier, notamment les militaires. Cette position simplificatrice ne devra cependant pas empêcher de rechercher le paludisme devant toute fièvre en Guyane ou au retour de Guyane.

#### Vaccinations

Mise à part la mise à jour des vaccins selon le calendrier vaccinal en vigueur, le principal vaccin à prévoir est celui contre la fièvre jaune qui, bien que rarissime, a été rapportée récemment en Guyane [362]. La vaccination avec une dose unique est obligatoire pour entrer sur le territoire et est vérifiée à l'aéroport au départ (carnet « jaune » conforme au règlement sanitaire international). L'OMS ne recommande plus qu'une dose, là où certains experts pensent qu'une deuxième dose après 10 ans est nécessaire pour garantir une protection à l’échelle de la vie [273]. Bien que l'Amazonie soit une zone d'endémie pour l'hépatite A, le risque semble faible en Guyane probablement en lien avec un niveau de salubrité satisfaisant. La vaccination n'est ainsi pas recommandée sauf dans des situations particulières, notamment pour les personnes allant travailler dans les communes isolées (soignants, enseignants, etc.). Elle peut cependant être utile chez les personnes qui voyagent régulièrement sous les tropiques et une consultation pré-voyage peut être l'occasion de la proposer (2 doses à 6 mois d'intervalle). Le risque de fièvre typhoïde est également négligeable et, bien que quelques cas aient été rapportés dans des bourgs isolés sur le fleuve Maroni chez des habitants vivant dans des conditions d'hygiène dégradées, la vaccination, peu efficace, n'est pas à recommander chez le voyageur quel que soit son mode de voyage. La vaccination antirabique n'est pas recommandée pour tous mais doit s'envisager en cas d'exposition professionnelle au risque de morsure par des mammifères et par les chauves-souris en particulier. De plus, la vaccination pourra être envisagée pour les voyageurs en ayant les moyens et souhaitant le risque minimal. Les nouveaux schémas simplifiés à deux doses peuvent être utilisés. La vaccination contre la dengue avec le seul vaccin actuellement disponible, le Dengvaxia n'est pas recommandée pour les voyageurs, à fortiori s'ils sont non-immuns, du fait du risque de réaliser une dengue plus grave chez un patient vacciné alors que non-immun. L'arrivée récente d'un nouveau vaccin produit par le laboratoire Takeda semble s'affranchir de ce problème et les recommandations devraient alors évoluer.

#### Risques liés au péril fécal

Sur la grande majorité du territoire guyanais, exception faite des habitats informels du littoral et des « écarts » des communes des fleuves, l'eau du robinet est potable, et les précautions habituellement appliquées dans les pays à faible niveau d'hygiène concernant les glaçons, l'accès à l'eau, la gestion des fruits et crudités, etc, n'ont pas lieu d’être.

#### Recommandations diverses

Forêt: Un bon équipement pour la marche en forêt (chaussures de marche fermées) est nécessaire pour se prémunir du risque de morsure de serpents, qui n'est pas nul en Guyane. Ne pas partir seul en forêt. En plus des risques déjà décrits, il y a le risque de se perdre, le risque de chute d'arbres/branches, le risque de plaies +/- avec corps étrangers à risque de surinfections (épines), etc.

Soleil et chaleur: Protection nécessaire contre les coups de soleil, avec un indice UV souvent ≥ 10. Pour le risque de coup de chaleur, en plus de l'hydratation, envisager une acclimatation progressive à l'effort chez les nouveaux arrivants compte tenu du climat chaud et humide perturbant la thermolyse et la correction des pertes en sel.

IST: au vu de la prévalence du VIH et des IST (recrudescence importante de la syphilis), les rapports sexuels protégés sont recommandés et la prescription de la prophylaxie pré-exposition au VIH (PrEP) peut s'envisager pour les voyageurs les plus à risque. Le traitement post-exposition est quant à lui disponible dans la plupart des structures de santé du territoire.

#### Risques divers

Les risques d'accident de la route ou de pirogue, et de noyade sont réels et doivent être considérés, ainsi que le risque de coup de chaleur et de déshydratation. L'accès aux soins et aux médicaments en pharmacie est correct en Guyane, en particulier sur le littoral.

### Au départ et au retour de Guyane

Victor Tertre, Nicolas Vignier, François Delon, Loïc Epelboin

**Bilan de départ de Guyane chez des patients réalisant des séjours prolongés en forêt**

Chaque année, des personnes quittent la Guyane, parmi lesquelles des personnes ayant réalisé des séjours prolongés en forêt, comme les militaires, les scientifiques, les soignants et d'autres catégories socio-professionnelles, sans forcément de suivi médical. La grande variété de pathologies présentes sur le territoire, et le manque de connaissances les concernant, sont parfois source de certaines craintes. Il peut alors être envisagé de réaliser une consultation médicale de pré-départ ou au retour de la Guyane.

#### Interrogatoire et examen clinique

L'interrogatoire doit rechercher les pathologies et symptômes survenus depuis l'arrivée en Guyane. L'examen clinique portera une attention particulière à l'examen dermatologique ainsi que sur les signes fonctionnels et l'examen clinique digestif.

#### Bilan biologique

Un bilan biologique peut être proposé, afin de dépister certaines pathologies initialement asymptomatiques, mais dont l'expression clinique peut être plus tardive. Hémogramme: intéressant pour rechercher une hyperéosinophilie ou une anémie qui peuvent être liées à une parasitose intestinale.

Examen parasitologique des selles 3 jours consécutifs pour rechercher une infection par ankylostome, anguillule, et/ou une giardiase. La sérologie anguillulose peut également être utilisée [126]. Un déparasitage systématique par ivermectine en dose unique et albendazole 3 jours consécutifs peut être envisagé de façon systématique chez une personne ayant réalisé des séjours prolongés et/ou répétés en forêt.

Sérologie fièvre Q: en cas d'histoire clinique évocatrice de pneumopathie ou de fièvre inexpliquée au cours du séjour, elle peut être proposée de façon à réaliser un suivi à la recherche d'une chronicisation, en particulier chez un patient immunodéprimé ou porteur de valvulopathie.

Sérologie Chagas: peut être proposée chez des personnes ayant séjourné de façon prolongée dans les communes isolées.

Bilan d'IST: à proposer systématiquement si des rapports sexuels non protégés ont eu lieu au cours du séjour: sérologies VIH, VHB, VHC, syphilis et HTLV-1 et recherche de gonocoque et de chlamydia dans les urines, par auto-prélèvement vaginal, au niveau anal et/ou pharyngé selon les pratiques.

## Algorithmes Décisionnels

Mathilde Boutrou, Richard Naldjinan Kodbaye, Philippe Abboud, Céline Michaud, Gaëlle Walter

**Figure 74:**
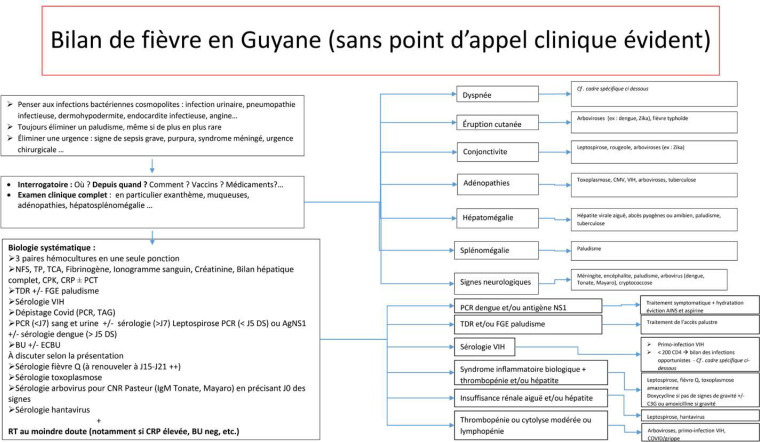
Algorithme décisionnel devant une fièvre chez un adulte immunocompétent vivant en Guyane ou en revenant Decision-making algorithm for fever in an immunocompetent adult living in or returning from French Guiana

**Figure 75:**
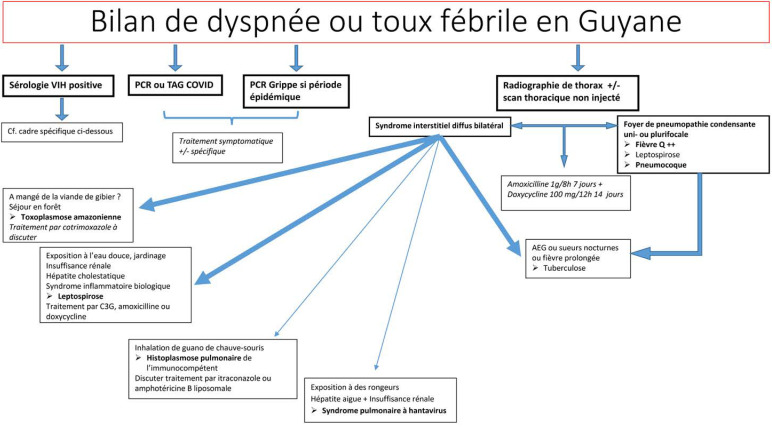
Algorithme décisionnel devant une fièvre chez un patient adulte vivant avec le VIH habitant en Guyane ou en revenant Decision-making algorithm for fever in an adult patient living with HIV residing in or returning from French Guiana

**Figure 76:**
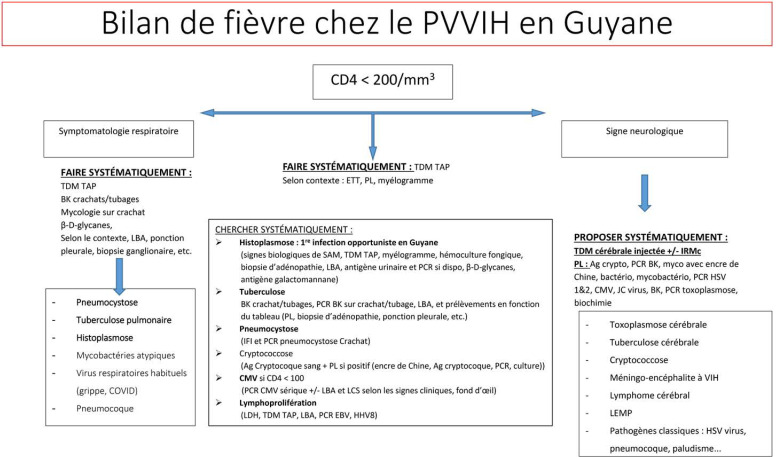
Algorithme décisionnel devant une fièvre chez un adulte immunocompétent vivant en Guyane ou en revenant Decision-making algorithm for fever in an immunocompetent adult living in or returning from French Guiana

Des situations cliniques fréquentes pour un infectiologue nécessitent une approche spécifique chez des patients vivant ou revenant de Guyane. Sont proposés ici 3 algorithmes décisionnels:

Conduite à tenir devant une fièvre chez un adulte immunocompétent vivant ou revenant de Guyane (Fig. 74);

Conduite à tenir devant des signes respiratoires chez un adulte immunocompétent vivant ou revenant de Guyane (Fig. 75);

Conduite à tenir devant une fièvre chez un patient adulte vivant avec le VIH résidant ou revenant de Guyane (Fig. 76).

## Conclusion

Le souhait des auteurs de ce panorama est que le lecteur, les soignants arrivés ou envisageant de partir exercer en Guyane, mais aussi les soignants prenant en charge un patient en zone tempérée en provenance de l'Amazonie française, aient un outil complet pour ne pas passer à côté de diagnostics essentiels. De nombreuses histoires cliniques circulent sur des patients pris en charge dans l'Hexagone avec des diagnostics faits avec retard: histoplasmose disséminée chez le patient immunodéprimé, ulcère cutané chronique pour lequel personne n'avait évoqué la leishmaniose, pneumopathie communautaire n'ayant pas bénéficié de doxycycline contre la fièvre Q, etc. Le panel des pathologies présentées ici donne un aperçu du quotidien des cliniciens et chercheurs en Guyane, tant sur le littoral, aux urgences, en pédiatrie, en infectiologie, en médecine générale, que dans les centres de santé isolés. Du fait de ressources de niveau européen, il existe des possibilités diagnostiques et thérapeutiques inexistantes dans les pays alentours, et la recherche en Guyane occupe une place clé au sein de la région amazonienne, malgré une population bien plus faible que dans les pays voisins, entraînant un nombre annuel de cas plus faible. Ainsi, certaines pathologies, parmi celles citées ci-dessus, ne sont pratiquement pas décrites dans les pays voisins, probablement du fait d'un sous-diagnostic lié à des ressources plus limitées. La Guyane se trouve donc avoir un rôle moteur dans l’étude de ces pathologies.

Les auteurs espèrent que ce document donnera envie aux cliniciens de l'Hexagone de venir exercer en Guyane, et à ceux qui franchiraient le pas, de se sentir moins seuls au cours de leur exercice clinique, avec ce « panorama de la pathologie tropicale amazonienne ». Il permettra également d'orienter les cliniciens de l'Hexagone devant un patient au retour de Guyane.

L'association de pathologies tropicales variées, de praticiens et chercheurs experts, de moyens diagnostiques et thérapeutiques d'un niveau européen, fait de la Guyane un terrain d'apprentissage de choix pour des étudiants, biologistes, chercheurs et médecins du monde entier qui voudraient se frotter et se former à la pathologie tropicale, le tout dans l'environnement exceptionnel qu'est la forêt amazonienne.

## Remerciements

Merci au photographe Karl Joseph pour les images issues du projet anthropo-photographique Kalalou (financement Labex CEBA).

Ce panorama a été soutenu par l'Union européenne et la Collectivité territoriale de Guyane dans le cadre de l'appel à projet FlashCovid-19 et du projet AMAZCOV'N FEVERS (FEDER, SYNERGIE GY0028034).

Merci à la direction du Centre hospitalier de Cayenne et à l'Agence régionale de santé pour leur aide à la diffusion de cet ouvrage.

Merci à tous les soignants, chercheurs, scientifiques et naturalistes du territoire guyanais, dont les travaux, les écrits et les paroles, et pour certains la prise en charge des patients ont permis l'aboutissement de cet ouvrage. Certains d'entre eux auraient eu leur place parmi les auteurs, mais la taille définitive actuelle de cet ouvrage, ainsi que le nombre de chapitres et d'auteurs sont bien au-delà de ce qui était prévu initialement, actualisation d'un article synthétique de 2016, ce qui explique que certains collègues n'aient pas été ajoutés sur le tard à ce travail.

Enfin, un merci tout particulier à M^me^ Isabelle Borloz, assistante de rédaction à MTSI, pour l’énorme travail qu'elle a fourni pour la mise en page de l'ouvrage, ainsi que pour sa disponibilité et sa réactivité tout au long du processus de publication, et aussi aux membres du Comité de rédaction de MTSI, pour leur impressionnant travail de relecture et pour les nombreuses et judicieuses suggestions et corrections qui ont permis la qualité de ce travail tel qu'il est présenté aujourd'hui.
